# Exploratory proteomic characterization of *Apis mellifera* venom across an environmental gradient in central Chile

**DOI:** 10.3389/fphar.2026.1904856

**Published:** 2026-09-09

**Authors:** José Luis Marcos, Ricardo Campos-Soto, Rina Ortiz, Yerko Olivares, Joan Villena, Mario Párraga, Ramón Sotomayor-Zárate, Luis Constandil, Heine Müller

**Affiliations:** 1 Departamento de Ciencias Veterinarias, Facultad de Ciencias de la Vida, Universidad Viña del Mar, Viña del Mar, Chile; 2 Escuela de Medicina Veterinaria, Facultad Ciencias de La Vida, Universidad Andres Bello, Viña del Mar, Chile; 3 Magíster en Ciencias Médicas mención Biología Celular y Molecular, Escuela de Medicina, Facultad de Medicina, Universidad de Valparaíso, Valparaíso, Chile; 4 Laboratorio de Bioensayos, Escuela de Medicina, Facultad de Medicina, Centro Interdisciplinario de Investigación Biomédica e Ingeniería para la Salud (MEDING), Universidad de Valparaíso, Valparaíso, Chile; 5 Centro de Neurobiología y Fisiopatología Integrativa (CENFI), Instituto de Fisiología, Facultad de Ciencias, Universidad de Valparaíso, Valparaíso, Chile; 6 Laboratory of Neurobiology, Department of Biology, Faculty of Chemistry and Biology, University of Santiago de Chile, Santiago, Chile; 7 Anaesthesiology and Intensive-Care, Clinical Centre for Small Animal Health and Research, Clinical Department for Small Animals and Horses, University of Veterinary Medicine Vienna, Vienna, Austria

**Keywords:** *Apis mellifera*, bee venom proteomics, bioactive peptides, chemical ecology, drug discovery potential, ecological venom variation, LC–MS/MS proteomics, venom pharmacology

## Abstract

**Background:**

Bee venom (*Apis mellifera*) is a complex biological secretion rich in peptides, enzymes, and bioactive molecules with recognized pharmacological potential. Environmental conditions may influence venom composition; however, geographic variation in the bee venom proteome remains insufficiently characterized, particularly in South American Mediterranean ecosystems, including central Chile.

**Methods:**

Venom from multiple *A. mellifera* colonies within each sampling area of the Valparaíso Region, Chile, was pooled to generate one composite sample per geographic zone: Sea Level, Inland Valley, and Andean Foothill. Two analytical aliquots from each pooled sample were independently processed by liquid chromatography tandem mass spectrometry (LC-MS/MS). Protein identification and label-free quantification were performed using MaxLFQ and iBAQ, and differential abundance was assessed using DEqMS.

**Results:**

Overall, 679 proteins were identified. Principal component analysis and hierarchical clustering showed reproducible grouping of analytical duplicates by geographic origin, indicating distinct proteomic profiles among zones. Differential abundance analysis identified proteins with significant abundance changes between regions, particularly between Sea Level and Inland Valley. Classical venom components, including melittin and phospholipase A2, predominated across samples, whereas proteins associated with stress response, redox regulation, metabolism, and secretion showed geographic variation.

**Conclusion:**

Bee venom from geographically distinct areas of central Chile exhibited exploratory proteomic differences, primarily in protein abundance patterns. Although the absence of independent biological replicates limits population-level inference, these patterns suggest environmentally associated proteomic plasticity potentially relevant to biological activity and pharmacological standardization. Further studies incorporating biological replication, targeted validation, and functional assays are required to confirm these findings.

## 1 Introduction

The venom of the honeybee (*Apis mellifera*) is a complex defensive secretion produced by female workers as part of colony protection. It is synthesized primarily by the acid venom gland and the Dufour gland and consists of a mixture of peptides, enzymes, biogenic amines, and other bioactive compounds (Zlotkin, 1997; Alcalá-Escamilla et al., 2024). Bee venom (BV) is a transparent, acidic aqueous secretion rich in biologically active molecules that contribute to both its toxicological effects and pharmacological potential (Khalil et al., 2021; Alcalá-Escamilla et al., 2024). It is composed mainly of water (∼88%) (Gajski et al., 2024), with dry components including melittin, phospholipase A_2_ (PLA2), apamin, and hyaluronidase, which are responsible for inflammatory, neuroactive, and cytotoxic effects while also attracting attention as pharmacologically relevant compounds (Valderrama, 2003; Carpena et al., 2020).

Interest in BV has expanded from traditional apitherapy toward molecular pharmacology and drug discovery. Historically used in complementary medicine for musculoskeletal and inflammatory disorders (Lee et al., 2008; Zhang et al., 2018), BV and its derived peptides have demonstrated anti-inflammatory, analgesic, antimicrobial, neuroprotective, and anticancer effects in experimental models (Baek et al., 2006; Wehbe et al., 2019; Shi et al., 2022). Melittin and apamin, in particular, have been investigated for their roles in the modulation of neuroinflammation, chronic pain pathways, and tumor cell viability, although toxicity, allergenicity, and anaphylactic risk remain major clinical limitations (Burzyńska and Piasecka-Kwiatkowska, 2021; Kuzmenkov et al., 2022). This dual pharmacological–toxicological nature highlights the importance of detailed molecular characterization of venom composition.

Proteomic approaches have become essential for understanding venom variability and identifying bioactive compounds with biomedical potential. Advances in mass spectrometry-based proteomics enable sensitive detection and quantification of venom proteins, complementing genomic resources such as the fully sequenced *A. mellifera* genome, and facilitating biomarker discovery and pharmacological exploration (Honeybee Genome Sequencing Consortium, 2006; Peiren et al., 2005; Banks et al., 2000; Bessarabova et al., 2012). Quantitative analysis of major venom peptides, particularly melittin and phospholipase A_2_, can also contribute to the assessment of venom purity, compositional consistency, and biochemical variability across samples (Peiren et al., 2005; Van Vaerenbergh et al., 2014).

Ecological factors may significantly influence venom composition. Variables such as temperature, floral availability, altitude, seasonality, colony condition, and defensive behavior have been associated with compositional variability in BV (Scaccabarozzi et al., 2021). More broadly, studies on venomous animals suggest that toxin composition can adaptively respond to ecological pressures, influencing defensive strategies, ecological fitness, and biological interactions (Gangur et al., 2017; Surm and Moran, 2021; Koludarov et al., 2023). Evidence from hymenopterans further indicates that altitude and environmental stressors may alter defensive venom potency, suggesting environmentally driven plasticity in toxin expression (Barkan et al., 2019).

Despite increasing pharmacological interest in BV, geographic variation in the proteomic profile of *A. mellifera* venom remains insufficiently explored, particularly in Mediterranean ecosystems of South America. Understanding environmentally driven proteomic variation could provide insights into venom ecology while identifying region-specific bioactive profiles relevant for pharmacological research. Accordingly, the present study evaluates variation in the proteomic profile of *A. mellifera* venom across three contrasting agroecological landscapes in the Valparaíso Region of Chile (Sea Level, SL; Inland Valley, IV; and Andean Foothill, AF). As a working hypothesis, we propose that altitude and its associated environmental conditions may contribute to the observed variation in the relative abundance of venom proteins across geographic regions. However, given the descriptive nature of the study and the absence of independent biological replicates, the observed proteomic patterns cannot be attributed to altitude or specific environmental factors. Rather, these findings should be considered preliminary and hypothesis-generating, providing a basis for future studies incorporating independent biological replication, controlled assessment of environmental variables, and targeted functional validation.

## 2 Methods

### 2.1 Geographic locations of the study sites and climatic characteristics

BV samples from *A. mellifera* were collected from three geographic locations in the Valparaíso Region of central Chile, representing a coastal–valley–Andean altitudinal gradient (Supplementary Figure S1). This region is characterized by a Mediterranean-type climate, with winter precipitation and a prolonged summer dry season, influenced by the subtropical anticyclone and mid-latitude storm tracks (Garreaud et al., 2020). The coastal site (Sea Level; SL) was located in Viña del Mar (33°01′ S, 71°35′ W), where coastal Mediterranean conditions include moderate temperatures, marine influence, and winter rainfall regimes typical of central Chile (Armesto and Arroyo, 2007). The IV site was located near La Ligua (32°27′ S, 71°16′ W) at approximately 200 m above SL, an area characterized by semi-arid Mediterranean conditions, with pronounced atmospheric dryness and strong seasonal precipitation concentration (Becerra, 2016). The AF site (pre-Andean) was located near Putaendo (32°30′ S, 70°43′ W) at approximately 800 m above SL. Increasing elevation in central Chile is associated with greater thermal amplitude, reduced precipitation reliability, and ecological transitions toward montane Mediterranean environments (Salazar et al., 2024).

### 2.2 Venom extraction method

For each geographic zone, BV obtained from four representative colonies was pooled to generate one composite biological sample per geographic origin (SL, AF and IV), minimizing intra-apiary variability. Two independent analytical duplicates were subsequently prepared from each pooled sample and processed separately for LC-MS/MS analysis, generating analytical duplicates for each geographic condition (total n = 6 LC-MS/MS runs). Therefore, proteomic comparisons were based on analytical duplicates derived from pooled biological material rather than independent biological replicates. Consequently, statistical analyses were used to characterize analytical variability and identify exploratory patterns in relative protein abundance, rather than to support population-level biological inference or establish statistically validated differences among geographic regions.

BV was collected from the three study locations using an electrical stimulation device (Bee Farm Collector®) designed for non-lethal venom harvesting. The system delivered controlled electrical pulses (55 V) for 8 s, followed by 4 s resting intervals, over a total stimulation period of 15 min per hive, following previously described methods (Bucio-Villalobos and Martínez-Jaime, 2019). BV was delivered through glass collector plates, inducing venom release without stinger loss or insect mortality. Crystallized venom deposited was recovered from glass plates and transferred into sterile microcentrifuge tubes. Samples were handled under controlled conditions to preserve protein integrity. Samples were carefully scraped, pooled, and weighed using a precision digital balance. Each analytical duplicate consisted of approximately 50 mg of venom suspended in protease inhibitor solution and urea to preserve protein stability. Samples were transported under refrigerated conditions in insulated containers with frozen gel packs and subsequently stored at −20 °C until proteomic analysis to ensure protein integrity. Dry venom yield obtained per zone was SL: 0.9091 g; IV: 2.4057 g; and AF: 0.8888 g.

### 2.3 Proteomic profiling workflow

Proteins were extracted using a lysis buffer supplemented with protease inhibitors, followed by reduction, alkylation, and chloroform/methanol-based cleanup. Sample preparation and proteomic analysis were performed following a protocol based on that described by Vera-Montecinos et al. (2025). Samples were subsequently digested with trypsin, and the resulting peptides were purified using C18 Evotips columns. Proteomic analysis was performed by liquid chromatography coupled to tandem mass spectrometry (LC-MS/MS) using an EVOSEP One (EVOSEP Biosystems) system coupled to a timsTOF Pro mass spectrometer (Bruker) with data-dependent acquisition (DDA) strategy at the MELISA Institute (Concepción, Chile).

Raw mass spectrometry data were processed using FragPipe v19.1 with the MSFragger search engine (Kong et al., 2017). Database search was conducted against the *A. mellifera* reference proteome (The UniProt Consortium, 2023), applying standard parameters for precursor and fragment mass tolerances. Peptide-spectrum matches and protein identifications were filtered using a false discovery rate (FDR) threshold of 1% at both peptide and protein levels. Quantitative information was obtained from MaxLFQ intensity values generated by IonQuant within the FragPipe workflow (Yu et al., 2021). Protein abundance was estimated using both relative label-free quantification (MaxLFQ) and intensity-based absolute quantification (iBAQ). For iBAQ calculation, protein sequences were retrieved from UniProtKB and subjected to *in silico* tryptic digestion using standard cleavage rules, considering theoretical peptides between 6 and 30 amino acids in length. Protein intensities were normalized by the number of theoretically observable tryptic peptides to estimate relative molar abundance.

### 2.4 Statistical analysis

Protein quantification was performed using MaxLFQ intensity values generated by IonQuant within the FragPipe platform. Quantitative data were analyzed in the R statistical environment (R Foundation for Statistical Computing, Vienna, Austria). MaxLFQ intensities were log_2_-transformed to stabilize variance (Cox et al., 2014). Zero intensity values were treated as missing values and converted to NA. Proteins were retained for downstream analyses when at least two quantitative observations were available in at least one geographic condition.

To reduce inter-run technical variability and correct for differences in sample loading and ionization efficiency, data were normalized by median centering of log2-transformed intensities.

Principal component analysis (PCA) was performed using complete quantitative profiles across all analytical runs to evaluate global proteomic variation among geographic conditions. PCA was used to reduce data dimensionality while preserving variance structure, allowing identification of protein expression patterns and clustering of biologically meaningful groups (Jolliffe and Cadima, 2016).

Differential protein abundance (DPA) analysis was conducted using linear models implemented in the limma package. Because the experimental design included only analytical duplicates derived from pooled biological samples, variance estimation was moderated using the DEqMS package, which applies empirical Bayes variance moderation while accounting for peptide-level evidence for each protein. This approach adjusts variance estimates according to peptide coverage and has been proposed to improve statistical reliability in proteomic datasets with limited replication.

Three pairwise contrasts were evaluated: SL *versus* IV, AF *versus* IV, and SL *versus* AF. Proteins were considered significantly differentially abundant when they met both criteria of false discovery rate (FDR) < 0.05 and absolute log_2_ fold-change >1.

To characterize venom composition, absolute abundance estimates were additionally calculated using the intensity-based absolute quantification (iBAQ) method, allowing estimation of the relative molar contribution of major venom components.

## 3 Results

### 3.1 Protein identification and proteome overview

A total of 679 proteins were identified across all pooled venom samples (Supplementary Table S1). Hierarchical clustering of differentially abundant proteins revealed consistent grouping of analytical duplicates according to geographic origin (Supplementary Figure S2), supporting the reproducibility of the proteomic profiles. Geographic variation was mainly reflected by differences in protein abundance patterns among zones, which were further explored through differential abundance analyses. Because protein identification was performed using pooled samples with technical duplicates, these findings should be interpreted descriptively and do not constitute evidence of population-level geographic differentiation.

### 3.2 Relative stoichiometric composition of *Apis mellifera* venom

Absolute stoichiometric quantification based on iBAQ confirmed the characteristic composition of *A. mellifera* venom across all geographic sample groups (Table 1). Melittin was the predominant venom component, representing approximately 72.5%–77.7% of the total proteome, followed by phospholipase A2, which accounted for 6.3%–10.6% of total protein abundance. Hyaluronidase and major royal jelly proteins (MRJPs) were detected at substantially lower proportions, generally contributing less than 2% and 0.4% of the venom proteome, respectively.

**TABLE 1:** Relative stoichiometric composition of Apis mellifera venom determined by iBAQ analysis.

Component	SL_1 (%)	SL_2 (%)	SL mean ± SD (%)	IV_1 (%)	IV_2 (%)	IV mean ± SD (%)	AF_1 (%)	AF_2 (%)	AF mean ± SD (%)
Melittin	72.44	79.94	**76.19 ± 5.30**	77.54	77.92	**77.73 ± 0.27**	73.87	71.18	**72.52 ± 1.90**
Phospholipase A2	7.18	5.35	**6.26 ± 1.30**	9.70	5.53	**7.62 ± 2.95**	8.14	12.99	**10.56 ± 3.43**
Hyaluronidase	2.32	1.44	**1.88 ± 0.62**	0.90	1.09	**1.00 ± 0.13**	1.14	0.84	**0.99 ± 0.21**
MRJPs	0.33	0.40	**0.36 ± 0.05**	0.06	0.12	**0.09 ± 0.04**	0.10	0.19	**0.15 ± 0.06**
Secapins	0.01	0.01	**0.01 ± 0.00**	0.05	0.31	**0.18 ± 0.18**	0.13	0.05	**0.09 ± 0.06**
Others	17.72	12.86	**15.29 ± 3.44**	11.74	15.03	**13.39 ± 2.33**	16.62	14.76	**15.69 ± 1.32**

Relative abundances are expressed as percentages of the total venom proteome and are reported for individual analytical duplicates and as mean ± standard deviation for Sea Level (SL), Inland Valley (IV), and Andean Foothill (AF) sample groups. Major venom protein classes are shown, including melittin, phospholipase A2, hyaluronidase, major royal jelly proteins (MRJPs), Secapins, and other proteins. Values were derived from iBAQ-based absolute quantification of pooled venom samples.

Bold values indicate the mean ± standard deviation (SD) for each geographic sample group.

The overall stoichiometric profile was consistent among geographic sample groups and closely matched the canonical composition reported for honeybee venom. Melittin remained the dominant protein component in SL, IV, and AF samples, supporting the integrity and biological representativeness of the analyzed venom pools.

Despite the overall similarity in venom composition, some quantitative differences were observed for specific protein groups. Secapins were nearly undetectable in SL samples (0.01% ± 0.00%) but showed higher relative abundances in IV (0.18% ± 0.18%) and AF (0.09% ± 0.06%) samples. Phospholipase A2 also exhibited higher relative proportions in AF samples (10.56% ± 3.43%) compared with SL samples (6.26% ± 1.30%), whereas MRJPs showed the opposite trend, reaching their highest relative abundance in SL samples (0.36% ± 0.05%).

Overall, the iBAQ-based analysis revealed a conserved core venom composition dominated by melittin and phospholipase A2, together with exploratory differences in the relative abundance of minor venom components among pooled geographic sample groups. Because the analysis was based on pooled samples with technical duplicates, these quantitative differences should be interpreted as descriptive and hypothesis-generating rather than evidence of population-level geographic variation.

### 3.3 Principal components analysis (PCA)

Principal component analysis (PCA) of the normalized proteomic dataset revealed clustering of analytical duplicates according to geographic condition (Figure 1). The first two principal components explained 76.7% of the total variance (PC1 = 56.7%; PC2 = 20.0%) (Figure 1). Duplicates from each geographic zone clustered closely after median-centering normalization, demonstrating reduced technical variability and good analytical reproducibility. The separation observed along PC1 and PC2 suggests distinct protein abundance profiles among pooled venom samples collected from SL, IV, and AF locations. Nevertheless, because no independent biological replicates were available, these differences should be considered exploratory rather than evidence of population-level geographic variation.

**FIGURE 1:**
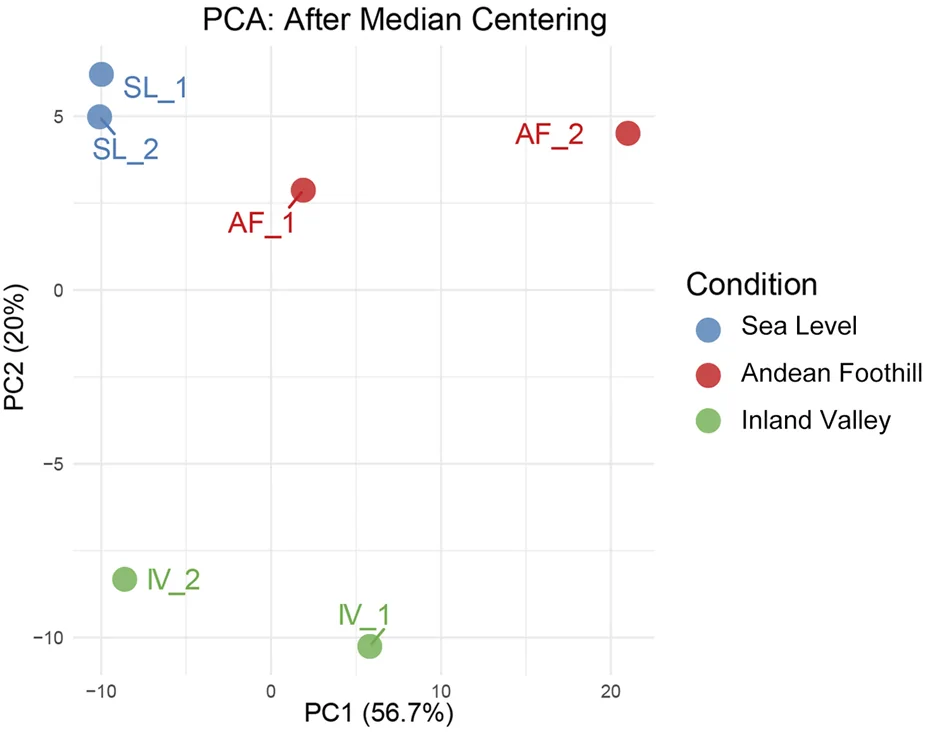
Principal component analysis (PCA) of normalized proteomic profiles of pooled *Apis mellifera* venom samples from three geographic regions of central Chile. PCA was performed using log_2_-transformed MaxLFQ intensity values after median-centering normalization. Samples represent analytical duplicates obtained from pooled venom collected at Sea Level (SL), Inland Valley (IV), and Andean Foothill (AF) sites. Principal components 1 and 2 accounted for 56.7% and 20.0% of the total variance, respectively. The clustering pattern observed along PC1 and PC2 suggests distinct protein abundance profiles among pooled venom samples collected from the three geographic zones.

### 3.4 Differential protein abundance between geographic zones

Differential abundance analysis using DEqMS (Zhu et al., 2020) identified multiple proteins showing significant abundance differences between IV and SL venom samples (Figure 2). Among the most prominent proteins, Secapin-2 exhibited markedly higher abundance in IV samples and represented the strongest positive abundance shift observed in the dataset. In contrast, several proteins involved in nutrient storage and secretion, including vitellogenin, major royal jelly proteins (MRJP1-4), and hexamerins, showed lower relative abundance in IV compared with SL samples (Supplementary Table S2). Additional differences were detected for venom-associated proteins, including venom acid phosphatase and other secreted venom components.

**FIGURE 2:**
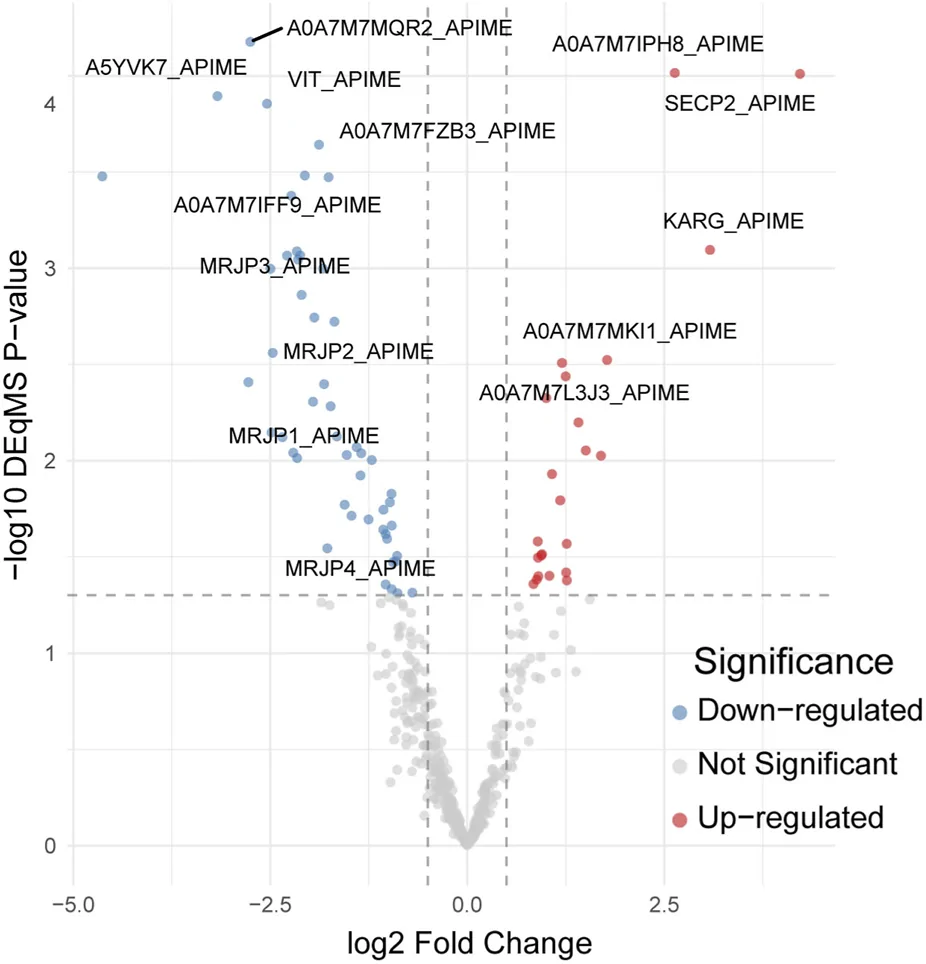
Volcano plot of differential protein abundance between Inland Valley (IV) and Sea Level (SL) venom samples. Differential abundance analysis was performed using log_2_-transformed MaxLFQ intensity values and the DEqMS statistical framework. Each point represents an identified protein. The x-axis shows the log_2_ fold change (log_2_FC) between IV and SL samples, and the y-axis represents statistical significance as −log_10_ (DEqMS p-value). Proteins exhibiting significantly higher abundance in IV and SL samples are highlighted in red and blue, respectively, whereas non-significant proteins are shown in gray. Selected proteins of interest are labeled, including Secapin-2, Vitellogenin, and major royal jelly proteins (MRJP1-4). The observed abundance differences represent exploratory findings derived from technical duplicates of pooled venom samples and should be interpreted cautiously in the absence of independent biological replicates.

The overall distribution of significant proteins across positive and negative log_2_ fold-change values suggests distinct proteomic profiles between the pooled venom samples from both locations. Functional categories recurrently represented among differentially abundant proteins included venom components, major royal jelly proteins, storage proteins, lipid-transport proteins, phospholipases, and proteins associated with redox regulation (Supplementary Tables S2–S4). Notably, the increased abundance of Secapin-2 was consistent with the iBAQ-based stoichiometric analysis, which also indicated a greater relative representation of secapins in inland geographic sample groups. Given the absence of independent biological replicates, these findings should be considered exploratory and hypothesis-generating rather than evidence of population-level geographic variation.

## 4 Discussion

The observed variation in BV proteomic profiles among geographic zones raises the hypothesis that environmental heterogeneity along the coastal–valley–Andean gradient of central Chile may contribute to differences in venom composition. This gradient encompasses marked ecological transitions, including variation in climate, vegetation structure, floral diversity, resource availability, and pollination networks, which collectively define distinct ecological contexts for honeybee colonies (Cavieres et al., 2000; Ramos-Jiliberto et al., 2010; Fernández Murillo et al., 2019; Arroyo et al., 2024). Such environmental heterogeneity could influence colony physiology through differences in resource availability, nutritional conditions, metabolic demands, and exposure to environmental stressors. Together, these factors provide a biologically plausible framework for investigating geographic variation in venom composition. However, because environmental variables were not experimentally manipulated or statistically associated with venom protein abundance, and because the present sampling design lacked independent biological replication within each geographic zone, the proteomic differences observed here cannot be directly attributed to environmental conditions. Rather, they should be interpreted as preliminary geographic patterns that generate the hypothesis that environmental context may contribute to BV proteomic variability. This possibility is potentially relevant to pharmacological research because environmentally associated variability, if confirmed, could affect the compositional consistency and biological activity of BV-derived preparations. Previous studies have reported relationships between ecological conditions, altitude, environmental stressors, and venom-related phenotypes in hymenopterans and other venomous taxa (Barkan et al., 2019; Scaccabarozzi et al., 2021), consistent with the broader concept that venom phenotypes can exhibit ecological plasticity rather than being determined exclusively by genetic factors (Gangur et al., 2017; Koludarov et al., 2023).

Building on this ecological framework, our exploratory proteomic analysis identified geographic patterns in relative protein abundance among the pooled BV samples. SL samples showed a higher relative abundance of several classical venom components, whereas IV and AF samples exhibited higher relative abundance of proteins annotated as stress-related, metabolic, or secretory. Approximately 25%–28% of the quantifiable proteins met the exploratory criteria for differential relative abundance across pairwise geographic comparisons.

Among the geographic patterns observed, SL samples exhibited a comparatively higher relative abundance of several classical venom components, including melittin, PLA_2_, and other venom-associated enzymes, resulting in a proteomic profile with a greater apparent contribution of toxin-related proteins than that observed in the IV and AF samples. These components are major determinants of the biological and pharmacological activity of BV: melittin exerts potent membrane-disruptive effects, whereas PLA_2_ and other venom enzymes contribute to inflammatory responses, tissue effects, and nociceptive signaling (Carpena et al., 2020; Shi et al., 2022). Although these abundance patterns suggest geographic variation in the relative representation of functionally important venom components, their biological significance should be interpreted cautiously given the exploratory sampling design and should be confirmed using independent biological replicates and targeted quantitative validation. The recurrent identification of venom-associated proteins among those showing the largest exploratory differences in relative abundance suggests that the SL sample may exhibit a comparatively toxin-oriented proteomic profile. However, given the absence of independent biological replicates, this pattern should not be interpreted as evidence of a distinct or more specialized venom phenotype at the population level. From an ecological perspective, if confirmed through biologically replicated studies, geographic variation in the relative abundance of these venom components could potentially reflect differences in predator pressure, colony density, interspecific interactions, or other local defensive demands. Alternatively, such variation may arise from differences in resource availability, nutritional conditions, colony physiological status, or worker age structure, all of which could influence venom production, secretion, or storage. These mechanisms were not directly evaluated in the present study and therefore represent plausible hypotheses for future investigation rather than explanations established by the current dataset (Scaccabarozzi et al., 2021).

In contrast, the AF and IV samples showed higher relative abundances of several proteins annotated as stress-associated, metabolic, or secretory, distinguishing their exploratory proteomic profiles from the profile observed in the SL sample. These included proteins involved in cellular stress responses and redox regulation, such as heat shock proteins and thioredoxin-dependent enzymes, as well as metabolic enzymes associated with glycolysis and energy homeostasis. Previous studies have documented changes in these protein classes in honeybees exposed to thermal, oxidative, and other environmental stressors, with heat shock proteins and redox-regulatory enzymes playing important roles in maintaining cellular homeostasis under physiologically challenging conditions. These findings provide a biologically plausible context for interpreting the proteomic patterns observed in the AF and IV samples. However, because environmental stressors and colony physiological status were not directly assessed in the present study, the observed abundance patterns cannot be attributed to specific environmental conditions and should instead be regarded as preliminary observations that generate hypotheses for future investigation (Shih et al., 2021; Li, 2024). Therefore, the higher relative representation of these protein classes should not be interpreted as evidence of a stress-induced or environmentally adapted venom phenotype, but rather as an exploratory pattern that warrants further investigation using independent biological replicates together with direct environmental and physiological measurements. The recurrent identification of stress-associated, metabolic, and redox-related proteins among those showing the largest exploratory differences in relative abundance raises the hypothesis that colony physiological responses may contribute to the geographic proteomic variation observed in the IV and AF samples. Inland and higher-altitude environments can differ from coastal habitats in factors such as thermal variability, water availability, and temporal patterns of floral resources, providing a plausible ecological context for this hypothesis. However, because these environmental variables and associated physiological responses were not directly measured, their contribution to the observed venom proteomic patterns cannot be established from the present dataset (Garreaud et al., 2020). The AF and IV profiles also showed higher relative abundances of MRJPs and other secreted proteins, raising the possibility that geographic variation in BV composition may extend beyond classical venom components and involve differences in the relative contribution of glandular or secretory proteins. However, because glandular activity and secretion dynamics were not directly assessed, the biological origin and significance of these differences remain uncertain and may also reflect variation in colony physiological status, worker age, or other biological and technical factors. A particularly notable observation was the higher relative abundance of MRJPs and other secreted or storage-associated proteins in AF and IV samples. MRJPs are predominantly associated with secretions of the hypopharyngeal glands of worker bees and are classically linked to nutritional, developmental, and social functions within the colony rather than to the principal toxic activities of bee venom (Altaye et al., 2019; Mattei et al., 2020). Their differential representation in the analyzed venom pools may therefore reflect variation in glandular contributions, secretion dynamics, worker physiological state, or other colony-level factors. However, these possibilities remain speculative because glandular activity and bee physiological status were not directly evaluated in the present study. Moreover, proteomic studies indicate that venom obtained by electrical stimulation may contain a broader spectrum of secreted proteins than canonical venom components alone, potentially including proteins originating from other glandular or physiological sources (Matuszewska et al., 2021; Scaccabarozzi et al., 2021). Thus, the recurrent detection and geographic variation of MRJPs in the present dataset highlight the complexity of the protein composition recovered during venom collection and raise the hypothesis that differences in bee physiology, glandular contributions, or environmental context may influence the resulting proteomic profile. Distinguishing among these possibilities will require biologically replicated sampling together with targeted analyses of venom glands and other exocrine secretions.

Taken together, these exploratory findings reveal geographic variation in the relative protein composition of the analyzed BV pools and are compatible with the broader hypothesis that venom composition may exhibit phenotypic plasticity in association with ecological and physiological factors. Rather than representing an entirely static biochemical mixture, BV may constitute a dynamic secretion whose composition varies according to the physiological state of the colony and the ecological context in which bees develop and forage. Importantly, all samples included in the present study were collected within a 2-week period during the austral summer, providing a common seasonal sampling window and thereby reducing, although not eliminating, seasonality as a potential source of variation among the analyzed pools. Consequently, the proteomic differences reported here should be interpreted as preliminary geographic patterns observed under a single seasonal context rather than as evidence of environmentally driven modulation of venom composition (Surm and Moran, 2021; Koludarov et al., 2023).

This interpretation is biologically plausible because environmental stressors are known to influence honeybee physiology, immunity, and metabolism, and accumulating proteomic evidence suggests that these physiological responses may contribute to variation in venom composition (Peiren et al., 2005; Arad et al., 2025; Al Naggar et al., 2026). More broadly, evidence from honeybees and other venomous taxa indicates that venom-related phenotypes can vary in association with environmental conditions, ecological interactions, and life-history factors, including diet, stress exposure, physiological state, and habitat characteristics. Nevertheless, direct causal evidence linking specific environmental stressors to defined alterations in the honeybee venom proteome remains limited, and the present experimental design does not allow the observed geographic differences to be attributed to specific environmental or physiological drivers. Accordingly, these findings should be regarded as hypothesis-generating and provide a foundation for future studies incorporating independent biological replicates, multi-seasonal sampling, longitudinal monitoring across contrasting environmental conditions, direct characterization of relevant environmental variables, and targeted proteomic and functional validation. Such approaches will be necessary to determine whether environmental variation contributes causally to BV proteomic variability and to establish the biological and pharmacological significance of the patterns identified here.

From a pharmacological perspective, the observed variability in venom composition has important implications. Differences in the relative abundance of bioactive peptides and enzymes may influence the therapeutic potential, efficacy, and safety profile of BV-derived products. For example, variation in melittin or phospholipase A_2_ content could affect anti-inflammatory, immunomodulatory, or cytotoxic activity, while the presence of additional non-classical proteins may modulate immunogenicity or off-target effects (Carpena et al., 2020; Sadek et al., 2024). Such geographic variation in venom composition may contribute to the variability reported across pharmacological and preclinical studies using BV from different sources. These findings emphasize the need for standardized collection protocols and detailed compositional characterization of BV used in biomedical and pharmaceutical applications, particularly when venoms are sourced from distinct geographic regions (El Mehdi et al., 2022; Oliveira Orsi et al., 2024). Despite these insights, several limitations should be considered. Each geographic condition was represented by a single pooled biological sample derived from multiple colonies, while proteomic analyses were performed using analytical duplicates generated from the same pooled material. Consequently, statistical comparisons reflect analytical reproducibility and exploratory proteomic trends rather than true biological replication, limiting population-level ecological inference. In addition, MaxLFQ-based label-free quantification provides semi-quantitative estimates that may not fully reflect absolute protein abundance. Furthermore, the absence of functional validation precludes direct assessment of how the observed proteomic differences translate into biological or pharmacological effects. Future studies should incorporate independent biological replicates, targeted quantitative proteomics, and functional assays to validate the biological significance and translational relevance of these findings.

In conclusion, this exploratory study identified preliminary differences in the relative proteomic composition of pooled *A. mellifera* venom samples collected from three geographic zones along a coastal–valley–Andean gradient in central Chile, while also revealing a substantial core of conserved venom components across samples. The observed patterns, including geographic variation in the relative abundance of classical venom proteins and proteins associated with metabolic, stress-related, and secretory functions, provide a descriptive basis for the hypothesis that environmental and physiological factors may contribute to BV proteomic variability. However, given the absence of independent biological replicates and the single seasonal sampling window, these findings should not be interpreted as evidence that environmental conditions directly modulate venom composition or as confirmation of environmentally driven venom plasticity. Rather, they represent hypothesis-generating observations that warrant validation through biologically replicated, multi-seasonal, and longitudinal studies incorporating direct characterization of environmental variables, targeted quantitative proteomics, and functional assays. If confirmed, environmentally and geographically associated variation in BV composition could have important implications for understanding venom ecology and for evaluating the compositional consistency, biological activity, and pharmacological standardization of BV-derived products.

## Data Availability

The mass spectrometry proteomics data have been deposited to the ProteomeXchange Consortium (Deutsch et al., 2023) via the PRIDE (Perez-Riverol et al., 2025) partner repository with the dataset identifier PXD079138.
