# Supplementary material for: Exploratory proteomic characterization of Apis mellifera venom across an environmental gradient in central Chile
Source: Front Pharmacol. 2026 Sep 9;17:1904856. doi: 10.3389/fphar.2026.1904856 (PMC13621212; doi:10.3389/fphar.2026.1904856)
Supplement: Supplementary file 1 [file Supplementaryfile1.docx]

Supplementary Material

**Supplementary Figure 1. Geographic location of the study sites in central Chile.** Map showing the three sampling zones in the Valparaíso Region: coastal zone (Viña del Mar, Sea Level), Inland Valley (IV, La Ligua; ~200 m above Sea Level), and Andean Foothill (AF, Putaendo; ~800 m Sea Level): These locations represent a coastal-valley-Andean altitudinal gradient used to evaluate environmental variation in the proteomic profile of *Apis mellifera* venom. The insert indicates the position of the study area within South America.

**Supplementary Figure 2.** Hierarchical clustering of differentially abundant proteins across geographic zones. Heatmap showing the normalized abundance patterns of proteins identified as differentially abundant among the studied geographic zones. Rows represent proteins and columns represent analytical duplicates from SL, AF, and IV samples. Protein abundance values were scaled by Z-score prior to clustering. Unsupervised hierarchical clustering was performed using Euclidean distance and complete linkage. Warmer colors indicate relatively higher protein abundance, whereas cooler colors indicate lower abundance. Analytical duplicates clustered according to geographic origin, supporting the reproducibility of the proteomic profiles and the geographic differences observed in the principal component analysis.

**Supplementary Figure 3. Volcano plot of differential protein abundance between Andean Foothill (AF) and Sea Level (SL) venom samples.** Differential abundance analysis was performed using log₂-transformed MaxLFQ intensity values and the DEqMS statistical framework. Each point represents an identified protein. The x-axis shows the log₂ fold change (log₂FC) between AF and SL samples, and the y-axis represents statistical significance as −log₁₀(DEqMS p-value). Proteins exhibiting significantly higher abundance in AF and SL samples are highlighted in red and blue, respectively, whereas non-significant proteins are shown in gray. Selected proteins of interest are labeled, including Secapin-2 (SECP2), major royal jelly proteins (MRJP1-4), vitellogenin, hyaluronidase, and peroxiredoxin-1. The observed abundance differences represent exploratory findings derived from technical duplicates of pooled venom samples and should be interpreted cautiously in the absence of independent biological replicates.

**Supplementary Figure 4. Volcano plot of differential protein abundance between Andean Foothill (AF) and Inland IVy (IV) venom samples.** Differential abundance analysis was performed using log₂-transformed MaxLFQ intensity values and the DEqMS statistical framework. Each point represents an identified protein. The x-axis shows the log₂ fold change (log₂FC) between AF and IV samples, and the y-axis represents statistical significance as −log₁₀(DEqMS p-value). Proteins exhibiting significantly higher abundance in AF and IV samples are highlighted in red and blue, respectively, whereas non-significant proteins are shown in gray. Selected proteins of interest are labeled, including peroxiredoxin-1, antitrypsin, secapin-1, phospholipase-related proteins, and other proteins displaying the strongest abundance differences between sample groups. The observed abundance differences represent exploratory findings derived from technical duplicates of pooled venom samples and should be interpreted cautiously in the absence of independent biological replicates.

| Geographic Zone | Total Identified Proteins | Analytical duplicate 1 n (%) | Analytical duplicate 2 n (%) |
| --- | --- | --- | --- |
| SL | 604 | 568 (94.0%) | 487 (80.6%) |
| IV | 530 | 392 (74.0%) | 511 (96.4%) |
| AF | 395 | 375 (94.9%) | 314 (79.5%) |

**Supplementary Table 1. Total number and relative proportion of identified proteins across geographic zones and analytical duplicates.** The table summarizes the total number of distinct proteins identified in BV samples collected from three geographic zones (SL, IV, and AF). Values for each analytical duplicate are presented as absolute counts (n) and percentages relative to the total number of proteins identified within the corresponding geographic zone. Percentages reflect the proportion of proteins detected in each analytical duplicate with respect to the zone-specific total proteome identified.

| **N°** | **Protein.ID** | **Gene** | **Description** | **Count** | **logFC** | **P Value** | **FDR-adjusted P value** |
| --- | --- | --- | --- | --- | --- | --- | --- |
| **1** | A0A7M7GQV1 | LOC102655185 | Uncharacterized protein LOC102655185 isoform X1 | 1 | 9,49E+14 | 3,06E+14 | 2,33E+13 |
| **2** | A0A7M7FZJ9 | LOC725926 | Collagen alpha-1(IV) chain isoform X2 | 1 | 9,41E+14 | 3,11E+14 | 2,33E+13 |
| **3** | A0A7M7MGK9 | LOC552249 | Alpha-mannosidase | 1 | -8,90E+14 | 3,12E+14 | 2,33E+13 |
| **4** | A0A7M7GAK6 | LOC408875 | Heat shock protein beta-1 isoform X2 | 1 | -1,22E+14 | 9,26E+14 | 4,19E+13 |
| **5** | A0A7M7R3G4 | 409265 | Dolichyl-diphosphooligosaccharide--protein glycosyltransferase subunit STT3A | 15 | -6,89E+14 | 1,40E+14 | 4,89E+13 |
| **6** | A0A7M7GVH6 | 409790 | Multiple coagulation factor deficiency protein 2 homolog | 168 | -7,68E+14 | 1,70E+14 | 5,32E+13 |
| **7** | A0A7M7IJ14 | LOC726352 | Transmembrane protease serine 11B-like protein | 23 | -7,74E+14 | 2,73E+14 | 6,76E+13 |
| **8** | A0A7M7G9V0 | 100576467 | 14 kDa phosphohistidine phosphatase | 13 | 3,84E+13 | 2,76E+14 | 6,79E+13 |
| **9** | A0A7M7H0Y8 | LOC412612 | Carbohydrate sulfotransferase 4 isoform X1 | 1 | 6,18E+14 | 3,26E+14 | 7,18E+13 |
| **10** | A0A7M7GTW3 | LOC102655078 | LOW QUALITY PROTEIN: zonadhesin | 9 | 5,84E+14 | 3,27E+13 | 7,18E+13 |
| **11** | A0A7M7R6A3 | LOC409945 | Aspartate--tRNA ligase, cytoplasmic | 5 | -5,24E+14 | 3,53E+14 | 7,47E+13 |
| **12** | A0A7M7R5S0 | LOC409365 | Lysosomal alpha-glucosidase | 39 | -4,40E+14 | 3,69E+14 | 7,69E+13 |
| **13** | A0A7M7FYN2 | 724489 | Ubiquitin-like protein 5 | 1 | 5,70E+14 | 3,91E+13 | 7,89E+13 |
| **14** | A0A7M7R2Y3 | 408621 | ER membrane protein complex subunit 7 | 57 | 2,86E+14 | 5,23E+14 | 8,54E+13 |
| **15** | A0A7M7H507 | LOC552141 | Beta-1,4-N-acetylgalactosaminyltransferase | 40 | 2,24E+14 | 5,19E+14 | 8,54E+13 |
| **16** | P00630 |  | Phospholipase A2 | 8768 | 2,08E+14 | 5,26E+13 | 8,54E+13 |
| **17** | A0A7M7IM62 | 552051 | Dolichyl-diphosphooligosaccharide--protein glycosyltransferase 48 kDa subunit | 7 | -3,51E+14 | 5,25E+14 | 8,54E+13 |
| **18** | A0A7M7GTC0 | 412808 | Aminopeptidase | 1 | -3,97E+14 | 5,23E+14 | 8,54E+13 |
| **19** | A0A7M7MTL0 | CUTA | Protein CutA homolog isoform X1 | 18 | -1,72E+14 | 6,08E+14 | 9,01E+13 |
| **20** | A0A7M7FZR7 | LOC725960 | Neurofilament heavy polypeptide | 49 | -3,75E+14 | 6,09E+14 | 9,01E+13 |
| **21** | A0A7M7GWL6 | LOC551763 | Hypoxia up-regulated protein 1 | 8 | 2,29E+14 | 6,90E+14 | 9,39E+13 |
| **22** | A0A7M7GZD2 | LOC408568 | Peptidyl-alpha-hydroxyglycine alpha-amidating lyase 1 isoform X4 | 1 | -1,31E+14 | 6,90E+14 | 9,39E+13 |
| **23** | A0A7M7MVS7 | LOC412513 | Uncharacterized protein LOC412513 | 131 | -1,65E+14 | 6,92E+14 | 9,39E+13 |
| **24** | A0A7M7MSF1 | LOC551913 | Protein CREG1 | 229 | -1,85E+14 | 6,90E+14 | 9,39E+13 |
| **25** | A0A7M7H421 | LOC725899 | Alpha-galactosidase | 25 | -1,93E+14 | 6,87E+13 | 9,39E+13 |
| **26** | A0A7M7H375 | 551031 | Uncharacterized protein LOC551031 | 23 | -7,55E+13 | 9,86E+14 | 9,88E+13 |
| **27** | A0A7M6UW32 | 406122 | Actin related protein 1 | 51 | -1,65E+14 | 7,49E+14 | 1,03E+14 |
| **28** | A0A7M7G1K4 | LOC724308 | trypsin | 23 | -2,35E+14 | 7,55E+14 | 1,03E+14 |
| **29** | Q06830 | PRDX1 | Peroxiredoxin-1 | 37 | -2,48E+14 | 7,13E+14 | 1,03E+14 |
| **30** | A0A7M7MN75 | LOC551880 | Uncharacterized protein LOC551880 | 4 | 1,70E+14 | 9,42E+14 | 1,08E+14 |
| **31** | P56587 |  | Tertiapin | 142 | 1,50E+14 | 8,85E+13 | 1,08E+14 |
| **32** | A0A7M6W5F9 | MRJP7 | Bee-milk protein | 115 | -1,35E+14 | 9,15E+14 | 1,08E+14 |
| **33** | A0A7M7MM69 | LOC412835 | Nucleoporin NUP35 | 76 | -1,40E+14 | 8,50E+14 | 1,08E+14 |
| **34** | A0A7M7IGY7 | 724779 | Protein artichoke | 104 | -1,53E+14 | 9,34E+14 | 1,08E+14 |
| **35** | H9KL77 | 725230 | Histone H4 | 20 | -2,21E+14 | 9,09E+14 | 1,08E+14 |
| **36** | A0A7M7G8Z8 | LOC409954 | thioredoxin-dependent peroxiredoxin | 22 | -1,21E+14 | 9,93E+13 | 1,08E+14 |
| **37** | P61626 | LYZ | Lysozyme C | 70 | -2,16E+14 | 9,69E+14 | 1,08E+14 |
| **38** | A0A7M7TFN1 | 413631 | Vanin-like protein 1 | 30 | 1,08E+14 | 1,17E+14 | 1,24E+14 |
| **39** | P00761 |  | Trypsin | 1249 | -1,35E+14 | 1,19E+14 | 1,24E+14 |
| **40** | I1VC85 |  | Secapin-2 | 1 | 4,22E+14 | 9,76E+09 | 1,25E+14 |
| **41** | A0A7M7IPH8 | LOC552229 | Carboxylic ester hydrolase | 7 | 2,63E+14 | 9,65E+09 | 1,25E+14 |
| **42** | Q868N5 | Vg | Vitellogenin | 1356 | -2,54E+14 | 1,40E+14 | 1,25E+14 |
| **43** | A0A7M7MQR2 | LOC100578030 | Antitrypsin isoform X2 | 1 | -2,75E+14 | 6,66E+08 | 1,25E+14 |
| **44** | A5YVK7 | Hex70a | Hexamerin 70a precursor | 268 | -3,17E+14 | 1,27E+14 | 1,25E+14 |
| **45** | A0A7M7H3B9 | 544670 | Elongation factor 1-alpha | 15 | -9,61E+14 | 1,49E+14 | 1,51E+14 |
| **46** | A0A7M7GZ60 | 551617 | Uncharacterized protein LOC551617 | 77 | 1,18E+14 | 1,61E+14 | 1,60E+14 |
| **47** | A0A7M7M4R3 | LOC413324 | Chitinase-like protein Idgf4 isoform X2 | 26 | -9,82E+14 | 1,65E+14 | 1,60E+14 |
| **48** | A0A8U0WQC3 | Obp14 | Odorant binding protein 14 precursor | 306 | -1,56E+13 | 1,69E+14 | 1,61E+14 |
| **49** | A0A7M7GSP5 | LOC100577777 | Cysteine-rich venom protein 6 isoform X2 | 1 | -1,76E+14 | 3,36E+14 | 1,67E+14 |
| **50** | A0A7M7FZB3 | LOC726737 | Venom acid phosphatase Acph-1 | 33 | -1,88E+14 | 2,28E+14 | 1,67E+14 |
| **51** | A0A7M7IFF9 | 409167 | Translation elongation factor 2 isoform X1 | 24 | -2,06E+14 | 3,29E+14 | 1,67E+14 |
| **52** | P01499 |  | Mast cell degranulating peptide | 180 | -4,63E+14 | 3,33E+14 | 1,67E+14 |
| **53** | A0A7M7L1T2 | LOC552572 | Eukaryotic translation initiation factor 5A | 23 | -1,06E+13 | 1,80E+14 | 1,68E+14 |
| **54** | A0A7M7G1N6 | 724386 | NPC intracellular cholesterol transporter 2 | 46 | -1,47E+14 | 1,93E+14 | 1,77E+14 |
| **55** | A0A7M6UUL3 | Obp17 | OBP17 | 29 | -1,25E+14 | 2,02E+14 | 1,81E+14 |
| **56** | A0A7M6UMR7 | Hex110 | Hexamerin 110 precursor | 5 | -2,23E+14 | 4,20E+14 | 1,88E+14 |
| **57** | A0A7M7MN89 | LOC551373 | Ectonucleoside triphosphate diphosphohydrolase 5 isoform X1 | 1 | -9,58E+14 | 2,17E+14 | 1,91E+14 |
| **58** | A0A7M7GY19 | LOC100577073 | Pigment-dispersing hormone peptides | 2 | -1,07E+14 | 2,28E+14 | 1,97E+14 |
| **59** | A0A7M7MQS4 | LOC100578030 | Antichymotrypsin-2 isoform X1 | 1 | -1,03E+14 | 2,41E+14 | 2,04E+14 |
| **60** | Q17061 | MRJP4 | Major royal jelly protein 4 | 50 | -1,02E+12 | 2,55E+14 | 2,11E+14 |
| **61** | A0A7M7R5W7 | 411943 | glutaminyl-peptide cyclotransferase | 10 | 8,96E+14 | 2,63E+14 | 2,14E+14 |
| **62** | A0A7M7LRW3 | LOC102656570 | Glucose dehydrogenase [FAD, quinone] | 171 | 1,26E+14 | 2,71E+14 | 2,16E+14 |
| **63** | A0A7M6UV61 | 411899 | Gram-negative bacteria-binding protein 1-2 precursor | 1 | -1,78E+14 | 2,85E+14 | 2,24E+14 |
| **64** | A0A7M6UFV3 | 100302106 | Uncharacterized protein LOC100302106 | 14 | 8,98E+14 | 3,19E+14 | 2,34E+14 |
| **65** | A0A7M7SRI2 | LOC552678 | Enolase | 71 | -9,06E+14 | 3,33E+14 | 2,39E+14 |
| **66** | A0A7M7L6X9 | LOC726286 | Uncharacterized protein LOC726286 | 25 | -9,42E+14 | 3,37E+14 | 2,39E+14 |
| **67** | O61367 | ARGK | Arginine kinase | 1 | 3,08E+14 | 8,03E+14 | 2,65E+14 |
| **68** | A0A7M7GAR4 | 100578168 | Fucosyltransferase | 1 | -1,83E+14 | 1,00E+14 | 2,65E+14 |
| **69** | A0A7M7IRS5 | LOC107963996 | Leucine-rich repeat-containing protein 70 | 49 | -2,12E+14 | 8,59E+14 | 2,65E+14 |
| **70** | B0LUE8 | A4 | Apolipophorin-III-like protein precursor | 107 | -2,15E+14 | 9,01E+14 | 2,65E+14 |
| **71** | P02788 | LTF | Lactotransferrin | 131 | -2,16E+14 | 8,17E+14 | 2,65E+14 |
| **72** | Q25BT7 | Hbg2 | alpha-glucosidase | 174 | -2,29E+14 | 8,59E+14 | 2,65E+14 |
| **73** | A0A7M7R759 | 409801 | Carboxylic ester hydrolase | 125 | -2,50E+14 | 1,01E+14 | 2,65E+14 |
| **74** | A0A7M7LN74 | 100578232 | Notch homolog 2 N-terminal-like protein | 5 | 1,25E+13 | 3,80E+13 | 2,66E+14 |
| **75** | A0A7M7G3D2 | 727309 | Glutaredoxin-C4 isoform X2 | 17 | 9,03E+14 | 3,98E+14 | 2,70E+14 |
| **76** | A0A7M7H331 | LOC725848 | Uncharacterized protein LOC725848 | 15 | 1,04E+14 | 3,96E+14 | 2,70E+14 |
| **77** | A0A7M7RA46 | LOC552154 | Uncharacterized protein LOC552154 | 19 | 8,82E+14 | 4,14E+14 | 2,76E+14 |
| **78** | A0A7M7R7N1 | 411799 | Polypeptide N-acetylgalactosaminyltransferase | 3 | 1,26E+14 | 4,18E+14 | 2,76E+14 |
| **79** | A0A7M7MP34 | LOC107964964 | Uncharacterized protein LOC107964964 | 21 | 8,41E+14 | 4,37E+14 | 2,81E+14 |
| **80** | A0A7M7SQT4 | LOC100578744 | Uncharacterized protein LOC100578744 | 158 | -1,03E+14 | 4,39E+13 | 2,81E+14 |
| **81** | A0A7M7R9F4 | 552600 | GILT-like protein 1 | 80 | -9,59E+14 | 4,65E+14 | 2,93E+14 |
| **82** | A0A7M7GUN4 | LOC411006 | Endoplasmic reticulum lectin 1 | 1 | -6,96E+14 | 4,84E+14 | 2,99E+14 |
| **83** | A0A7M6UVZ7 | 727472 | Peptidoglycan-recognition protein | 64 | -8,84E+14 | 4,88E+13 | 2,99E+14 |
| **84** | A0A7M7R2L6 | 408572 | Transgelin | 21 | -9,97E+14 | 5,12E+14 | 3,10E+14 |
| **85** | A0A7M7G0H4 | LOC727116 | Uncharacterized protein LOC727116 | 6 | 1,55E+14 | 5,24E+14 | 3,12E+14 |
| **86** | A0A7M7R3H9 | LOC409278 | Apolipoprotein D | 21 | -9,05E+14 | 5,29E+14 | 3,12E+14 |
| **87** | A0A7M7GB31 | 100577576 | Protein obstructor-E | 20 | 6,53E+14 | 5,73E+13 | 3,13E+14 |
| **88** | A0A7M7LKE8 | LOC724436 | phospholipase A2 | 545 | -1,10E+14 | 5,50E+14 | 3,13E+14 |
| **89** | A0A7M7R625 | 412886 | Tubulin alpha chain | 1 | -1,75E+14 | 5,62E+14 | 3,13E+14 |
| **90** | P56682 |  | Chymotrypsin inhibitor | 49 | -1,85E+14 | 5,44E+14 | 3,13E+14 |
| **91** | A0A7M7R7J6 | LOC409861 | Nucleoside diphosphate kinase | 66 | -8,13E+14 | 5,72E+13 | 3,13E+14 |
| **92** | A0A7M7R9P8 | 551250 | Microsomal triglyceride transfer protein large subunit | 16 | -8,17E+14 | 5,55E+14 | 3,13E+14 |
| **93** | A0A7M7MP24 | LOC551589 | Hexosyltransferase | 6 | 1,19E+14 | 6,05E+14 | 3,26E+14 |
| **94** | A0A7M7R7L3 | 412543 | Uncharacterized protein LOC412543 isoform X1 | 14 | -7,14E+14 | 6,17E+14 | 3,29E+14 |
| **95** | Q17060 | MRJP3 | Major royal jelly protein 3 | 374 | -2,10E+14 | 1,38E+14 | 3,42E+14 |
| **96** | A0A7M7IMI7 | 724477 | Vitamin K-dependent protein C isoform X1 | 1 | 7,25E+14 | 6,98E+14 | 3,68E+14 |
| **97** | Q1W633 | Obp21 | Odorant binding protein 21 precursor | 78 | -8,33E+14 | 7,23E+14 | 3,77E+14 |
| **98** | A0A7M7GKS8 | LOC411889 | Serine protease K12H4.7 | 86 | -8,71E+14 | 7,35E+14 | 3,79E+14 |
| **99** | A0A7M7L685 | LOC724890 | Laccase-1 | 12 | 7,17E+14 | 8,08E+14 | 3,87E+14 |
| **100** | A0A088AA06 | 552398 | Hexosyltransferase | 1 | 6,69E+14 | 7,91E+14 | 3,87E+14 |
| **101** | A0A7M7GU71 | LOC552077 | ER membrane protein complex subunit 1 | 1 | 5,55E+14 | 8,01E+14 | 3,87E+14 |
| **102** | A0A7M7TG30 | 552555 | Synaptobrevin homolog YKT6 | 2 | 1,10E+14 | 8,02E+14 | 3,87E+14 |
| **103** | A0A7M7MNC0 | LOC726444 | Ectonucleoside triphosphate diphosphohydrolase 5 | 132 | -8,70E+13 | 8,21E+14 | 3,87E+14 |
| **104** | A0A7M7L0F8 | LOC552283 | glutathione transferase | 33 | -7,16E+14 | 7,71E+14 | 3,87E+14 |
| **105** | Q8MQS8 |  | Venom serine protease 34 | 1 | -7,24E+14 | 8,20E+14 | 3,87E+14 |
| **106** | A0A7M7R743 | 409341 | Lysosomal aspartic protease | 273 | -8,57E+14 | 7,92E+14 | 3,87E+14 |
| **107** | A0A7M7GZX8 | LOC725078 | Uncharacterized protein LOC725078 isoform X1 | 1 | -6,08E+13 | 8,41E+14 | 3,92E+14 |
| **108** | A0A7M7TEW6 | 408782 | Tubulin beta chain | 3 | -5,38E+13 | 9,03E+13 | 4,13E+14 |
| **109** | A0A7M7MGP7 | LOC100576383 | alpha-1,2-Mannosidase | 1 | -7,03E+14 | 9,03E+14 | 4,13E+14 |
| **110** | O77061 | MRJP2 | Major royal jelly protein 2 | 27 | -1,69E+14 | 1,89E+14 | 4,24E+14 |
| **111** | A0A7M7SQ18 | LOC408961 | Apolipophorins | 2185 | -1,94E+14 | 1,80E+14 | 4,24E+14 |
| **112** | A0A7M7GAF4 | 100576444 | Chymotrypsin inhibitor | 246 | 1,31E+14 | 9,62E+14 | 4,31E+14 |
| **113** | A0A7M7RAL9 | 551381 | Uncharacterized protein LOC551381 | 19 | -1,03E+14 | 1,01E+14 | 4,46E+14 |
| **114** | A0A7M6UCT9 | Hsc70-4 | Heat shock protein cognate 4 | 86 | -7,61E+14 | 1,02E+14 | 4,49E+14 |
| **115** | A0A7M7LKD3 | LOC724873 | Ras-related protein Rab-7a | 5 | 9,34E+14 | 1,05E+13 | 4,55E+14 |
| **116** | A0A7M7H3H4 | 725215 | Venom acid phosphatase Acph-1 | 433 | 8,04E+14 | 1,06E+14 | 4,58E+14 |
| **117** | A0A7M6UDS2 | 409398 | Superoxide dismutase [Cu-Zn] | 76 | -7,23E+14 | 1,08E+13 | 4,60E+14 |
| **118** | A0A088AHC8 | 410122 | Glyceraldehyde-3-phosphate dehydrogenase | 29 | -6,64E+14 | 1,12E+14 | 4,72E+14 |
| **119** | A0A7M7SRZ1 | LOC113219268 | Uncharacterized protein LOC113219268 isoform X1 | 1 | 7,31E+14 | 1,14E+14 | 4,76E+14 |
| **120** | P83563 |  | Allergen Api m 6.03 / Api m 6.04 | 939 | 6,88E+14 | 1,26E+12 | 4,77E+14 |
| **121** | A0A7M7R6X2 | LOC409091 | Neurotrimin | 132 | 6,66E+14 | 1,24E+14 | 4,77E+14 |
| **122** | A0A7M7GYE7 | LOC412273 | Probable chitinase 10 isoform X2 | 1 | 6,20E+14 | 1,19E+14 | 4,77E+14 |
| **123** | A0A7M7GQQ2 | LOC412795 | Cadherin-99C isoform X2 | 1 | 5,44E+14 | 1,27E+14 | 4,77E+14 |
| **124** | A0A7M7R2N0 | LOC408603 | Glucose dehydrogenase [FAD, quinone] | 20 | 1,38E+14 | 1,25E+13 | 4,77E+14 |
| **125** | H9KQJ7 |  | Omega-conotoxin-like protein 1 | 135 | 1,13E+14 | 1,26E+14 | 4,77E+14 |
| **126** | A0A7M7R860 | 551905 | Uncharacterized protein LOC551905 | 2 | -9,48E+12 | 1,17E+14 | 4,77E+14 |
| **127** | A0A7M7R723 | LOC410894 | Chymotrypsin-1 | 14 | -1,03E+14 | 1,28E+14 | 4,77E+14 |
| **128** | A0A7M7IXJ7 | 551205 | Glucosidase II subunit alpha | 1 | -5,35E+14 | 1,24E+14 | 4,77E+14 |
| **129** | A0A7M7GL03 | LOC726966 | Angiotensin-converting enzyme | 11 | -6,29E+14 | 1,19E+14 | 4,77E+14 |
| **130** | A0A7M7GPV3 | LOC551882 | Protein dj-1beta | 48 | -6,65E+14 | 1,24E+14 | 4,77E+14 |
| **131** | A0A7M7R8E3 | 411576 | Phosphoglycerate kinase | 37 | -7,35E+14 | 1,29E+14 | 4,77E+14 |
| **132** | A0A7M7L3I9 | LOC724811 | kynurenine--oxoglutarate transaminase | 144 | -7,35E+14 | 1,28E+14 | 4,77E+14 |
| **133** | A0A7M7GC96 | LOC410733 | Glucose dehydrogenase [FAD, quinone] | 225 | -8,17E+14 | 1,29E+14 | 4,77E+14 |
| **134** | A0A7M7M5Q4 | LOC100577163 | Insulin-like growth factor-binding protein complex acid labile subunit | 15 | -1,14E+14 | 1,31E+14 | 4,79E+14 |
| **135** | A0A7M7R8E8 | 411622 | Uncharacterized protein LOC411622 | 6 | 8,72E+14 | 1,32E+14 | 4,82E+14 |
| **136** | A0A7M7R9K9 | LOC413980 | CD109 antigen | 8 | 9,26E+13 | 1,36E+14 | 4,83E+14 |
| **137** | A0A7M7LPI2 | LOC413441 | DnaJ homolog subfamily C member 3 | 111 | -7,04E+12 | 1,35E+14 | 4,83E+14 |
| **138** | Q86MV4 | PPO | Phenoloxidase subunit A3 | 152 | -7,47E+13 | 1,34E+14 | 4,83E+14 |
| **139** | A0A7M6UEA8 | Ppib | Peptidyl-prolyl cis-trans isomerase | 1484 | 6,85E+14 | 1,38E+14 | 4,87E+14 |
| **140** | B2D0J4 |  | Venom dipeptidyl peptidase 4 | 41 | -7,77E+14 | 1,42E+14 | 4,94E+14 |
| **141** | A0A7M7R6I4 | 413478 | Spermine oxidase | 53 | -6,91E+14 | 1,50E+14 | 5,15E+14 |
| **142** | Q1W647 | Obp3 | Odorant binding protein 3 isoform X1 | 9 | -9,62E+14 | 1,51E+14 | 5,16E+14 |
| **143** | A0A7M7FYF7 | LOC725287 | Sulfhydryl oxidase | 27 | 6,49E+14 | 1,55E+14 | 5,17E+14 |
| **144** | A0A7M7LPS5 | 409728 | 40S ribosomal protein S5 | 9 | 4,82E+14 | 1,61E+14 | 5,17E+14 |
| **145** | A0A7M7GXZ4 | LOC726552 | NEDD8 | 5 | 4,70E+14 | 1,59E+14 | 5,17E+14 |
| **146** | A0A7M7ML78 | LOC408953 | Peroxidase isoform X3 | 1 | -4,49E+14 | 1,58E+14 | 5,17E+14 |
| **147** | A0A7M7R866 | 552091 | Group XV phospholipase A2 isoform X1 | 26 | -5,10E+14 | 1,59E+13 | 5,17E+14 |
| **148** | A0A7M7IG95 | LOC411864 | Chaoptin | 21 | -6,08E+14 | 1,62E+14 | 5,17E+14 |
| **149** | A0A7M7GPP4 | LOC725788 | Plexin domain-containing protein 2 isoform X1 | 2 | -6,25E+14 | 1,56E+14 | 5,17E+14 |
| **150** | A0A7M7R586 | 408426 | Lysosomal thioesterase PPT2 homolog isoform X1 | 2 | -6,29E+14 | 1,60E+14 | 5,17E+14 |
| **151** | Q08169 |  | Hyaluronidase | 610 | -6,71E+14 | 1,58E+14 | 5,17E+14 |
| **152** | A0A7M7RB96 | 551968 | Aldose reductase | 35 | -5,48E+14 | 1,64E+14 | 5,22E+14 |
| **153** | A0A7M7MKK8 | LOC408874 | Uncharacterized protein LOC408874 isoform X9 | 413 | -6,72E+14 | 1,68E+14 | 5,29E+14 |
| **154** | A0A7M7FZ07 | LOC726429 | Uncharacterized protein LOC726429 | 38 | -6,79E+13 | 1,72E+14 | 5,37E+14 |
| **155** | A0A7M7R8L8 | LOC413614 | Ras-related protein Rab-14 | 6 | 5,25E+14 | 1,76E+14 | 5,37E+14 |
| **156** | A0A7M7GFE2 | LOC100577168 | Uncharacterized protein LOC100577168 | 37 | -5,52E+13 | 1,77E+14 | 5,37E+14 |
| **157** | A0A7M7GM85 | 550671 | CLIP domain-containing serine protease | 402 | -6,42E+14 | 1,74E+13 | 5,37E+14 |
| **158** | A0A7M7GAZ8 | 409406 | NTF2-related export protein | 1 | -9,03E+14 | 1,77E+14 | 5,37E+14 |
| **159** | A0A7M7H042 | LOC725552 | DNA-directed RNA polymerase subunit | 1 | -7,64E+14 | 1,80E+13 | 5,42E+14 |
| **160** | A0A7M7R7E3 | LOC551276 | Isocitrate dehydrogenase [NADP] | 8 | -4,54E+14 | 1,82E+14 | 5,45E+14 |
| **161** | A0A7M7GS92 | 100578168 | Fucosyltransferase | 1 | -4,75E+14 | 1,85E+14 | 5,50E+14 |
| **162** | A0A7M7LIH2 | 726495 | Protein D2 | 10 | 6,31E+14 | 1,91E+14 | 5,62E+14 |
| **163** | O97432 | MRJP5 | Major royal jelly protein 5 | 117 | -6,97E+14 | 1,92E+14 | 5,62E+14 |
| **164** | A0A7M7GRZ5 | 409668 | Proteasome inhibitor PI31 subunit | 1 | -5,36E+14 | 1,95E+14 | 5,67E+14 |
| **165** | A0A7M7GQB8 | LOC552636 | Dipeptidase | 1 | -8,22E+14 | 1,98E+14 | 5,73E+14 |
| **166** | A0A7M6UD74 | 412484 | Peptidoglycan-recognition protein | 1 | -2,47E+14 | 2,75E+14 | 5,87E+14 |
| **167** | O77727 | KRT15 | Keratin, type I cytoskeletal 15 | 27 | -9,24E+14 | 2,05E+14 | 5,88E+14 |
| **168** | A0A7M7R7Z9 | 551684 | Ferritin | 152 | -5,41E+13 | 2,12E+14 | 6,04E+14 |
| **169** | Q6TGR0 | Mrjp8 | Bee-milk protein | 1399 | -6,27E+14 | 2,14E+14 | 6,04E+14 |
| **170** | P62979 | RPS27A | Ubiquitin-40S ribosomal protein S27a | 1 | -7,95E+14 | 2,14E+14 | 6,04E+14 |
| **171** | A0A7M7MKI1 | LOC726101 | Uncharacterized protein LOC726101 | 27 | 1,77E+14 | 3,00E+14 | 6,05E+14 |
| **172** | A0A7M7L3J3 | LOC413785 | peptidylprolyl isomerase | 1 | 1,20E+13 | 3,11E+14 | 6,05E+14 |
| **173** | R4TRB9 | LOC113218576 | LYS1 | 475 | -6,15E+14 | 2,17E+14 | 6,07E+14 |
| **174** | A0A7M7MU23 | LOC551651 | Ferritin | 305 | -5,67E+14 | 2,20E+14 | 6,11E+14 |
| **175** | A0A7M7IS49 | 552115 | peptidylprolyl isomerase | 16 | 8,08E+14 | 2,31E+14 | 6,15E+14 |
| **176** | A0A7M7FYU4 | 726196 | Ubiquitin-fold modifier 1 | 10 | 5,05E+14 | 2,33E+14 | 6,15E+14 |
| **177** | A0A7M7KZK9 | LOC551170 | Uncharacterized protein LOC551170 isoform X3 | 1 | 4,70E+14 | 2,35E+14 | 6,15E+14 |
| **178** | A0A7M7LP09 | 410380 | Neprilysin-2 isoform X2 | 1 | 4,48E+13 | 2,34E+13 | 6,15E+14 |
| **179** | A0A7M7GRI9 | 409849 | Fasciclin-2 isoform X3 | 1 | -3,77E+14 | 2,31E+14 | 6,15E+14 |
| **180** | A0A7M7LMV8 | 413749 | Serine protease inhibitor 88Ea isoform X1 | 1 | -4,33E+14 | 2,33E+14 | 6,15E+14 |
| **181** | A0A7M6UVG4 | Cpap3-b | Cuticular protein analogous to peritrophins 3-B precursor | 148 | -5,47E+14 | 2,35E+14 | 6,15E+14 |
| **182** | A0A7M7GWC9 | 411272 | Acyl-CoA-binding protein | 7 | -7,07E+14 | 2,25E+14 | 6,15E+14 |
| **183** | A0A7M7IKU1 | LOC726698 | Serine protease snake | 73 | -7,51E+14 | 2,25E+13 | 6,15E+14 |
| **184** | A0A7M7IQZ1 | LOC107965485 | Uncharacterized protein LOC107965485 | 47 | -7,62E+14 | 2,31E+14 | 6,15E+14 |
| **185** | A0A7M7GV42 | 408851 | Cysteine proteinase CG12163 isoform X1 | 9 | 6,72E+13 | 2,39E+14 | 6,19E+14 |
| **186** | A0A7M7GB63 | 409481 | ADP-ribosylation factor | 41 | -4,87E+14 | 2,39E+14 | 6,19E+14 |
| **187** | A0A7M7IJI5 | LOC107964911 | CRISP/Allergen/PR-1 | 4 | -6,54E+14 | 2,42E+14 | 6,22E+14 |
| **188** | A0A7M7G9Y8 | 100577844 | Uncharacterized protein LOC100577844 isoform X1 | 1 | 3,71E+14 | 2,46E+14 | 6,29E+14 |
| **189** | A0A7M7FZ28 | LOC724644 | Uncharacterized protein LOC724644 | 244 | -9,03E+14 | 2,53E+13 | 6,44E+14 |
| **190** | A0A7M7SS83 | LOC552435 | alpha-1,2-Mannosidase | 43 | 4,54E+14 | 2,65E+14 | 6,68E+14 |
| **191** | A0A7M6UMJ2 | Obp13 | OBP13 | 75 | -4,96E+14 | 2,64E+14 | 6,68E+14 |
| **192** | A0A7M7GWX8 | LOC100578909 | Procollagen-lysine,2-oxoglutarate 5-dioxygenase 3 isoform X1 | 1 | -4,05E+14 | 2,69E+14 | 6,73E+14 |
| **193** | A0A7M7MQW1 | LOC552683 | Chitinase domain-containing protein 1 | 146 | -4,82E+14 | 2,72E+14 | 6,76E+14 |
| **194** | A0A7M7GBS8 | LOC726252 | Titin isoform X3 | 1 | 1,25E+14 | 3,65E+14 | 6,81E+14 |
| **195** | A0A7M7G060 | 726733 | Cysteine-rich protein 1 | 23 | 7,81E+14 | 2,86E+14 | 6,86E+14 |
| **196** | A0A7M7GZH3 | 409488 | Protein-L-isoaspartate O-methyltransferase | 1 | 3,57E+14 | 3,05E+14 | 6,86E+14 |
| **197** | A0A7M7IL97 | 551044 | Glucose dehydrogenase [FAD, quinone] isoform X2 | 1 | 3,13E+14 | 3,00E+14 | 6,86E+14 |
| **198** | A0A7M7R3I2 | LOC409298 | Formylglycine-generating enzyme | 18 | 3,80E+12 | 3,02E+14 | 6,86E+14 |
| **199** | A0A7M7IGQ7 | LOC100576326 | Phenoloxidase-activating factor 2 isoform X1 | 1 | -7,75E+13 | 2,99E+14 | 6,86E+14 |
| **200** | A0A7M7FXY2 | LOC725038 | NPC intracellular cholesterol transporter 2 homolog a | 178 | -9,27E+13 | 2,82E+14 | 6,86E+14 |
| **201** | A0A7M7R7C9 | LOC410035 | Dorsal-ventral patterning protein Sog isoform X2 | 7 | -3,26E+14 | 2,89E+14 | 6,86E+14 |
| **202** | A0A7M7L949 | LOC413012 | EH domain-containing protein 3 | 21 | -4,01E+14 | 2,86E+14 | 6,86E+14 |
| **203** | A0A7M7FZV4 | 725931 | Multiple inositol polyphosphate phosphatase 1 | 17 | -4,08E+14 | 3,00E+14 | 6,86E+14 |
| **204** | Q5BLY5 |  | Venom acid phosphatase Acph-1 | 1 | -4,25E+14 | 3,06E+14 | 6,86E+14 |
| **205** | A0A7M7FYN8 | LOC725756 | Beta-galactosidase | 451 | -4,80E+14 | 3,04E+14 | 6,86E+14 |
| **206** | A0A7M7GQ25 | LOC409517 | Digestive cysteine proteinase 1 | 278 | -4,89E+14 | 2,98E+14 | 6,86E+14 |
| **207** | A0A7M7SQD3 | LOC551435 | Protein disulfide-isomerase | 577 | -5,10E+14 | 2,97E+14 | 6,86E+14 |
| **208** | A0A7M7L6P4 | LOC551664 | Palmitoyl-protein thioesterase 1 | 310 | -5,24E+14 | 3,01E+14 | 6,86E+14 |
| **209** | A0A7M7GSN5 | 102654987 | Ras-related protein Rab-1A | 24 | -5,26E+14 | 3,00E+13 | 6,86E+14 |
| **210** | A0A7M7IHP6 | 410234 | UDP-glucuronate decarboxylase | 62 | -5,44E+14 | 3,02E+14 | 6,86E+14 |
| **211** | P02768 | ALB | Albumin | 134 | -5,58E+14 | 3,02E+14 | 6,86E+14 |
| **212** | A0A7M7LRK3 | LOC552588 | Lipase 1 isoform X3 | 1 | -6,85E+14 | 2,82E+14 | 6,86E+14 |
| **213** | Q86PH6 | Tsf1 | Transferrin | 928 | -1,82E+14 | 4,00E+14 | 6,89E+14 |
| **214** | A0A7M7RA20 | LOC408807 | IRP30 | 53 | -2,78E+14 | 3,91E+14 | 6,89E+14 |
| **215** | A0A7M7L386 | LOC724816 | Carboxypeptidase D | 1 | -3,65E+14 | 3,17E+13 | 7,07E+14 |
| **216** | A0A7M7LIA2 | 724382 | Protein obstructor-E | 12 | 3,60E+14 | 3,22E+14 | 7,13E+14 |
| **217** | A0A7M7TFR9 | 551785 | 6-phosphogluconolactonase | 30 | 4,11E+14 | 3,32E+13 | 7,25E+14 |
| **218** | A0A7M6UQF8 | Obp4 | Odorant binding protein 4 precursor | 60 | 3,58E+14 | 3,37E+14 | 7,33E+14 |
| **219** | Q6W3E3 | Mrjp6 | Bee-milk protein | 33 | -5,17E+13 | 3,40E+14 | 7,35E+14 |
| **220** | A0A7M7LM75 | LOC409826 | Proteoglycan 4 isoform X2 | 1 | 5,94E+14 | 3,45E+14 | 7,35E+14 |
| **221** | A0A7M7G8H6 | LOC726320 | Serine protease inhibitor 27A isoform X1 | 1 | -3,37E+14 | 3,42E+14 | 7,35E+14 |
| **222** | A0A7M7GXT7 | LOC409533 | Annexin | 1 | -4,44E+14 | 3,43E+14 | 7,35E+14 |
| **223** | A0A7M7R5Q5 | 412393 | Cytosolic non-specific dipeptidase | 33 | -3,91E+14 | 3,52E+14 | 7,47E+14 |
| **224** | A0A7M6UVD3 | 551975 | Peroxiredoxin-like protein | 28 | -6,48E+11 | 3,65E+14 | 7,64E+14 |
| **225** | A0A7M7H494 | 409708 | Glucosylceramidase | 1 | -2,96E+14 | 3,65E+14 | 7,64E+14 |
| **226** | Q8I6X7 | CSP3 | Antennal-specific protein 3c | 36 | -3,50E+14 | 3,75E+14 | 7,74E+14 |
| **227** | A0A7M7GY96 | LOC552007 | Pyruvate kinase | 1 | -5,89E+14 | 3,75E+14 | 7,74E+14 |
| **228** | A0A7M7GST0 | LOC410470 | Polypeptide N-acetylgalactosaminyltransferase | 1 | 5,54E+14 | 3,78E+13 | 7,77E+14 |
| **229** | A0A7M7GSG4 | 408474 | Apyrase isoform X1 | 1 | 1,00E+14 | 4,73E+14 | 7,85E+14 |
| **230** | A0A7M6UDU9 | 494523 | Glutathione peroxidase | 1 | -3,44E+14 | 3,88E+14 | 7,86E+14 |
| **231** | A0A7M7L1N9 | 726269 | Glutathione peroxidase | 278 | -4,07E+14 | 3,85E+14 | 7,86E+14 |
| **232** | A0A7M7R475 | 410121 | Thioredoxin domain-containing protein 11 | 6 | -4,81E+14 | 3,87E+14 | 7,86E+14 |
| **233** | A0A7M7GS09 | 724531 | Small ribosomal subunit protein eS28 | 5 | 4,97E+14 | 3,93E+14 | 7,90E+14 |
| **234** | A0A7M7GNL3 | LOC552697 | Zinc transporter foi isoform X1 | 6 | 4,83E+14 | 3,95E+14 | 7,90E+14 |
| **235** | A0A7M7TFI6 | 412670 | phosphoserine transaminase | 4 | 4,34E+14 | 4,14E+14 | 7,90E+14 |
| **236** | A0A7M7GL87 | LOC410975 | Uncharacterized protein LOC410975 isoform X1 | 1 | 3,24E+14 | 3,99E+14 | 7,90E+14 |
| **237** | A0A7M7RB56 | 552645 | Mesencephalic astrocyte-derived neurotrophic factor homolog | 32 | 3,14E+14 | 4,05E+14 | 7,90E+14 |
| **238** | A0A7M7M3X6 | 408666 | Uncharacterized protein LOC408666 | 1614 | -4,39E+13 | 4,13E+14 | 7,90E+14 |
| **239** | A0A7M7MN70 | LOC408618 | Furin-like protease 2 isoform X1 | 2 | -2,51E+14 | 4,01E+14 | 7,90E+14 |
| **240** | A0A7M7R9E8 | LOC550785 | Fructose-bisphosphate aldolase | 29 | -3,73E+14 | 4,03E+14 | 7,90E+14 |
| **241** | A0A7M7ILH0 | LOC410362 | MOXD1 homolog 2 | 214 | -3,86E+14 | 4,13E+14 | 7,90E+14 |
| **242** | A0A7M7R7K3 | 551273 | Lysosomal Pro-X carboxypeptidase | 312 | -4,01E+14 | 3,98E+14 | 7,90E+14 |
| **243** | A0A7M7GER9 | LOC552798 | Sodium-coupled neutral amino acid transporter 10 isoform X1 | 1 | -4,04E+14 | 4,12E+14 | 7,90E+14 |
| **244** | A0A7M7FYQ3 | LOC725387 | Uncharacterized protein DDB_G0283357 | 16 | -7,02E+14 | 4,11E+13 | 7,90E+14 |
| **245** | P13645 | KRT10 | Keratin, type I cytoskeletal 10 | 211 | -8,89E+14 | 4,04E+14 | 7,90E+14 |
| **246** | A0A087ZYX8 | LOC409143 | Venom serine protease 34 isoform X1 | 1 | -2,94E+14 | 4,18E+13 | 7,90E+14 |
| **247** | A0A7M7MQD1 | LOC410337 | Venom dipeptidyl peptidase 4 isoform X1 | 404 | -3,88E+14 | 4,17E+14 | 7,90E+14 |
| **248** | A0A7M6UX04 | 725256 | Cuticular protein 12 precursor | 4 | -1,96E+13 | 4,94E+14 | 7,90E+14 |
| **249** | O18330 | MRJP1 | Major royal jelly protein 1 | 817 | -1,73E+14 | 5,21E+14 | 8,05E+14 |
| **250** | A0A7M7MSM4 | LOC409257 | Uncharacterized protein LOC409257 isoform X1 | 1 | -3,28E+14 | 4,31E+14 | 8,09E+14 |
| **251** | A0A7M7GNU1 | LOC725883 | Guanine nucleotide-binding protein subunit gamma-1 | 25 | -3,30E+14 | 4,32E+12 | 8,09E+14 |
| **252** | A0A7M7GL48 | 413256 | Uncharacterized protein LOC413256 | 297 | 3,90E+14 | 4,34E+14 | 8,11E+14 |
| **253** | A0A7M7R8D8 | 411647 | Calumenin | 268 | 3,49E+14 | 4,40E+13 | 8,11E+14 |
| **254** | A0A7M7IHB7 | 551211 | Elongation factor 1-gamma | 10 | -3,22E+14 | 4,36E+14 | 8,11E+14 |
| **255** | A0A7M7GXX6 | 727390 | Uncharacterized protein LOC727390 isoform X2 | 1 | -3,41E+14 | 4,42E+14 | 8,11E+14 |
| **256** | A0A7M7GMA7 | 408412 | Prohibitin | 9 | -4,69E+14 | 4,41E+14 | 8,11E+14 |
| **257** | A0A7M7GPD9 | LOC551012 | Cation-independent mannose-6-phosphate receptor isoform X1 | 1 | 4,80E+14 | 4,46E+14 | 8,11E+14 |
| **258** | A0A7M7MQM2 | LOC409307 | Uncharacterized protein LOC409307 | 41 | 3,30E+14 | 4,47E+14 | 8,11E+14 |
| **259** | A0A7M6W880 | MRJP9 | Bee-milk protein | 2026 | -3,36E+14 | 4,46E+14 | 8,11E+14 |
| **260** | A0A7M7MP76 | LOC413274 | guanylate cyclase | 1 | -2,88E+14 | 4,53E+14 | 8,14E+14 |
| **261** | A0A7M7GM92 | LOC411748 | LOW QUALITY PROTEIN: protein kinase C-binding protein NELL1 | 74 | -3,26E+14 | 4,51E+14 | 8,14E+14 |
| **262** | Q1W638 | Obp16 | Odorant binding protein 16 precursor | 10 | 3,32E+14 | 4,74E+14 | 8,28E+14 |
| **263** | B2D0J5 |  | Venom carboxylesterase-6 | 2426 | 2,93E+14 | 4,70E+14 | 8,28E+14 |
| **264** | A0A7M7RAC1 | 551350 | Myotrophin | 14 | 2,57E+14 | 4,74E+14 | 8,28E+14 |
| **265** | A0A7M7IKC6 | 412394 | alpha-L-fucosidase | 1 | -2,17E+12 | 4,81E+14 | 8,28E+14 |
| **266** | P04264 | KRT1 | Keratin, type II cytoskeletal 1 | 518 | -9,73E+13 | 4,68E+14 | 8,28E+14 |
| **267** | A0A7M7L2X7 | LOC412103 | Transport and Golgi organization protein 1 isoform X4 | 1 | -2,32E+14 | 4,66E+14 | 8,28E+14 |
| **268** | A0A7M7ISX2 | LOC412198 | UDP-glucose:glycoprotein glucosyltransferase isoform X1 | 14 | -2,42E+14 | 4,82E+14 | 8,28E+14 |
| **269** | A0A7M7MYD9 | LOC113219418 | Uncharacterized protein LOC113219418 | 13 | -2,78E+14 | 4,80E+14 | 8,28E+14 |
| **270** | A0A7M7M5C7 | 410451 | Carboxypeptidase | 124 | -3,18E+14 | 4,76E+14 | 8,28E+14 |
| **271** | A0A7M7H478 | 725718 | Cofilin/actin-depolymerizing factor homolog | 76 | -3,44E+14 | 4,70E+13 | 8,28E+14 |
| **272** | A0A7M7R705 | 409201 | palmitoyl-protein hydrolase | 4 | -3,95E+14 | 4,75E+14 | 8,28E+14 |
| **273** | A0A7M7GJG6 | 100576745 | Leucine-rich repeat-containing protein 26 | 8 | -4,09E+14 | 4,78E+14 | 8,28E+14 |
| **274** | A0A7M7MR18 | 551268 | phospholipase A1 | 18 | 2,22E+14 | 4,87E+14 | 8,34E+14 |
| **275** | A0A7M7KYY1 | LOC725591 | Protein crumbs isoform X2 | 1 | 2,39E+14 | 4,91E+14 | 8,36E+14 |
| **276** | A0A7M7MR23 | LOC727193 | phospholipase A1 | 33 | -3,17E+14 | 4,94E+13 | 8,39E+14 |
| **277** | A0A7M7GY33 | 725882 | Uncharacterized protein LOC725882 | 35 | -2,54E+14 | 4,96E+13 | 8,39E+14 |
| **278** | A0A7M7R673 | 102656890 | Thioredoxin domain-containing protein 17 | 22 | 2,32E+14 | 5,04E+14 | 8,44E+14 |
| **279** | A0A7M7IGX3 | LOC410986 | Tolloid-like protein 1 isoform X5 | 1 | 2,17E+14 | 5,05E+14 | 8,44E+14 |
| **280** | A0A7M7M5A5 | 107965322 | Gamma-interferon-inducible lysosomal thiol reductase | 100 | -3,77E+14 | 5,07E+13 | 8,44E+14 |
| **281** | A0A7M7R9K2 | 552722 | Glyoxalase domain-containing protein 4 | 24 | -4,79E+14 | 5,03E+14 | 8,44E+14 |
| **282** | A0A7M7R6I1 | LOC408557 | Thioredoxin domain-containing protein 5 homolog | 5 | -3,50E+14 | 5,13E+14 | 8,51E+14 |
| **283** | A0A7M7L6C1 | LOC100577054 | Uncharacterized protein LOC100577054 isoform X1 | 2 | -1,88E+14 | 5,15E+14 | 8,51E+14 |
| **284** | A0A7M7GVQ8 | 552382 | Alpha-mannosidase | 1 | 1,90E+14 | 5,30E+14 | 8,58E+14 |
| **285** | A0A7M7GYF1 | LOC552568 | hydroxyacylglutathione hydrolase | 1 | 3,76E+14 | 5,47E+14 | 8,69E+14 |
| **286** | P85831 |  | Prohormone-4 | 28 | 2,24E+14 | 5,46E+14 | 8,69E+14 |
| **287** | A0A7M7TFG9 | 413429 | Purple acid phosphatase | 82 | 2,48E+12 | 5,45E+14 | 8,69E+14 |
| **288** | A0A7M7GUT3 | LOC503505 | Icarapin-like isoform X1 | 141 | -2,49E+12 | 5,46E+14 | 8,69E+14 |
| **289** | A0A7M7R8U4 | LOC412898 | peptidylglycine monooxygenase | 606 | -2,75E+14 | 5,45E+14 | 8,69E+14 |
| **290** | A0A7M7MM52 | LOC413627 | Uncharacterized protein LOC413627 | 96 | -2,50E+14 | 5,61E+14 | 8,85E+14 |
| **291** | A0A7M7MVC3 | LOC724421 | Fibrillin-2 | 66 | -5,06E+14 | 5,61E+14 | 8,85E+14 |
| **292** | A0A7M7GP09 | LOC102655788 | Uncharacterized protein LOC102655788 | 203 | 3,07E+14 | 5,64E+14 | 8,85E+14 |
| **293** | A0A7M7R6M6 | 409890 | Peptidyl-prolyl cis-trans isomerase | 84 | -2,84E+13 | 5,65E+14 | 8,85E+14 |
| **294** | A0A7M7R985 | 413489 | Deoxyribonuclease-2-alpha | 40 | -2,07E+13 | 5,72E+13 | 8,87E+14 |
| **295** | A0A7M7MM38 | LOC411392 | Protein Red | 22 | -3,97E+14 | 5,71E+14 | 8,87E+14 |
| **296** | A0A7M7H012 | LOC724993 | Uncharacterized protein LOC724993 | 18 | -4,24E+14 | 5,69E+14 | 8,87E+14 |
| **297** | A0A7M7MPW6 | 551793 | Uncharacterized protein LOC551793 isoform X1 | 1 | -1,96E+13 | 5,80E+14 | 8,93E+14 |
| **298** | A0A7M7MTG9 | LOC413579 | alpha-1,2-Mannosidase | 32 | -2,41E+14 | 5,79E+14 | 8,93E+14 |
| **299** | A0A7M7FZE8 | 725260 | Uncharacterized protein LOC725260 | 41 | -2,44E+14 | 5,82E+14 | 8,93E+14 |
| **300** | A0A7M7GTG7 | 552602 | Transmembrane emp24 domain-containing protein | 26 | 2,12E+13 | 5,85E+14 | 8,95E+14 |
| **301** | A0A7M7IGH2 | LOC409368 | LOW QUALITY PROTEIN: interferon-related developmental regulator 1-like | 67 | -2,12E+14 | 5,91E+14 | 8,98E+14 |
| **302** | A0A7M7GQD8 | LOC409290 | Large ribosomal subunit protein eL14 | 3 | -2,81E+14 | 5,91E+13 | 8,98E+14 |
| **303** | A0A7M7LQ38 | 551227 | Myb-like protein I isoform X2 | 1 | 3,26E+14 | 6,05E+14 | 9,00E+14 |
| **304** | A0A7M7TFL1 | LOC409911 | Protein disulfide-isomerase | 321 | 2,36E+14 | 5,98E+14 | 9,00E+14 |
| **305** | A0A7M7GQS0 | 551603 | Ras-related protein Rab-11A | 58 | 2,08E+13 | 5,97E+13 | 9,00E+14 |
| **306** | A4ZXC4 | Tpi | Triosephosphate isomerase | 69 | -1,99E+14 | 6,01E+14 | 9,00E+14 |
| **307** | A0A7M7L4E2 | LOC408826 | Dystroglycan 1 | 1 | -2,06E+14 | 6,01E+14 | 9,00E+14 |
| **308** | A0A7M7MMK3 | LOC113218958 | Superoxide dismutase [Cu-Zn] | 41 | -2,26E+14 | 6,04E+14 | 9,00E+14 |
| **309** | A0A7M7MTF8 | LOC413569 | protein-tyrosine-phosphatase | 1 | 1,91E+14 | 6,13E+14 | 9,04E+14 |
| **310** | Q5BLY4 |  | Icarapin-like | 1 | 1,76E+14 | 6,15E+14 | 9,04E+14 |
| **311** | A0A7M7GE53 | 100577527 | Uncharacterized protein LOC100577527 | 68 | -2,05E+14 | 6,18E+14 | 9,05E+14 |
| **312** | A0A7M7TF95 | 411832 | Translationally-controlled tumor protein homolog | 16 | 1,97E+14 | 6,28E+14 | 9,10E+14 |
| **313** | A0A7M7TFW2 | LOC552428 | Uncharacterized protein LOC552428 | 45 | -1,87E+14 | 6,27E+14 | 9,10E+14 |
| **314** | A0A7M7H2N8 | 409949 | Agrin | 33 | -2,70E+14 | 6,27E+14 | 9,10E+14 |
| **315** | A0A7M7GLV6 | 409165 | Calreticulin | 370 | 2,18E+14 | 6,42E+14 | 9,19E+14 |
| **316** | A0A7M7L992 | LOC100577764 | small monomeric GTPase | 4 | 1,34E+14 | 6,40E+14 | 9,19E+14 |
| **317** | A0A7M7LQ41 | LOC412045 | Dolichyl-diphosphooligosaccharide--protein glycosyltransferase subunit 2 | 113 | -1,95E+14 | 6,48E+14 | 9,19E+14 |
| **318** | A0A7M7GEI7 | LOC100578816 | Uncharacterized protein LOC100578816 | 19 | -2,02E+14 | 6,38E+14 | 9,19E+14 |
| **319** | A0A7M7R7F0 | 413977 | Calsyntenin-1 | 142 | -2,07E+14 | 6,45E+14 | 9,19E+14 |
| **320** | A0A7M7IKX6 | LOC410801 | Cathepsin L1 | 178 | -2,32E+14 | 6,44E+14 | 9,19E+14 |
| **321** | A0A7M7G7W8 | 725138 | Copper transport protein ATOX1 | 7 | -2,53E+14 | 6,50E+14 | 9,19E+14 |
| **322** | A0A087ZYQ1 | LOC411014 | Malate dehydrogenase | 17 | -2,53E+14 | 6,49E+14 | 9,19E+14 |
| **323** | Q10416 |  | Hymenoptaecin | 19 | 1,62E+14 | 6,54E+14 | 9,21E+14 |
| **324** | A0A7M7R746 | 406152 | Epoxide hydrolase | 5 | 2,38E+13 | 6,61E+14 | 9,27E+14 |
| **325** | A0A7M7GUX7 | LOC552663 | Pyridoxal kinase | 11 | -2,64E+14 | 6,62E+14 | 9,27E+14 |
| **326** | A0A7M7MMZ2 | LOC411344 | Fasciclin-1 isoform X4 | 1 | -1,41E+14 | 6,70E+14 | 9,34E+14 |
| **327** | A0A7M7GLE9 | 552191 | Endoplasmic reticulum resident protein 44 isoform X2 | 1 | 1,49E+14 | 6,78E+14 | 9,35E+14 |
| **328** | A0A7M7H0K2 | 552068 | Protein phosphatase 1L isoform X2 | 1 | 1,46E+14 | 6,78E+14 | 9,35E+14 |
| **329** | A0A7M7IRY4 | 410769 | Aminopeptidase | 1 | 2,89E+13 | 6,76E+14 | 9,35E+14 |
| **330** | A0A7M7GXL7 | LOC552223 | DnaJ homolog shv | 478 | -2,04E+14 | 6,72E+14 | 9,35E+14 |
| **331** | P35527 | KRT9 | Keratin, type I cytoskeletal 9 | 374 | -5,42E+14 | 6,98E+14 | 9,44E+14 |
| **332** | A0A7M7GBM8 | 100577847 | Uncharacterized protein LOC100577847 isoform X1 | 1 | 1,41E+14 | 6,33E+14 | 9,45E+14 |
| **333** | A0A7M7GQU7 | LOC412838 | Alpha-mannosidase | 16 | 1,32E+14 | 7,06E+14 | 9,52E+14 |
| **334** | A0A088AQF2 | 107965838 | Endoribonuclease | 34 | 1,46E+12 | 7,14E+14 | 9,55E+14 |
| **335** | A0A7M7TF36 | 410539 | Protein 5NUC | 184 | -1,66E+13 | 7,17E+14 | 9,55E+14 |
| **336** | A0A7M7MPK9 | LOC725048 | Nodal modulator 3 isoform X2 | 1 | -1,10E+14 | 7,13E+14 | 9,55E+14 |
| **337** | A0A7M7R5K0 | 409709 | Glucosylceramidase | 57 | -1,56E+14 | 7,15E+14 | 9,55E+14 |
| **338** | A0A7M7MVN9 | LOC409366 | Integral membrane protein 2 | 25 | -2,36E+14 | 7,20E+14 | 9,55E+14 |
| **339** | A0A7M7H423 | LOC552459 | Glycine-rich cell wall structural protein 1 isoform X2 | 1 | -2,80E+14 | 7,20E+14 | 9,55E+14 |
| **340** | A0A7M7LKF0 | LOC725273 | Uncharacterized protein LOC725273 isoform X1 | 11 | 8,72E+14 | 7,71E+14 | 9,55E+14 |
| **341** | A0A7M7R5K9 | LOC409160 | Dihydropteridine reductase | 5 | 1,94E+14 | 7,61E+14 | 9,55E+14 |
| **342** | A0A7M7IEU7 | LOC411903 | Dyslexia-associated protein KIAA0319 | 16 | 1,93E+14 | 7,67E+14 | 9,55E+14 |
| **343** | A0A7M7GMY7 | 408951 | 14-3-3 protein epsilon | 8 | 1,59E+14 | 7,80E+13 | 9,55E+14 |
| **344** | A0A7M7H6R7 | 102654353 | Fucosyltransferase | 95 | 1,25E+14 | 7,67E+14 | 9,55E+14 |
| **345** | A0A7M7LRS3 | 550798 | Protein ERGIC-53 | 143 | 1,21E+14 | 7,79E+14 | 9,55E+14 |
| **346** | A0A7M7M145 | 725498 | Protein mesh | 34 | 1,16E+14 | 7,85E+13 | 9,55E+14 |
| **347** | A0A7M7H280 | 408987 | Sortilin-related receptor | 43 | 1,16E+14 | 7,47E+13 | 9,55E+14 |
| **348** | A0A7M7GJM1 | LOC552228 | Renin receptor isoform X2 | 1 | 1,06E+14 | 7,34E+13 | 9,55E+14 |
| **349** | A0A7M7GSJ6 | 414035 | Lachesin isoform X2 | 1 | 2,16E+13 | 7,73E+14 | 9,55E+14 |
| **350** | A0A7M7R8W1 | 408608 | Uncharacterized protein LOC408608 | 7 | 1,74E+13 | 7,24E+14 | 9,55E+14 |
| **351** | A0A7M7TFK6 | LOC550734 | Thioredoxin-like protein 1 | 3 | 1,46E+13 | 7,79E+14 | 9,55E+14 |
| **352** | A0A7M7RBA5 | PGAM2 | Phosphoglycerate mutase | 61 | -1,01E+14 | 7,87E+14 | 9,55E+14 |
| **353** | A0A7M7H6B6 | 727510 | Lactosylceramide 4-alpha-galactosyltransferase isoform X2 | 1 | -1,09E+14 | 7,39E+14 | 9,55E+14 |
| **354** | A0A7M7R3V2 | LOC409663 | lysozyme | 121 | -1,10E+14 | 7,84E+14 | 9,55E+14 |
| **355** | A0A7M7R8D5 | 411542 | Signal recognition particle receptor subunit alpha homolog | 20 | -1,11E+14 | 7,84E+13 | 9,55E+14 |
| **356** | A0A7M7R900 | 412526 | Protein disulfide-isomerase A6 homolog | 59 | -1,12E+14 | 7,75E+14 | 9,55E+14 |
| **357** | A0A7M7R6H0 | 408851 | Cysteine proteinase CG12163 isoform X2 | 114 | -1,13E+14 | 7,86E+13 | 9,55E+14 |
| **358** | A0A7M7GPU6 | LOC411417 | Uncharacterized protein LOC411417 isoform X2 | 1 | -1,28E+14 | 7,49E+14 | 9,55E+14 |
| **359** | B8Y9E0 | C1q-VP | C1q-like venom protein | 190 | -1,31E+14 | 7,71E+14 | 9,55E+14 |
| **360** | A0A7M6URI6 | Hsc70-3 | Heat shock protein cognate 3 precursor | 768 | -1,34E+14 | 7,74E+14 | 9,55E+14 |
| **361** | A0A7M7R9Q2 | LOC552836 | Uncharacterized protein LOC552836 | 203 | -1,44E+14 | 7,70E+14 | 9,55E+14 |
| **362** | A0A7M7TFS9 | 551922 | NPC intracellular cholesterol transporter 2 homolog a | 142 | -1,51E+14 | 7,36E+14 | 9,55E+14 |
| **363** | A0A7M7R608 | 412150 | Heat shock protein 83 | 325 | -1,62E+14 | 7,42E+14 | 9,55E+14 |
| **364** | A0A7M7GQ60 | 408808 | Prosaposin isoform X1 | 62 | -1,66E+14 | 7,38E+14 | 9,55E+14 |
| **365** | P17722 |  | Defensin-1 | 15 | -1,72E+14 | 7,35E+14 | 9,55E+14 |
| **366** | A0A7M7MV57 | LOC727622 | Aspartate aminotransferase | 11 | -1,73E+14 | 7,74E+14 | 9,55E+14 |
| **367** | A0A7M6UMI0 | Trxr-1 | Thioredoxin reductase 1 isoform 2 | 1 | -2,10E+14 | 7,36E+14 | 9,55E+14 |
| **368** | A0A7M7MSB6 | LOC410369 | Uncharacterized protein LOC410369 | 13 | -2,22E+14 | 7,27E+14 | 9,55E+14 |
| **369** | A0A7M6UME9 | 406154 | Profilin | 9 | -8,98E+14 | 7,44E+14 | 9,55E+14 |
| **370** | A0A7M7GQQ6 | LOC100578311 | UMP-CMP kinase | 1 | -9,89E+14 | 7,70E+14 | 9,55E+14 |
| **371** | A0A7M7R503 | 408516 | Protein D2 isoform X3 | 26 | 8,62E+14 | 7,98E+14 | 9,66E+14 |
| **372** | A0A7M6UGK8 | Rpn1 | Dolichyl-diphosphooligosaccharide--protein glycosyltransferase subunit 1 | 115 | 9,32E+14 | 8,16E+14 | 9,75E+14 |
| **373** | A0A7M7R699 | 413167 | Melanotransferrin | 12 | 8,76E+14 | 8,19E+14 | 9,75E+14 |
| **374** | A0A7M7RBC3 | LOC552734 | Endoribonuclease | 17 | 1,50E+14 | 8,19E+14 | 9,75E+14 |
| **375** | A0A7M7IMS8 | LOC551688 | Band 7 protein AGAP004871 isoform X4 | 1 | 1,41E+13 | 8,18E+13 | 9,75E+14 |
| **376** | A0A7M7IRR6 | LOC413324 | Chitinase-like protein EN03 isoform X1 | 8 | -1,52E+14 | 8,13E+14 | 9,75E+14 |
| **377** | A0A7M7H289 | 727146 | Sphingomyelin phosphodiesterase | 1 | -7,97E+14 | 8,19E+14 | 9,75E+14 |
| **378** | A0A7M7MTB6 | 406146 | Hyaluronidase | 133 | -9,42E+14 | 8,21E+14 | 9,75E+14 |
| **379** | A0A7M7GQX8 | 408808 | Prosaposin isoform X2 | 16 | 1,31E+14 | 8,27E+14 | 9,78E+14 |
| **380** | A0A7M7R554 | 408327 | Uncharacterized protein LOC408327 | 312 | 1,01E+14 | 8,25E+14 | 9,78E+14 |
| **381** | A0A7M7GYC6 | 724900 | Uncharacterized protein LOC724900 isoform X3 | 1 | 9,84E+14 | 8,79E+14 | 9,78E+14 |
| **382** | A0A7M7R9S8 | LOC551844 | Stromal cell-derived factor 2-like protein 1 | 5 | 9,51E+14 | 8,57E+14 | 9,78E+14 |
| **383** | A0A7M7GAL3 | 409451 | Thioredoxin | 28 | 8,90E+14 | 8,99E+14 | 9,78E+14 |
| **384** | A0A7M7LRU3 | 551859 | Calmodulin | 37 | 8,47E+14 | 8,37E+14 | 9,78E+14 |
| **385** | A0A7M7R6Y1 | 409126 | Ras-related protein Rab-2 | 35 | 7,10E+14 | 8,50E+14 | 9,78E+14 |
| **386** | C1JYH6 | Hsp90 | Heat shock protein 83 | 23 | 6,77E+14 | 9,22E+14 | 9,78E+14 |
| **387** | A0A7M7L8V2 | LOC725169 | Succinate--CoA ligase [GDP-forming] subunit beta, mitochondrial | 41 | 6,47E+14 | 8,74E+14 | 9,78E+14 |
| **388** | A0A7M7FY77 | LOC724587 | Nucleotide exchange factor SIL1 | 3 | 6,05E+14 | 9,08E+14 | 9,78E+14 |
| **389** | A0A7M7INZ2 | 412523 | Out at first protein | 29 | 6,05E+14 | 8,79E+14 | 9,78E+14 |
| **390** | A0A7M7H1B6 | 551749 | LARGE xylosyl- and glucuronyltransferase 1 | 17 | 5,98E+14 | 8,67E+14 | 9,78E+14 |
| **391** | Q6J4Q1 | HEX70b | Hexamerin | 5 | 5,97E+14 | 9,14E+14 | 9,78E+14 |
| **392** | Q17058 |  | Alpha-glucosidase | 111 | 5,90E+14 | 9,11E+13 | 9,78E+14 |
| **393** | A0A7M7G858 | LOC725661 | Uncharacterized protein LOC725661 | 127 | 5,10E+14 | 9,00E+14 | 9,78E+14 |
| **394** | A0A7M7MQZ9 | LOC409474 | Laminin subunit gamma-1 | 393 | 5,06E+14 | 9,13E+14 | 9,78E+14 |
| **395** | A0A7M7GRE8 | LOC413987 | Polypeptide N-acetylgalactosaminyltransferase | 4 | 4,90E+14 | 8,85E+14 | 9,78E+14 |
| **396** | A0A7M7R7W3 | LOC551763 | Hypoxia up-regulated protein 1 | 27 | 4,87E+14 | 9,00E+14 | 9,78E+14 |
| **397** | A0A7M7GMF0 | 552756 | Cathepsin L1 | 210 | 4,69E+14 | 9,14E+14 | 9,78E+14 |
| **398** | A0A7M7IGK1 | LOC725122 | Uncharacterized protein LOC725122 isoform X2 | 33 | 4,56E+14 | 9,09E+14 | 9,78E+14 |
| **399** | A0A7M7GTZ8 | LOC102655329 | 14-3-3 protein zeta isoform X1 | 1 | 4,53E+14 | 9,41E+14 | 9,78E+14 |
| **400** | A0A7M7LQS4 | LOC552020 | Neuromodulin isoform X1 | 4 | 4,46E+14 | 9,32E+14 | 9,78E+14 |
| **401** | A0A7M7G0D7 | 727126 | Uncharacterized protein LOC727126 | 37 | 4,06E+14 | 9,40E+14 | 9,78E+14 |
| **402** | A0A7M7LSQ8 | 724341 | Endoplasmic reticulum resident protein 29 | 681 | 3,97E+14 | 9,31E+14 | 9,78E+14 |
| **403** | P01501 | MELT | Melittin | 4435 | 3,71E+14 | 9,23E+14 | 9,78E+14 |
| **404** | A0A7M7IJY0 | LOC409714 | protein-tyrosine-phosphatase | 1 | 3,46E+14 | 9,45E+14 | 9,78E+14 |
| **405** | A0A7M7MMB8 | LOC726872 | LDLR chaperone boca | 13 | 1,62E+14 | 9,59E+14 | 9,78E+14 |
| **406** | A0A7M7GQ55 | LOC411607 | 4a-hydroxytetrahydrobiopterin dehydratase | 12 | 1,07E+14 | 8,30E+13 | 9,78E+14 |
| **407** | A0A7M7R9B8 | 411765 | Rab GDP dissociation inhibitor | 8 | 1,06E+14 | 8,57E+14 | 9,78E+14 |
| **408** | A0A7M7R8L1 | 411890 | Nucleolysin TIAR | 4 | 3,65E+13 | 9,45E+14 | 9,78E+14 |
| **409** | A0A7M7RC42 | LOC552811 | Laccase-5 | 33 | 3,38E+13 | 9,34E+14 | 9,78E+14 |
| **410** | A0A7M7GQ39 | LOC408937 | SPARC | 51 | 2,64E+13 | 9,50E+14 | 9,78E+14 |
| **411** | A0A7M7MMJ3 | LOC551807 | Neurexin-4 isoform X4 | 1 | 1,78E+13 | 9,54E+14 | 9,78E+14 |
| **412** | A0A7M7R7J4 | LOC412231 | Trans-1,2-dihydrobenzene-1,2-diol dehydrogenase | 13 | 2,28E+12 | 9,52E+14 | 9,78E+14 |
| **413** | A0A7M6UBW1 | Obp18 | OBP18 | 26 | -6,54E+13 | 8,62E+14 | 9,78E+14 |
| **414** | A0A7M7R506 | LOC410734 | Glucose dehydrogenase [FAD, quinone] | 201 | -7,92E+13 | 8,61E+13 | 9,78E+14 |
| **415** | A0A7M7IT99 | LOC410890 | Protein seele | 41 | -1,08E+14 | 8,84E+14 | 9,78E+14 |
| **416** | A0A7M7L4T5 | LOC411978 | Myrosinase 1 | 8 | -1,25E+14 | 8,46E+14 | 9,78E+14 |
| **417** | P35908 | KRT2 | Keratin, type II cytoskeletal 2 epidermal | 183 | -1,74E+14 | 8,70E+14 | 9,78E+14 |
| **418** | A0A7M7GIK3 | 410663 | Selenoprotein F | 1 | -2,32E+14 | 9,57E+14 | 9,78E+14 |
| **419** | A0A7M7ISB8 | 552086 | Aldose 1-epimerase | 1 | -2,37E+14 | 9,37E+14 | 9,78E+14 |
| **420** | A0A7M7III5 | LOC408600 | Nucleobindin-2 | 1074 | -2,48E+14 | 9,57E+14 | 9,78E+14 |
| **421** | A0A7M7IVL6 | LOC409468 | A disintegrin and metalloproteinase with thrombospondin motifs 8 isoform X1 | 1 | -2,97E+14 | 9,28E+14 | 9,78E+14 |
| **422** | A0A7M7IQ52 | 551268 | phospholipase A1 | 42 | -3,13E+14 | 9,32E+14 | 9,78E+14 |
| **423** | A0A7M7IL03 | LOC102656186 | Trypsin-5 isoform X1 | 53 | -3,44E+14 | 9,32E+14 | 9,78E+14 |
| **424** | A0A7M7GTH3 | LOC411083 | Sodium/potassium-transporting ATPase subunit alpha | 1 | -3,47E+14 | 9,59E+14 | 9,78E+14 |
| **425** | A0A7M7GLY9 | 551780 | Uncharacterized protein CG3556 isoform X1 | 1 | -3,65E+14 | 9,17E+14 | 9,78E+14 |
| **426** | A0A7M7GX77 | 410530 | Alkaline phosphatase | 23 | -3,71E+14 | 9,24E+14 | 9,78E+14 |
| **427** | A0A7M7TF41 | 409999 | Lipase | 1 | -3,81E+14 | 8,98E+14 | 9,78E+14 |
| **428** | A0A7M7FZ02 | LOC724703 | Modular serine protease | 320 | -3,92E+14 | 9,33E+13 | 9,78E+14 |
| **429** | A0A7M7TFY7 | 552747 | Glucosidase 2 subunit beta | 141 | -4,53E+14 | 9,19E+14 | 9,78E+14 |
| **430** | A0A7M7G166 | 725813 | Uncharacterized protein LOC725813 | 44 | -5,13E+14 | 8,91E+14 | 9,78E+14 |
| **431** | A0A7M7TFR2 | LOC550884 | N-sulphoglucosamine sulphohydrolase | 133 | -5,19E+14 | 9,07E+14 | 9,78E+14 |
| **432** | A0A7M7R370 | LOC409026 | Transmembrane emp24 domain-containing protein bai | 13 | -5,26E+14 | 8,81E+14 | 9,78E+14 |
| **433** | A0A7M7MM50 | LOC409814 | Beta-glucuronidase | 106 | -5,84E+14 | 9,03E+14 | 9,78E+14 |
| **434** | A0A7M7GPB8 | LOC726421 | Membrane metallo-endopeptidase-like 1 isoform X2 | 1 | -6,46E+14 | 8,40E+14 | 9,78E+14 |
| **435** | A0A7M7GA54 | 100576174 | Uncharacterized protein LOC100576174 | 11 | -6,55E+14 | 9,14E+14 | 9,78E+14 |
| **436** | A0A7M7IHQ8 | 107965279 | phospholipase A1 | 112 | -6,93E+14 | 8,82E+14 | 9,78E+14 |
| **437** | A0A7M7GNJ2 | LOC552018 | tRNA (guanine(26)-N(2))-dimethyltransferase | 26 | -7,41E+14 | 8,62E+14 | 9,78E+14 |
| **438** | A0A7M7H184 | LOC412536 | Leukocyte elastase inhibitor | 23 | -7,64E+14 | 8,57E+14 | 9,78E+14 |
| **439** | A0A7M7FZE7 | LOC725041 | Leucine-rich repeat-containing protein 15 | 92 | -7,67E+14 | 8,54E+14 | 9,78E+14 |
| **440** | A0A7M7MNA5 | LOC409281 | Protein sel-1 homolog 1 | 43 | -8,62E+14 | 8,35E+14 | 9,78E+14 |
| **441** | A0A7M7MP33 | LOC413792 | Nucleoprotein TPR isoform X1 | 1 | 2,45E+14 | 9,69E+14 | 9,79E+14 |
| **442** | A0A7M7TFJ8 | LOC408280 | Uncharacterized protein LOC408280 | 8 | 2,28E+14 | 9,70E+14 | 9,79E+14 |
| **443** | A0A7M7G005 | 726353 | Uncharacterized protein LOC726353 isoform X1 | 1 | 1,12E+14 | 9,71E+14 | 9,79E+14 |
| **444** | Q9U8X6 | LOC406081 | Glucose oxidase | 96 | -2,38E+13 | 9,68E+14 | 9,79E+14 |
| **445** | A0A7M7GQ31 | LOC726818 | Beta-hexosaminidase | 37 | -1,31E+14 | 9,71E+14 | 9,79E+14 |
| **446** | A0A7M7GNS0 | 409004 | Ras-related protein Rab6 isoform X1 | 1 | 1,00E+14 | 9,77E+14 | 9,83E+14 |
| **447** | A0A7M7LT84 | 100578782 | Complex III subunit 9 | 29 | -1,02E+14 | 9,79E+14 | 9,83E+14 |
| **448** | A0A7M7SPQ2 | 100577408 | Leukocyte elastase inhibitor | 57 | 2,24E+14 | 9,95E+14 | 9,95E+14 |

**Supplementary Table 2. Differentially abundant proteins between IV and SL.**
Results of differential abundance analysis performed using DEqMS comparing protein abundance profiles between the IV and SL geographic zones. The table includes UniProt accession numbers, gene names, protein descriptions, peptide counts, log fold change (logFC), raw *P* values, and false discovery rate (FDR)-adjusted *P* values. Proteins are ranked according to FDR-adjusted *P* value in ascending order.

| **N°** | **Protein.ID** | **Gene** | **Description** | **Count** | **logFC** | **P Value** | **FDR-adjusted P value** |
| --- | --- | --- | --- | --- | --- | --- | --- |
| **1** | A0A7M7MP34 | LOC107964964 | Uncharacterized protein LOC107964964 | 21 | 4,10E+14 | 9,28E+14 | 9,60E+12 |
| **2** | A0A7M6UBW1 | Obp18 | OBP18 | 26 | 4,06E+14 | 9,30E+14 | 9,60E+12 |
| **3** | P01501 | MELT | Melittin | 4435 | -3,32E+14 | 9,31E+14 | 9,60E+12 |
| **4** | A0A7M7R6I4 | 413478 | Spermine oxidase | 53 | -1,65E+13 | 1,26E+14 | 1,03E+13 |
| **5** | A0A7M7GVH6 | 409790 | Multiple coagulation factor deficiency protein 2 homolog | 168 | -1,56E+14 | 1,28E+14 | 1,03E+13 |
| **6** | A0A7M7GM85 | 550671 | CLIP domain-containing serine protease | 402 | -1,28E+14 | 1,49E+14 | 1,07E+13 |
| **7** | A0A7M7R5Q5 | 412393 | Cytosolic non-specific dipeptidase | 33 | -1,11E+13 | 1,90E+14 | 1,22E+13 |
| **8** | A0A7M7R7J6 | LOC409861 | Nucleoside diphosphate kinase | 66 | -1,06E+14 | 1,83E+14 | 1,22E+13 |
| **9** | A0A7M7G8Z8 | LOC409954 | thioredoxin-dependent peroxiredoxin | 22 | -1,30E+14 | 1,91E+14 | 1,22E+13 |
| **10** | A0A7M7GUN4 | LOC411006 | Endoplasmic reticulum lectin 1 | 1 | -8,64E+14 | 1,89E+14 | 1,22E+13 |
| **11** | A0A7M7IHQ8 | 107965279 | phospholipase A1 | 112 | -1,23E+13 | 2,11E+14 | 1,26E+13 |
| **12** | A0A7M7GB63 | 409481 | ADP-ribosylation factor | 41 | -1,05E+14 | 2,12E+14 | 1,26E+13 |
| **13** | A0A7M7G166 | 725813 | Uncharacterized protein LOC725813 | 44 | -9,93E+14 | 2,08E+14 | 1,26E+13 |
| **14** | A0A7M7R7C9 | LOC410035 | Dorsal-ventral patterning protein Sog isoform X2 | 7 | -6,39E+13 | 5,19E+14 | 1,98E+13 |
| **15** | A0A7M7SQT4 | LOC100578744 | Uncharacterized protein LOC100578744 | 158 | -7,56E+14 | 1,24E+14 | 3,14E+13 |
| **16** | A0A7M7GB31 | 100577576 | Protein obstructor-E | 20 | -4,65E+14 | 1,58E+12 | 3,60E+13 |
| **17** | A0A088AHC8 | 410122 | Glyceraldehyde-3-phosphate dehydrogenase | 29 | -7,06E+14 | 1,61E+14 | 3,60E+13 |
| **18** | A0A7M7RAL9 | 551381 | Uncharacterized protein LOC551381 | 19 | -8,66E+14 | 1,59E+14 | 3,60E+13 |
| **19** | A0A7M7R9B8 | 411765 | Rab GDP dissociation inhibitor | 8 | -8,78E+14 | 1,60E+14 | 3,60E+13 |
| **20** | A0A7M7FYU4 | 726196 | Ubiquitin-fold modifier 1 | 10 | -5,22E+14 | 2,19E+14 | 4,20E+13 |
| **21** | A0A7M7R8E3 | 411576 | Phosphoglycerate kinase | 37 | -6,49E+14 | 2,60E+14 | 4,62E+13 |
| **22** | A0A7M7MVS7 | LOC412513 | Uncharacterized protein LOC412513 | 131 | -4,52E+14 | 2,89E+14 | 4,78E+13 |
| **23** | A0A7M7R506 | LOC410734 | Glucose dehydrogenase [FAD, quinone] | 201 | -4,49E+13 | 3,31E+14 | 5,12E+13 |
| **24** | A0A7M7GQ60 | 408808 | Prosaposin isoform X1 | 62 | -4,94E+14 | 3,30E+13 | 5,12E+13 |
| **25** | A4ZXC4 | Tpi | Triosephosphate isomerase | 69 | -3,67E+14 | 3,42E+14 | 5,20E+13 |
| **26** | A0A7M7GL03 | LOC726966 | Angiotensin-converting enzyme | 11 | -4,49E+14 | 3,43E+14 | 5,20E+13 |
| **27** | A0A7M7R8U4 | LOC412898 | peptidylglycine monooxygenase | 606 | -3,86E+12 | 4,00E+14 | 5,65E+13 |
| **28** | A0A7M7IGK1 | LOC725122 | Uncharacterized protein LOC725122 isoform X2 | 33 | -3,98E+14 | 4,22E+14 | 5,80E+13 |
| **29** | B8Y9E0 | C1q-VP | C1q-like venom protein | 190 | -3,12E+14 | 4,92E+14 | 6,37E+13 |
| **30** | A0A7M7IPH8 | LOC552229 | Carboxylic ester hydrolase | 7 | 1,45E+14 | 6,10E+14 | 8,22E+13 |
| **31** | A0A7M7LP09 | 410380 | Neprilysin-2 isoform X2 | 1 | -1,45E+13 | 7,66E+14 | 8,22E+13 |
| **32** | A0A7M7GJM1 | LOC552228 | Renin receptor isoform X2 | 1 | -1,01E+14 | 6,99E+14 | 8,22E+13 |
| **33** | A0A7M7MR18 | 551268 | phospholipase A1 | 18 | -1,02E+14 | 7,12E+14 | 8,22E+13 |
| **34** | A0A7M7H494 | 409708 | Glucosylceramidase | 1 | -1,04E+14 | 6,94E+14 | 8,22E+13 |
| **35** | A0A7M7MN89 | LOC551373 | Ectonucleoside triphosphate diphosphohydrolase 5 isoform X1 | 1 | -1,20E+14 | 6,63E+14 | 8,22E+13 |
| **36** | A0A7M7FZB3 | LOC726737 | Venom acid phosphatase Acph-1 | 33 | -1,22E+14 | 5,08E+14 | 8,22E+13 |
| **37** | A0A7M7RAC1 | 551350 | Myotrophin | 14 | -1,24E+14 | 4,59E+14 | 8,22E+13 |
| **38** | O77061 | MRJP2 | Major royal jelly protein 2 | 27 | -1,35E+14 | 7,59E+14 | 8,22E+13 |
| **39** | A0A7M7GKS8 | LOC411889 | Serine protease K12H4.7 | 86 | -1,44E+14 | 7,66E+14 | 8,22E+13 |
| **40** | A0A7M7LPI2 | LOC413441 | DnaJ homolog subfamily C member 3 | 111 | -1,49E+14 | 5,94E+14 | 8,22E+13 |
| **41** | A0A7M7IRS5 | LOC107963996 | Leucine-rich repeat-containing protein 70 | 49 | -1,52E+14 | 7,73E+14 | 8,22E+13 |
| **42** | A0A7M7SQ18 | LOC408961 | Apolipophorins | 2185 | -1,57E+14 | 6,88E+14 | 8,22E+13 |
| **43** | A0A7M7GQB8 | LOC552636 | Dipeptidase | 1 | -1,64E+14 | 7,30E+14 | 8,22E+13 |
| **44** | A0A7M7RC42 | LOC552811 | Laccase-5 | 33 | -1,64E+14 | 7,09E+14 | 8,22E+13 |
| **45** | Q17060 | MRJP3 | Major royal jelly protein 3 | 374 | -1,67E+14 | 6,10E+14 | 8,22E+13 |
| **46** | A0A7M7RA20 | LOC408807 | IRP30 | 53 | -2,08E+14 | 6,51E+13 | 8,22E+13 |
| **47** | A0A7M7R5K9 | LOC409160 | Dihydropteridine reductase | 5 | -2,89E+14 | 9,69E+14 | 9,81E+13 |
| **48** | Q868N5 | Vg | Vitellogenin | 1356 | -1,35E+14 | 1,12E+14 | 1,00E+14 |
| **49** | A0A7M7G1N6 | 724386 | NPC intracellular cholesterol transporter 2 | 46 | -1,32E+13 | 1,19E+14 | 1,02E+14 |
| **50** | A0A7M6UQF8 | Obp4 | Odorant binding protein 4 precursor | 60 | -1,08E+14 | 1,18E+13 | 1,02E+14 |
| **51** | A0A088A0N5 | 724633 | Histone H2B | 1 | -1,88E+14 | 1,22E+13 | 1,02E+14 |
| **52** | A0A7M7R9F4 | 552600 | GILT-like protein 1 | 80 | -1,26E+13 | 1,32E+14 | 1,05E+14 |
| **53** | Q17061 | MRJP4 | Major royal jelly protein 4 | 50 | -1,14E+14 | 1,44E+14 | 1,07E+14 |
| **54** | A5YVK7 | Hex70a | Hexamerin 70a precursor | 268 | -1,59E+14 | 1,41E+14 | 1,07E+14 |
| **55** | A0A7M7R759 | 409801 | Carboxylic ester hydrolase | 125 | -1,63E+14 | 1,45E+14 | 1,07E+14 |
| **56** | H9KL77 | 725230 | Histone H4 | 20 | -2,03E+14 | 1,40E+14 | 1,07E+14 |
| **57** | A0A7M7MNC0 | LOC726444 | Ectonucleoside triphosphate diphosphohydrolase 5 | 132 | -1,30E+12 | 1,60E+14 | 1,13E+14 |
| **58** | A0A7M7H042 | LOC725552 | DNA-directed RNA polymerase subunit | 1 | -1,24E+13 | 1,71E+14 | 1,17E+14 |
| **59** | A0A7M7R866 | 552091 | Group XV phospholipase A2 isoform X1 | 26 | -9,51E+14 | 1,70E+14 | 1,17E+14 |
| **60** | A0A7M6W5F9 | MRJP7 | Bee-milk protein | 115 | -1,16E+14 | 1,97E+14 | 1,22E+14 |
| **61** | A0A7M7R9E8 | LOC550785 | Fructose-bisphosphate aldolase | 29 | -1,46E+14 | 1,95E+14 | 1,22E+14 |
| **62** | A0A7M6UDU9 | 494523 | Glutathione peroxidase | 1 | -1,01E+14 | 2,28E+14 | 1,33E+14 |
| **63** | A0A7M6UDS2 | 409398 | Superoxide dismutase [Cu-Zn] | 76 | -1,08E+14 | 2,41E+14 | 1,33E+14 |
| **64** | A0A7M7LMV8 | 413749 | Serine protease inhibitor 88Ea isoform X1 | 1 | -1,11E+14 | 2,46E+14 | 1,33E+14 |
| **65** | A0A7M7TFS9 | 551922 | NPC intracellular cholesterol transporter 2 homolog a | 142 | -1,14E+14 | 2,50E+14 | 1,33E+14 |
| **66** | P02788 | LTF | Lactotransferrin | 131 | -1,22E+14 | 2,50E+14 | 1,33E+14 |
| **67** | O18330 | MRJP1 | Major royal jelly protein 1 | 817 | -1,29E+14 | 2,47E+14 | 1,33E+14 |
| **68** | A0A7M7LKF0 | LOC725273 | Uncharacterized protein LOC725273 isoform X1 | 11 | -7,70E+14 | 2,36E+14 | 1,33E+14 |
| **69** | A0A7M7L386 | LOC724816 | Carboxypeptidase D | 1 | -9,19E+14 | 2,35E+14 | 1,33E+14 |
| **70** | Q86PH6 | Tsf1 | Transferrin | 928 | -1,28E+14 | 2,67E+14 | 1,40E+14 |
| **71** | A0A7M7L3J3 | LOC413785 | peptidylprolyl isomerase | 1 | 8,09E+14 | 2,72E+14 | 1,41E+14 |
| **72** | A0A7M7IQZ1 | LOC107965485 | Uncharacterized protein LOC107965485 | 47 | -1,52E+14 | 2,80E+14 | 1,43E+14 |
| **73** | A0A7M7IGY7 | 724779 | Protein artichoke | 104 | -1,22E+14 | 2,89E+14 | 1,46E+14 |
| **74** | A0A7M7GQ55 | LOC411607 | 4a-hydroxytetrahydrobiopterin dehydratase | 12 | -1,01E+14 | 2,94E+14 | 1,47E+14 |
| **75** | A0A7M7IL03 | LOC102656186 | Trypsin-5 isoform X1 | 53 | -9,67E+12 | 3,26E+14 | 1,60E+14 |
| **76** | Q06830 | PRDX1 | Peroxiredoxin-1 | 37 | 1,79E+14 | 3,38E+14 | 1,63E+14 |
| **77** | Q08169 |  | Hyaluronidase | 610 | -1,08E+14 | 3,35E+14 | 1,63E+14 |
| **78** | A0A7M7III5 | LOC408600 | Nucleobindin-2 | 1074 | -1,09E+14 | 3,48E+14 | 1,65E+14 |
| **79** | A0A7M7H331 | LOC725848 | Uncharacterized protein LOC725848 | 15 | 8,49E+14 | 4,02E+14 | 1,78E+14 |
| **80** | A0A7M7R9Q2 | LOC552836 | Uncharacterized protein LOC552836 | 203 | -1,13E+14 | 3,94E+14 | 1,78E+14 |
| **81** | A0A7M7R6I1 | LOC408557 | Thioredoxin domain-containing protein 5 homolog | 5 | -1,24E+14 | 3,91E+14 | 1,78E+14 |
| **82** | A0A7M7R8E8 | 411622 | Uncharacterized protein LOC411622 | 6 | -1,48E+14 | 3,84E+14 | 1,78E+14 |
| **83** | A0A7M7R7W3 | LOC551763 | Hypoxia up-regulated protein 1 | 27 | -8,85E+14 | 3,98E+14 | 1,78E+14 |
| **84** | A0A7M6UCT9 | Hsc70-4 | Heat shock protein cognate 4 | 86 | -9,99E+14 | 3,95E+14 | 1,78E+14 |
| **85** | A0A7M7GA54 | 100576174 | Uncharacterized protein LOC100576174 | 11 | 1,62E+14 | 4,28E+14 | 1,81E+14 |
| **86** | Q86MV4 | PPO | Phenoloxidase subunit A3 | 152 | -1,06E+14 | 4,36E+14 | 1,81E+14 |
| **87** | A0A7M7R6M6 | 409890 | Peptidyl-prolyl cis-trans isomerase | 84 | -1,11E+14 | 4,13E+14 | 1,81E+14 |
| **88** | H9K244 | 725272 | Histone H3 | 6 | -1,24E+14 | 4,34E+14 | 1,81E+14 |
| **89** | A0A7M7GZE6 | 408388 | Tubulin alpha chain | 1 | -1,40E+14 | 4,34E+14 | 1,81E+14 |
| **90** | A0A7M7GPV3 | LOC551882 | Protein dj-1beta | 48 | -9,19E+14 | 4,22E+14 | 1,81E+14 |
| **91** | A0A7M7GPP4 | LOC725788 | Plexin domain-containing protein 2 isoform X1 | 2 | -9,36E+14 | 4,39E+14 | 1,81E+14 |
| **92** | A0A7M7G0H4 | LOC727116 | Uncharacterized protein LOC727116 | 6 | 1,59E+14 | 4,79E+14 | 1,86E+14 |
| **93** | A0A7M7R503 | 408516 | Protein D2 isoform X3 | 26 | -7,36E+13 | 4,75E+14 | 1,86E+14 |
| **94** | A0A7M7L3I9 | LOC724811 | kynurenine--oxoglutarate transaminase | 144 | -1,01E+14 | 4,57E+14 | 1,86E+14 |
| **95** | A0A7M7IL97 | 551044 | Glucose dehydrogenase [FAD, quinone] isoform X2 | 1 | -6,48E+14 | 4,61E+14 | 1,86E+14 |
| **96** | A0A7M7R673 | 102656890 | Thioredoxin domain-containing protein 17 | 22 | -7,49E+14 | 4,79E+14 | 1,86E+14 |
| **97** | A0A7M7SPQ2 | 100577408 | Leukocyte elastase inhibitor | 57 | -8,54E+14 | 4,79E+14 | 1,86E+14 |
| **98** | A0A7M6URI6 | Hsc70-3 | Heat shock protein cognate 3 precursor | 768 | -1,02E+13 | 4,86E+14 | 1,87E+14 |
| **99** | A0A7M7GQQ6 | LOC100578311 | UMP-CMP kinase | 1 | -7,12E+14 | 5,42E+13 | 2,04E+14 |
| **100** | A0A7M7MP76 | LOC413274 | guanylate cyclase | 1 | -7,79E+13 | 5,94E+14 | 2,16E+14 |
| **101** | A0A7M7GVQ8 | 552382 | Alpha-mannosidase | 1 | -6,21E+14 | 5,84E+14 | 2,16E+14 |
| **102** | A0A7M7IGH2 | LOC409368 | LOW QUALITY PROTEIN: interferon-related developmental regulator 1-like | 67 | -8,08E+14 | 5,93E+14 | 2,16E+14 |
| **103** | A0A7M7FZE7 | LOC725041 | Leucine-rich repeat-containing protein 15 | 92 | -8,64E+14 | 5,81E+14 | 2,16E+14 |
| **104** | A0A7M7IVL6 | LOC409468 | A disintegrin and metalloproteinase with thrombospondin motifs 8 isoform X1 | 1 | -6,65E+13 | 6,46E+14 | 2,33E+14 |
| **105** | A0A7M7RBA5 | PGAM2 | Phosphoglycerate mutase | 61 | -7,39E+14 | 6,79E+14 | 2,38E+14 |
| **106** | A0A7M7SRI2 | LOC552678 | Enolase | 71 | -7,55E+14 | 6,74E+14 | 2,38E+14 |
| **107** | A0A087ZYQ1 | LOC411014 | Malate dehydrogenase | 17 | -9,10E+14 | 6,69E+14 | 2,38E+14 |
| **108** | A0A7M7MQD1 | LOC410337 | Venom dipeptidyl peptidase 4 isoform X1 | 404 | -9,31E+14 | 6,84E+14 | 2,38E+14 |
| **109** | A0A7M7GUT3 | LOC503505 | Icarapin-like isoform X1 | 141 | -8,07E+13 | 6,91E+14 | 2,39E+14 |
| **110** | I1VC85 |  | Secapin-2 | 1 | 4,47E+14 | 5,97E+09 | 2,41E+14 |
| **111** | A0A7M7H478 | 725718 | Cofilin/actin-depolymerizing factor homolog | 76 | -9,21E+14 | 7,07E+14 | 2,42E+14 |
| **112** | P01499 |  | Mast cell degranulating peptide | 180 | -1,78E+14 | 7,21E+14 | 2,43E+14 |
| **113** | A0A7M7IKC6 | 412394 | alpha-L-fucosidase | 1 | -5,92E+14 | 7,19E+14 | 2,43E+14 |
| **114** | A0A7M7GE53 | 100577527 | Uncharacterized protein LOC100577527 | 68 | -7,92E+14 | 7,29E+14 | 2,43E+14 |
| **115** | A0A7M7IXJ7 | 551205 | Glucosidase II subunit alpha | 1 | -6,34E+14 | 7,46E+14 | 2,46E+14 |
| **116** | A0A7M7GS92 | 100578168 | Fucosyltransferase | 1 | -6,63E+14 | 7,47E+14 | 2,46E+14 |
| **117** | A0A7M7M145 | 725498 | Protein mesh | 34 | -1,00E+14 | 7,67E+14 | 2,49E+14 |
| **118** | A0A7M6UVG4 | Cpap3-b | Cuticular protein analogous to peritrophins 3-B precursor | 148 | -8,49E+14 | 7,74E+14 | 2,49E+14 |
| **119** | A0A7M7GQ25 | LOC409517 | Digestive cysteine proteinase 1 | 278 | -8,73E+14 | 7,76E+14 | 2,49E+14 |
| **120** | A0A7M7R900 | 412526 | Protein disulfide-isomerase A6 homolog | 59 | -7,43E+13 | 7,88E+13 | 2,51E+14 |
| **121** | A0A7M7MM52 | LOC413627 | Uncharacterized protein LOC413627 | 96 | -8,06E+14 | 8,03E+14 | 2,53E+14 |
| **122** | P83563 |  | Allergen Api m 6.03 / Api m 6.04 | 939 | 7,94E+14 | 8,25E+14 | 2,54E+14 |
| **123** | A0A8U0WQC3 | Obp14 | Odorant binding protein 14 precursor | 306 | -1,05E+14 | 8,51E+14 | 2,54E+14 |
| **124** | A0A7M7R2N0 | LOC408603 | Glucose dehydrogenase [FAD, quinone] | 20 | -1,59E+14 | 8,25E+13 | 2,54E+14 |
| **125** | A0A7M7TEW6 | 408782 | Tubulin beta chain | 3 | -6,76E+14 | 8,33E+14 | 2,54E+14 |
| **126** | A0A7M7R6Y1 | 409126 | Ras-related protein Rab-2 | 35 | -6,95E+14 | 8,61E+14 | 2,54E+14 |
| **127** | A0A7M7FYN8 | LOC725756 | Beta-galactosidase | 451 | -8,40E+14 | 8,66E+14 | 2,54E+14 |
| **128** | A0A7M7LSQ8 | 724341 | Endoplasmic reticulum resident protein 29 | 681 | -8,43E+14 | 8,59E+14 | 2,54E+14 |
| **129** | A0A7M7MKK8 | LOC408874 | Uncharacterized protein LOC408874 isoform X9 | 413 | -8,59E+14 | 8,57E+14 | 2,54E+14 |
| **130** | A0A7M7SQD3 | LOC551435 | Protein disulfide-isomerase | 577 | -8,84E+14 | 8,50E+14 | 2,54E+14 |
| **131** | A0A7M7GL48 | 413256 | Uncharacterized protein LOC413256 | 297 | -9,24E+14 | 8,15E+14 | 2,54E+14 |
| **132** | P62979 | RPS27A | Ubiquitin-40S ribosomal protein S27a | 1 | -1,14E+14 | 8,80E+14 | 2,54E+14 |
| **133** | A0A7M7GPU6 | LOC411417 | Uncharacterized protein LOC411417 isoform X2 | 1 | -7,35E+14 | 8,77E+14 | 2,54E+14 |
| **134** | A0A7M7H184 | LOC412536 | Leukocyte elastase inhibitor | 23 | -7,66E+13 | 9,32E+14 | 2,67E+14 |
| **135** | A0A7M7MMB8 | LOC726872 | LDLR chaperone boca | 13 | -5,60E+12 | 9,56E+14 | 2,69E+14 |
| **136** | Q1W647 | Obp3 | Odorant binding protein 3 isoform X1 | 9 | -1,14E+14 | 9,64E+14 | 2,69E+14 |
| **137** | A0A7M7R743 | 409341 | Lysosomal aspartic protease | 273 | -8,08E+14 | 9,54E+14 | 2,69E+14 |
| **138** | A0A7M7H3H4 | 725215 | Venom acid phosphatase Acph-1 | 433 | -8,30E+14 | 9,67E+14 | 2,69E+14 |
| **139** | A0A7M7GRI9 | 409849 | Fasciclin-2 isoform X3 | 1 | -5,37E+14 | 9,86E+14 | 2,73E+14 |
| **140** | A0A7M7IGQ7 | LOC100576326 | Phenoloxidase-activating factor 2 isoform X1 | 1 | -1,29E+14 | 1,00E+14 | 2,75E+14 |
| **141** | A0A7M7FXY2 | LOC725038 | NPC intracellular cholesterol transporter 2 homolog a | 178 | -1,46E+14 | 1,03E+14 | 2,78E+14 |
| **142** | A0A7M7MGP7 | LOC100576383 | alpha-1,2-Mannosidase | 1 | -6,75E+14 | 1,02E+13 | 2,78E+14 |
| **143** | A0A7M7GMF0 | 552756 | Cathepsin L1 | 210 | -7,59E+14 | 1,03E+14 | 2,78E+14 |
| **144** | A0A7M7IJ14 | LOC726352 | Transmembrane protease serine 11B-like protein | 23 | -1,18E+14 | 1,10E+14 | 2,92E+14 |
| **145** | A0A7M7M5Q4 | LOC100577163 | Insulin-like growth factor-binding protein complex acid labile subunit | 15 | -1,21E+14 | 1,10E+14 | 2,92E+14 |
| **146** | A0A7M7GL87 | LOC410975 | Uncharacterized protein LOC410975 isoform X1 | 1 | -6,38E+14 | 1,12E+14 | 2,96E+14 |
| **147** | A0A7M7MPK9 | LOC725048 | Nodal modulator 3 isoform X2 | 1 | -4,98E+14 | 1,18E+13 | 3,01E+14 |
| **148** | A0A7M7MTL0 | CUTA | Protein CutA homolog isoform X1 | 18 | -5,59E+14 | 1,16E+14 | 3,01E+14 |
| **149** | A0A7M7R5W7 | 411943 | glutaminyl-peptide cyclotransferase | 10 | -5,92E+14 | 1,18E+14 | 3,01E+14 |
| **150** | A0A7M6UMJ2 | Obp13 | OBP13 | 75 | -7,18E+14 | 1,17E+14 | 3,01E+14 |
| **151** | A0A7M7R2L6 | 408572 | Transgelin | 21 | -7,76E+14 | 1,16E+14 | 3,01E+14 |
| **152** | A0A7M7TF41 | 409999 | Lipase | 1 | -4,87E+13 | 1,23E+14 | 3,13E+14 |
| **153** | Q6W3E3 | Mrjp6 | Bee-milk protein | 33 | -8,60E+14 | 1,25E+14 | 3,15E+14 |
| **154** | R4TRB9 | LOC113218576 | LYS1 | 475 | -7,66E+14 | 1,31E+13 | 3,28E+14 |
| **155** | A0A7M7SRZ1 | LOC113219268 | Uncharacterized protein LOC113219268 isoform X1 | 1 | 6,90E+14 | 1,33E+14 | 3,28E+14 |
| **156** | A0A7M7GAR4 | 100578168 | Fucosyltransferase | 1 | -6,66E+14 | 1,33E+14 | 3,28E+14 |
| **157** | A0A7M6UEA8 | Ppib | Peptidyl-prolyl cis-trans isomerase | 1484 | -6,86E+14 | 1,37E+14 | 3,36E+14 |
| **158** | A0A7M7GLE9 | 552191 | Endoplasmic reticulum resident protein 44 isoform X2 | 1 | -5,56E+14 | 1,40E+14 | 3,40E+14 |
| **159** | A0A7M7R370 | LOC409026 | Transmembrane emp24 domain-containing protein bai | 13 | -6,70E+14 | 1,41E+14 | 3,40E+14 |
| **160** | A0A7M7R608 | 412150 | Heat shock protein 83 | 325 | -7,64E+14 | 1,41E+14 | 3,40E+14 |
| **161** | A0A7M7FZ28 | LOC724644 | Uncharacterized protein LOC724644 | 244 | -1,18E+14 | 1,44E+13 | 3,43E+14 |
| **162** | A0A7M7MQW1 | LOC552683 | Chitinase domain-containing protein 1 | 146 | -6,55E+14 | 1,44E+13 | 3,43E+14 |
| **163** | A0A7M7GXK4 | 413732 | Golgi apparatus protein 1 isoform X2 | 1 | 1,26E+14 | 1,45E+14 | 3,43E+14 |
| **164** | A0A7M7M4R3 | LOC413324 | Chitinase-like protein Idgf4 isoform X2 | 26 | -5,35E+14 | 1,50E+14 | 3,50E+14 |
| **165** | A0A7M7TFG9 | 413429 | Purple acid phosphatase | 82 | -6,13E+14 | 1,51E+14 | 3,50E+14 |
| **166** | A0A7M7TFL1 | LOC409911 | Protein disulfide-isomerase | 321 | -6,73E+14 | 1,51E+14 | 3,50E+14 |
| **167** | A0A7M7M5A5 | 107965322 | Gamma-interferon-inducible lysosomal thiol reductase | 100 | -8,46E+14 | 1,52E+14 | 3,50E+14 |
| **168** | A0A7M7GC96 | LOC410733 | Glucose dehydrogenase [FAD, quinone] | 225 | -7,63E+13 | 1,53E+14 | 3,52E+14 |
| **169** | Q6TGR0 | Mrjp8 | Bee-milk protein | 1399 | -7,09E+14 | 1,64E+13 | 3,65E+14 |
| **170** | P00630 |  | Phospholipase A2 | 8768 | 4,72E+14 | 1,65E+14 | 3,67E+14 |
| **171** | A0A7M7MR23 | LOC727193 | phospholipase A1 | 33 | -7,92E+13 | 1,78E+14 | 3,88E+14 |
| **172** | A0A7M7L6C1 | LOC100577054 | Uncharacterized protein LOC100577054 isoform X1 | 2 | -4,04E+14 | 1,76E+14 | 3,88E+14 |
| **173** | A0A7M7G005 | 726353 | Uncharacterized protein LOC726353 isoform X1 | 1 | -4,31E+14 | 1,78E+14 | 3,88E+14 |
| **174** | A0A7M7R7K3 | 551273 | Lysosomal Pro-X carboxypeptidase | 312 | -6,55E+14 | 1,79E+13 | 3,88E+14 |
| **175** | A0A7M7GAZ8 | 409406 | NTF2-related export protein | 1 | -8,91E+13 | 1,83E+14 | 3,95E+14 |
| **176** | A0A7M7GSG4 | 408474 | Apyrase isoform X1 | 1 | 3,98E+14 | 1,87E+14 | 4,01E+14 |
| **177** | A0A7M7MPW6 | 551793 | Uncharacterized protein LOC551793 isoform X1 | 1 | -5,96E+14 | 1,87E+14 | 4,01E+14 |
| **178** | A0A7M7LRS3 | 550798 | Protein ERGIC-53 | 143 | -5,88E+14 | 1,90E+14 | 4,04E+14 |
| **179** | A0A7M7GIK3 | 410663 | Selenoprotein F | 1 | -5,85E+14 | 1,92E+14 | 4,04E+14 |
| **180** | A0A7M7R5S0 | LOC409365 | Lysosomal alpha-glucosidase | 39 | -6,53E+14 | 1,92E+14 | 4,04E+14 |
| **181** | Q5BLY4 |  | Icarapin-like | 1 | -1,79E+14 | 3,00E+14 | 4,04E+14 |
| **182** | A0A7M7IFF9 | 409167 | Translation elongation factor 2 isoform X1 | 24 | -2,63E+14 | 2,41E+14 | 4,04E+14 |
| **183** | A0A7M7MM69 | LOC412835 | Nucleoporin NUP35 | 76 | -5,99E+13 | 1,98E+14 | 4,10E+14 |
| **184** | A0A7M7MN70 | LOC408618 | Furin-like protease 2 isoform X1 | 2 | -3,93E+14 | 1,99E+14 | 4,10E+14 |
| **185** | A0A7M7GM92 | LOC411748 | LOW QUALITY PROTEIN: protein kinase C-binding protein NELL1 | 74 | -5,72E+14 | 1,98E+14 | 4,10E+14 |
| **186** | A0A7M7GSN5 | 102654987 | Ras-related protein Rab-1A | 24 | -6,63E+14 | 1,98E+14 | 4,10E+14 |
| **187** | A0A7M7MQM2 | LOC409307 | Uncharacterized protein LOC409307 | 41 | -5,69E+14 | 2,01E+14 | 4,13E+14 |
| **188** | A0A7M7GNS0 | 409004 | Ras-related protein Rab6 isoform X1 | 1 | -4,51E+14 | 2,04E+14 | 4,15E+14 |
| **189** | A0A7M7TFY7 | 552747 | Glucosidase 2 subunit beta | 141 | -5,93E+14 | 2,04E+14 | 4,15E+14 |
| **190** | A0A7M7MSM4 | LOC409257 | Uncharacterized protein LOC409257 isoform X1 | 1 | -4,39E+14 | 2,09E+14 | 4,17E+14 |
| **191** | A0A7M7ISX2 | LOC412198 | UDP-glucose:glycoprotein glucosyltransferase isoform X1 | 14 | -4,39E+14 | 2,14E+14 | 4,17E+14 |
| **192** | A0A087ZYX8 | LOC409143 | Venom serine protease 34 isoform X1 | 1 | -4,61E+14 | 2,15E+14 | 4,17E+14 |
| **193** | A0A7M7ILH0 | LOC410362 | MOXD1 homolog 2 | 214 | -6,04E+14 | 2,10E+14 | 4,17E+14 |
| **194** | A0A7M7IHP6 | 410234 | UDP-glucuronate decarboxylase | 62 | -6,65E+14 | 2,12E+14 | 4,17E+14 |
| **195** | A0A7M7GNJ2 | LOC552018 | tRNA (guanine(26)-N(2))-dimethyltransferase | 26 | -6,76E+14 | 2,13E+14 | 4,17E+14 |
| **196** | A0A7M7GUX7 | LOC552663 | Pyridoxal kinase | 11 | -7,82E+14 | 2,14E+14 | 4,17E+14 |
| **197** | A0A7M7GMA7 | 408412 | Prohibitin | 9 | -7,85E+14 | 2,10E+14 | 4,17E+14 |
| **198** | A0A7M7GTZ8 | LOC102655329 | 14-3-3 protein zeta isoform X1 | 1 | -8,01E+14 | 2,11E+14 | 4,17E+14 |
| **199** | A0A7M6UME9 | 406154 | Profilin | 9 | -3,51E+14 | 2,17E+14 | 4,19E+14 |
| **200** | A0A7M7R3G4 | 409265 | Dolichyl-diphosphooligosaccharide--protein glycosyltransferase subunit STT3A | 15 | -4,57E+14 | 2,21E+13 | 4,24E+14 |
| **201** | A0A7M7FZE8 | 725260 | Uncharacterized protein LOC725260 | 41 | -5,57E+14 | 2,23E+14 | 4,25E+14 |
| **202** | A0A7M7MQZ9 | LOC409474 | Laminin subunit gamma-1 | 393 | -5,80E+14 | 2,24E+13 | 4,25E+14 |
| **203** | A0A7M6UFV3 | 100302106 | Uncharacterized protein LOC100302106 | 14 | -4,65E+14 | 2,28E+14 | 4,31E+14 |
| **204** | A0A7M7GLV6 | 409165 | Calreticulin | 370 | -5,80E+14 | 2,31E+14 | 4,32E+14 |
| **205** | A0A7M7R6A3 | LOC409945 | Aspartate--tRNA ligase, cytoplasmic | 5 | -6,89E+14 | 2,31E+14 | 4,32E+14 |
| **206** | A0A7M7R625 | 412886 | Tubulin alpha chain | 1 | -4,08E+14 | 4,34E+14 | 4,39E+14 |
| **207** | A0A7M7IJY0 | LOC409714 | protein-tyrosine-phosphatase | 1 | -5,00E+14 | 2,38E+14 | 4,41E+14 |
| **208** | A0A7M7M5C7 | 410451 | Carboxypeptidase | 124 | -5,39E+14 | 2,37E+14 | 4,41E+14 |
| **209** | A0A7M7L2X7 | LOC412103 | Transport and Golgi organization protein 1 isoform X4 | 1 | -3,80E+14 | 2,41E+14 | 4,44E+14 |
| **210** | A0A7M7FZV4 | 725931 | Multiple inositol polyphosphate phosphatase 1 | 17 | -4,63E+14 | 2,43E+14 | 4,45E+14 |
| **211** | A0A7M7MM50 | LOC409814 | Beta-glucuronidase | 106 | -5,79E+14 | 2,43E+13 | 4,45E+14 |
| **212** | A0A7M7LNL1 | 412460 | cystathionine gamma-lyase | 1 | -4,15E+14 | 2,47E+14 | 4,49E+14 |
| **213** | A0A7M7H507 | LOC552141 | Beta-1,4-N-acetylgalactosaminyltransferase | 40 | -4,10E+14 | 2,48E+13 | 4,49E+14 |
| **214** | P35527 | KRT9 | Keratin, type I cytoskeletal 9 | 374 | 1,35E+14 | 2,52E+14 | 4,54E+14 |
| **215** | A0A7M7MQR2 | LOC100578030 | Antitrypsin isoform X2 | 1 | -4,11E+14 | 2,53E+14 | 4,54E+14 |
| **216** | A0A7M7GY33 | 725882 | Uncharacterized protein LOC725882 | 35 | -4,34E+14 | 2,54E+14 | 4,55E+14 |
| **217** | A0A7M7H280 | 408987 | Sortilin-related receptor | 43 | -4,13E+14 | 2,63E+14 | 4,63E+14 |
| **218** | A0A7M7L4E2 | LOC408826 | Dystroglycan 1 | 1 | -5,57E+14 | 2,62E+13 | 4,63E+14 |
| **219** | A0A7M7FZ07 | LOC726429 | Uncharacterized protein LOC726429 | 38 | -6,71E+14 | 2,63E+13 | 4,63E+14 |
| **220** | A0A7M7LQ41 | LOC412045 | Dolichyl-diphosphooligosaccharide--protein glycosyltransferase subunit 2 | 113 | -4,85E+14 | 2,69E+14 | 4,64E+14 |
| **221** | A0A7M7R3I2 | LOC409298 | Formylglycine-generating enzyme | 18 | -5,01E+14 | 2,68E+14 | 4,64E+14 |
| **222** | A0A7M7L6P4 | LOC551664 | Palmitoyl-protein thioesterase 1 | 310 | -5,64E+14 | 2,67E+13 | 4,64E+14 |
| **223** | A0A7M7MP33 | LOC413792 | Nucleoprotein TPR isoform X1 | 1 | -7,13E+14 | 2,68E+13 | 4,64E+14 |
| **224** | A0A7M7R7N1 | 411799 | Polypeptide N-acetylgalactosaminyltransferase | 3 | 6,09E+14 | 2,81E+14 | 4,68E+14 |
| **225** | A0A7M7H6B6 | 727510 | Lactosylceramide 4-alpha-galactosyltransferase isoform X2 | 1 | -3,63E+14 | 2,79E+14 | 4,68E+14 |
| **226** | A0A7M7G8H6 | LOC726320 | Serine protease inhibitor 27A isoform X1 | 1 | -3,90E+14 | 2,74E+14 | 4,68E+14 |
| **227** | Q8I6X7 | CSP3 | Antennal-specific protein 3c | 36 | -4,35E+14 | 2,75E+14 | 4,68E+14 |
| **228** | A0A7M7MSF1 | LOC551913 | Protein CREG1 | 229 | -5,14E+14 | 2,79E+14 | 4,68E+14 |
| **229** | A0A7M7L1N9 | 726269 | Glutathione peroxidase | 278 | -5,16E+14 | 2,76E+14 | 4,68E+14 |
| **230** | A0A7M7GXL7 | LOC552223 | DnaJ homolog shv | 478 | -5,38E+14 | 2,77E+14 | 4,68E+14 |
| **231** | A0A7M7H012 | LOC724993 | Uncharacterized protein LOC724993 | 18 | -8,27E+14 | 2,80E+14 | 4,68E+14 |
| **232** | A0A7M6UGK8 | Rpn1 | Dolichyl-diphosphooligosaccharide--protein glycosyltransferase subunit 1 | 115 | -4,44E+14 | 2,82E+14 | 4,68E+14 |
| **233** | A0A7M7R554 | 408327 | Uncharacterized protein LOC408327 | 312 | -4,92E+14 | 2,93E+14 | 4,83E+14 |
| **234** | A0A7M7LIH2 | 726495 | Protein D2 | 10 | 4,91E+13 | 3,00E+14 | 4,93E+14 |
| **235** | A0A7M7R8D8 | 411647 | Calumenin | 268 | -4,72E+14 | 3,02E+13 | 4,95E+14 |
| **236** | A0A7M7IKU1 | LOC726698 | Serine protease snake | 73 | -6,27E+14 | 3,05E+14 | 4,98E+14 |
| **237** | A0A7M7FZJ9 | LOC725926 | Collagen alpha-1(IV) chain isoform X2 | 1 | 4,06E+14 | 3,08E+14 | 4,99E+14 |
| **238** | A0A7M7IRY4 | 410769 | Aminopeptidase | 1 | -7,22E+14 | 3,09E+13 | 4,99E+14 |
| **239** | A0A7M7ISB8 | 552086 | Aldose 1-epimerase | 1 | -3,12E+14 | 3,12E+14 | 4,99E+14 |
| **240** | A0A7M7MMZ2 | LOC411344 | Fasciclin-1 isoform X4 | 1 | -3,41E+14 | 3,11E+14 | 4,99E+14 |
| **241** | A0A7M7H421 | LOC725899 | Alpha-galactosidase | 25 | -4,93E+14 | 3,12E+14 | 4,99E+14 |
| **242** | A0A7M7KYY1 | LOC725591 | Protein crumbs isoform X2 | 1 | 4,32E+14 | 3,15E+14 | 5,02E+14 |
| **243** | Q17058 |  | Alpha-glucosidase | 111 | -5,41E+13 | 3,19E+14 | 5,03E+14 |
| **244** | Q5BLY5 |  | Venom acid phosphatase Acph-1 | 1 | -4,13E+14 | 3,19E+14 | 5,03E+14 |
| **245** | A0A7M7LT84 | 100578782 | Complex III subunit 9 | 29 | 3,87E+14 | 3,28E+14 | 5,11E+14 |
| **246** | A0A7M7R6H0 | 408851 | Cysteine proteinase CG12163 isoform X2 | 114 | -4,18E+14 | 3,27E+14 | 5,11E+14 |
| **247** | A0A7M7G0D7 | 727126 | Uncharacterized protein LOC727126 | 37 | -5,44E+14 | 3,28E+14 | 5,11E+14 |
| **248** | A0A7M7GQS0 | 551603 | Ras-related protein Rab-11A | 58 | -3,87E+14 | 3,34E+14 | 5,14E+14 |
| **249** | A0A7M7M3X6 | 408666 | Uncharacterized protein LOC408666 | 1614 | -5,20E+14 | 3,35E+14 | 5,15E+14 |
| **250** | A0A7M7GXX6 | 727390 | Uncharacterized protein LOC727390 isoform X2 | 1 | -4,29E+14 | 3,37E+14 | 5,16E+14 |
| **251** | A0A7M7TFR2 | LOC550884 | N-sulphoglucosamine sulphohydrolase | 133 | -4,29E+14 | 3,45E+14 | 5,21E+14 |
| **252** | A0A7M7GRZ5 | 409668 | Proteasome inhibitor PI31 subunit | 1 | -3,81E+14 | 3,48E+14 | 5,22E+14 |
| **253** | O97432 | MRJP5 | Major royal jelly protein 5 | 117 | -4,93E+14 | 3,47E+14 | 5,22E+14 |
| **254** | A0A7M7SQQ4 | 406133 | Bee-milk protein | 1 | -6,63E+14 | 3,55E+14 | 5,31E+14 |
| **255** | A0A7M7GSJ6 | 414035 | Lachesin isoform X2 | 1 | -6,07E+14 | 3,60E+14 | 5,36E+14 |
| **256** | H9KQJ7 |  | Omega-conotoxin-like protein 1 | 135 | 6,43E+14 | 3,64E+13 | 5,40E+14 |
| **257** | P02768 | ALB | Albumin | 134 | 4,79E+14 | 3,72E+14 | 5,49E+14 |
| **258** | A0A7M7GTG7 | 552602 | Transmembrane emp24 domain-containing protein | 26 | -3,50E+14 | 3,74E+14 | 5,49E+14 |
| **259** | A0A7M7LKE8 | LOC724436 | phospholipase A2 | 545 | -4,74E+14 | 3,74E+14 | 5,49E+14 |
| **260** | P10599 | TXN | Thioredoxin | 6 | -5,04E+14 | 3,76E+14 | 5,50E+14 |
| **261** | A0A7M7R3H9 | LOC409278 | Apolipoprotein D | 21 | -4,64E+14 | 3,79E+14 | 5,53E+14 |
| **262** | A0A7M7G9Y8 | 100577844 | Uncharacterized protein LOC100577844 isoform X1 | 1 | -2,77E+14 | 3,81E+14 | 5,53E+14 |
| **263** | A0A7M7L8V2 | LOC725169 | Succinate--CoA ligase [GDP-forming] subunit beta, mitochondrial | 41 | -4,43E+14 | 3,83E+14 | 5,54E+14 |
| **264** | A0A7M7R3V2 | LOC409663 | lysozyme | 121 | -3,52E+14 | 3,90E+14 | 5,55E+14 |
| **265** | A0A7M7MGK9 | LOC552249 | Alpha-mannosidase | 1 | -3,91E+14 | 3,88E+14 | 5,55E+14 |
| **266** | A0A7M7MNA5 | LOC409281 | Protein sel-1 homolog 1 | 43 | -4,45E+14 | 3,89E+14 | 5,55E+14 |
| **267** | A0A7M7GP09 | LOC102655788 | Uncharacterized protein LOC102655788 | 203 | -4,65E+14 | 3,86E+12 | 5,55E+14 |
| **268** | Q9U8X6 | LOC406081 | Glucose oxidase | 96 | -5,16E+14 | 3,85E+14 | 5,55E+14 |
| **269** | A0A7M7R2Y3 | 408621 | ER membrane protein complex subunit 7 | 57 | -3,87E+14 | 3,92E+14 | 5,56E+14 |
| **270** | O77727 | KRT15 | Keratin, type I cytoskeletal 15 | 27 | 5,95E+14 | 4,01E+14 | 5,65E+14 |
| **271** | P13645 | KRT10 | Keratin, type I cytoskeletal 10 | 211 | 8,86E+14 | 4,06E+13 | 5,69E+14 |
| **272** | A0A7M7MTB6 | 406146 | Hyaluronidase | 133 | -3,50E+14 | 4,08E+14 | 5,70E+14 |
| **273** | A0A7M7GZX8 | LOC725078 | Uncharacterized protein LOC725078 isoform X1 | 1 | -2,74E+14 | 4,10E+14 | 5,71E+14 |
| **274** | A0A7M7GZH3 | 409488 | Protein-L-isoaspartate O-methyltransferase | 1 | -2,81E+14 | 4,15E+14 | 5,75E+14 |
| **275** | A0A7M7IG95 | LOC411864 | Chaoptin | 21 | -4,18E+14 | 4,16E+14 | 5,75E+14 |
| **276** | A0A7M7L992 | LOC100577764 | small monomeric GTPase | 4 | -2,88E+14 | 4,19E+14 | 5,78E+14 |
| **277** | A0A7M7IGX3 | LOC410986 | Tolloid-like protein 1 isoform X5 | 1 | 2,60E+14 | 4,27E+14 | 5,84E+14 |
| **278** | A0A7M6W880 | MRJP9 | Bee-milk protein | 2026 | -3,49E+14 | 4,29E+14 | 5,85E+14 |
| **279** | A0A7M7G9V0 | 100576467 | 14 kDa phosphohistidine phosphatase | 13 | -3,30E+14 | 4,38E+14 | 5,93E+14 |
| **280** | A0A7M7IKX6 | LOC410801 | Cathepsin L1 | 178 | -3,92E+14 | 4,39E+14 | 5,93E+14 |
| **281** | A0A7M6UUL3 | Obp17 | OBP17 | 29 | -4,49E+14 | 4,36E+14 | 5,93E+14 |
| **282** | P35908 | KRT2 | Keratin, type II cytoskeletal 2 epidermal | 183 | 6,70E+14 | 4,46E+14 | 5,97E+14 |
| **283** | A0A7M7IMS8 | LOC551688 | Band 7 protein AGAP004871 isoform X4 | 1 | 4,76E+14 | 4,46E+14 | 5,97E+14 |
| **284** | A0A7M7GEI7 | LOC100578816 | Uncharacterized protein LOC100578816 | 19 | 4,03E+14 | 4,48E+14 | 5,97E+14 |
| **285** | P85831 |  | Prohormone-4 | 28 | -2,84E+14 | 4,46E+14 | 5,97E+14 |
| **286** | A0A7M7MU23 | LOC551651 | Ferritin | 305 | -3,40E+14 | 4,51E+14 | 6,00E+14 |
| **287** | A0A7M7GBS8 | LOC726252 | Titin isoform X3 | 1 | -3,14E+14 | 4,53E+14 | 6,00E+14 |
| **288** | P56587 |  | Tertiapin | 142 | 3,61E+14 | 4,59E+14 | 6,06E+14 |
| **289** | A0A7M7MMJ3 | LOC551807 | Neurexin-4 isoform X4 | 1 | -2,28E+14 | 4,63E+14 | 6,09E+14 |
| **290** | A0A7M7GPD9 | LOC551012 | Cation-independent mannose-6-phosphate receptor isoform X1 | 1 | -5,27E+14 | 4,68E+14 | 6,14E+14 |
| **291** | A0A7M7GQU7 | LOC412838 | Alpha-mannosidase | 16 | -2,55E+14 | 4,71E+14 | 6,16E+14 |
| **292** | A0A7M7R7Z9 | 551684 | Ferritin | 152 | -3,01E+14 | 4,76E+14 | 6,20E+14 |
| **293** | A0A7M7MYD9 | LOC113219418 | Uncharacterized protein LOC113219418 | 13 | -3,37E+14 | 4,84E+14 | 6,29E+14 |
| **294** | A0A7M7H3B9 | 544670 | Elongation factor 1-alpha | 15 | -1,53E+14 | 7,91E+14 | 6,39E+14 |
| **295** | A0A7M7L4T5 | LOC411978 | Myrosinase 1 | 8 | -4,38E+14 | 5,02E+14 | 6,48E+14 |
| **296** | A0A7M7GTW3 | LOC102655078 | LOW QUALITY PROTEIN: zonadhesin | 9 | 3,80E+14 | 5,17E+14 | 6,65E+14 |
| **297** | P04264 | KRT1 | Keratin, type II cytoskeletal 1 | 518 | 8,21E+14 | 5,39E+14 | 6,86E+14 |
| **298** | A0A7M7L949 | LOC413012 | EH domain-containing protein 3 | 21 | -2,29E+14 | 5,35E+14 | 6,86E+14 |
| **299** | A0A7M7R699 | 413167 | Melanotransferrin | 12 | -2,89E+14 | 5,40E+14 | 6,86E+14 |
| **300** | A0A7M7R9P8 | 551250 | Microsomal triglyceride transfer protein large subunit | 16 | -2,93E+14 | 5,39E+14 | 6,86E+14 |
| **301** | A0A7M7GQX8 | 408808 | Prosaposin isoform X2 | 16 | 4,47E+14 | 5,45E+13 | 6,87E+14 |
| **302** | A0A7M7GRE8 | LOC413987 | Polypeptide N-acetylgalactosaminyltransferase | 4 | -2,53E+14 | 5,46E+14 | 6,87E+14 |
| **303** | A0A7M7GMY7 | 408951 | 14-3-3 protein epsilon | 8 | -3,46E+14 | 5,45E+14 | 6,87E+14 |
| **304** | A0A7M7TF36 | 410539 | Protein 5NUC | 184 | -2,75E+14 | 5,51E+14 | 6,91E+14 |
| **305** | Q25BT7 | Hbg2 | alpha-glucosidase | 174 | -2,08E+13 | 1,71E+14 | 6,95E+14 |
| **306** | A0A7M6UMR7 | Hex110 | Hexamerin 110 precursor | 5 | -1,54E+14 | 1,36E+14 | 6,95E+14 |
| **307** | A0A7M7GXT7 | LOC409533 | Annexin | 1 | -1,56E+14 | 1,70E+14 | 6,95E+14 |
| **308** | B0LUE8 | A4 | Apolipophorin-III-like protein precursor | 107 | -2,01E+14 | 1,44E+14 | 6,95E+14 |
| **309** | A0A7M7GJ95 | LOC100577156 | Chitinase-3-like protein 1 | 3 | -2,29E+14 | 1,72E+14 | 6,95E+14 |
| **310** | A0A7M7LRW3 | LOC102656570 | Glucose dehydrogenase [FAD, quinone] | 171 | -2,97E+14 | 5,59E+14 | 6,99E+14 |
| **311** | A0A7M7GER9 | LOC552798 | Sodium-coupled neutral amino acid transporter 10 isoform X1 | 1 | 2,85E+14 | 5,60E+14 | 6,99E+14 |
| **312** | Q8MQS8 |  | Venom serine protease 34 | 1 | -2,26E+14 | 5,62E+14 | 6,99E+14 |
| **313** | A0A7M7GAF4 | 100576444 | Chymotrypsin inhibitor | 246 | 4,22E+14 | 5,70E+14 | 7,03E+14 |
| **314** | A0A7M7R5K0 | 409709 | Glucosylceramidase | 57 | -2,41E+14 | 5,73E+14 | 7,03E+14 |
| **315** | A0A7M7MVN9 | LOC409366 | Integral membrane protein 2 | 25 | -3,74E+14 | 5,72E+14 | 7,03E+14 |
| **316** | A0A7M7H2N8 | 409949 | Agrin | 33 | -3,85E+14 | 5,73E+14 | 7,03E+14 |
| **317** | P56682 |  | Chymotrypsin inhibitor | 49 | -4,92E+14 | 5,78E+14 | 7,08E+14 |
| **318** | A0A7M7RA46 | LOC552154 | Uncharacterized protein LOC552154 | 19 | 2,17E+14 | 5,81E+14 | 7,09E+14 |
| **319** | P17722 |  | Defensin-1 | 15 | -2,79E+14 | 5,85E+14 | 7,11E+14 |
| **320** | A0A7M7MMK3 | LOC113218958 | Superoxide dismutase [Cu-Zn] | 41 | 2,85E+14 | 5,93E+14 | 7,15E+14 |
| **321** | A0A7M7L685 | LOC724890 | Laccase-1 | 12 | -2,50E+14 | 5,91E+14 | 7,15E+14 |
| **322** | A0A7M7R8L1 | 411890 | Nucleolysin TIAR | 4 | -2,86E+14 | 5,90E+14 | 7,15E+14 |
| **323** | A0A7M7G858 | LOC725661 | Uncharacterized protein LOC725661 | 127 | -2,08E+11 | 6,11E+14 | 7,32E+14 |
| **324** | A0A7M7GV42 | 408851 | Cysteine proteinase CG12163 isoform X1 | 9 | -2,29E+13 | 6,12E+14 | 7,32E+14 |
| **325** | P00761 |  | Trypsin | 1249 | -2,34E+14 | 6,12E+14 | 7,32E+14 |
| **326** | A0A7M7MM38 | LOC411392 | Protein Red | 22 | -3,50E+14 | 6,17E+14 | 7,35E+14 |
| **327** | A0A7M7GYC6 | 724900 | Uncharacterized protein LOC724900 isoform X3 | 1 | -3,94E+14 | 6,20E+14 | 7,37E+14 |
| **328** | A0A7M7GSP5 | LOC100577777 | Cysteine-rich venom protein 6 isoform X2 | 1 | -1,71E+14 | 6,26E+13 | 7,41E+14 |
| **329** | A0A7M7G3D2 | 727309 | Glutaredoxin-C4 isoform X2 | 17 | 2,30E+14 | 6,32E+14 | 7,47E+14 |
| **330** | A0A7M7RB96 | 551968 | Aldose reductase | 35 | -1,80E+14 | 6,34E+14 | 7,47E+14 |
| **331** | A0A7M7R8L8 | LOC413614 | Ras-related protein Rab-14 | 6 | -1,43E+13 | 6,37E+13 | 7,48E+14 |
| **332** | A0A7M7MVC3 | LOC724421 | Fibrillin-2 | 66 | -4,06E+14 | 6,39E+14 | 7,49E+14 |
| **333** | A0A7M7GQ39 | LOC408937 | SPARC | 51 | -2,37E+14 | 6,49E+14 | 7,58E+14 |
| **334** | Q10416 |  | Hymenoptaecin | 19 | -1,99E+14 | 6,54E+14 | 7,62E+14 |
| **335** | A0A7M7GU71 | LOC552077 | ER membrane protein complex subunit 1 | 1 | -1,26E+14 | 6,70E+14 | 7,78E+14 |
| **336** | A0A7M7TFN1 | 413631 | Vanin-like protein 1 | 30 | 1,48E+14 | 6,86E+14 | 7,84E+14 |
| **337** | A0A7M7IT99 | LOC410890 | Protein seele | 41 | -3,06E+13 | 6,80E+13 | 7,84E+14 |
| **338** | A0A7M7H6R7 | 102654353 | Fucosyltransferase | 95 | -1,70E+14 | 6,87E+14 | 7,84E+14 |
| **339** | A0A7M7GQV1 | LOC102655185 | Uncharacterized protein LOC102655185 isoform X1 | 1 | -1,94E+14 | 6,82E+14 | 7,84E+14 |
| **340** | A0A7M7RBC3 | LOC552734 | Endoribonuclease | 17 | -2,67E+14 | 6,86E+14 | 7,84E+14 |
| **341** | A0A7M7GAL3 | 409451 | Thioredoxin | 28 | -2,89E+14 | 6,81E+14 | 7,84E+14 |
| **342** | A0A7M7L0F8 | LOC552283 | glutathione transferase | 33 | -1,47E+14 | 2,17E+14 | 7,90E+14 |
| **343** | A0A7M7L6X9 | LOC726286 | Uncharacterized protein LOC726286 | 25 | -1,53E+14 | 2,35E+14 | 7,90E+14 |
| **344** | A0A7M7INZ2 | 412523 | Out at first protein | 29 | 1,88E+13 | 6,99E+14 | 7,96E+14 |
| **345** | A0A7M7ML78 | LOC408953 | Peroxidase isoform X3 | 1 | -1,10E+14 | 3,59E+14 | 8,07E+14 |
| **346** | A0A7M7GWX8 | LOC100578909 | Procollagen-lysine,2-oxoglutarate 5-dioxygenase 3 isoform X1 | 1 | -1,26E+14 | 4,20E+14 | 8,07E+14 |
| **347** | A0A7M7H289 | 727146 | Sphingomyelin phosphodiesterase | 1 | -1,26E+14 | 3,66E+14 | 8,07E+14 |
| **348** | A0A7M7GQ31 | LOC726818 | Beta-hexosaminidase | 37 | -1,27E+14 | 3,90E+14 | 8,07E+14 |
| **349** | A0A7M7R985 | 413489 | Deoxyribonuclease-2-alpha | 40 | -1,37E+14 | 2,83E+14 | 8,07E+14 |
| **350** | A0A7M6UVZ7 | 727472 | Peptidoglycan-recognition protein | 64 | -1,45E+14 | 4,03E+14 | 8,07E+14 |
| **351** | Q1W633 | Obp21 | Odorant binding protein 21 precursor | 78 | -1,57E+14 | 3,30E+14 | 8,07E+14 |
| **352** | A0A7M6UW32 | 406122 | Actin related protein 1 | 51 | -1,86E+14 | 3,65E+14 | 8,07E+14 |
| **353** | A0A7M7LT78 | LOC102654405 | Uncharacterized protein LOC102654405 | 26 | -2,63E+14 | 3,80E+14 | 8,07E+14 |
| **354** | B2D0J4 |  | Venom dipeptidyl peptidase 4 | 41 | -2,23E+13 | 7,17E+14 | 8,13E+14 |
| **355** | A0A7M7GY19 | LOC100577073 | Pigment-dispersing hormone peptides | 2 | -1,19E+14 | 7,20E+14 | 8,14E+14 |
| **356** | A0A088AA06 | 552398 | Hexosyltransferase | 1 | -1,25E+14 | 7,24E+14 | 8,17E+14 |
| **357** | A0A7M7RB56 | 552645 | Mesencephalic astrocyte-derived neurotrophic factor homolog | 32 | -1,27E+14 | 7,33E+14 | 8,20E+14 |
| **358** | A0A7M7GXZ4 | LOC726552 | NEDD8 | 5 | -1,32E+14 | 7,34E+14 | 8,20E+14 |
| **359** | A0A7M7H0K2 | 552068 | Protein phosphatase 1L isoform X2 | 1 | -1,49E+14 | 7,28E+14 | 8,20E+14 |
| **360** | P61626 | LYZ | Lysozyme C | 70 | -2,41E+14 | 7,33E+14 | 8,20E+14 |
| **361** | A0A7M7GFE2 | LOC100577168 | Uncharacterized protein LOC100577168 | 37 | -1,30E+14 | 7,40E+13 | 8,24E+14 |
| **362** | A0A7M7GZD2 | LOC408568 | Peptidyl-alpha-hydroxyglycine alpha-amidating lyase 1 isoform X4 | 1 | -1,07E+14 | 7,44E+14 | 8,26E+14 |
| **363** | A0A7M7GBM8 | 100577847 | Uncharacterized protein LOC100577847 isoform X1 | 1 | -1,34E+13 | 8,54E+14 | 8,27E+14 |
| **364** | A0A7M7L1T2 | LOC552572 | Eukaryotic translation initiation factor 5A | 23 | -1,23E+14 | 8,21E+14 | 8,27E+14 |
| **365** | A0A7M7MQS4 | LOC100578030 | Antichymotrypsin-2 isoform X1 | 1 | -1,26E+14 | 8,60E+14 | 8,27E+14 |
| **366** | A0A7M7FZR7 | LOC725960 | Neurofilament heavy polypeptide | 49 | -2,31E+14 | 8,09E+14 | 8,27E+14 |
| **367** | B2D0J5 |  | Venom carboxylesterase-6 | 2426 | 1,28E+14 | 7,51E+14 | 8,31E+14 |
| **368** | A0A7M7LQ38 | 551227 | Myb-like protein I isoform X2 | 1 | 1,95E+14 | 7,56E+14 | 8,32E+14 |
| **369** | A0A7M7H1B6 | 551749 | LARGE xylosyl- and glucuronyltransferase 1 | 17 | -1,37E+14 | 7,56E+14 | 8,32E+14 |
| **370** | A0A7M7R586 | 408426 | Lysosomal thioesterase PPT2 homolog isoform X1 | 2 | 1,28E+14 | 7,64E+14 | 8,37E+14 |
| **371** | A0A7M7TFW2 | LOC552428 | Uncharacterized protein LOC552428 | 45 | -1,16E+14 | 7,63E+14 | 8,37E+14 |
| **372** | A0A7M7MTG9 | LOC413579 | alpha-1,2-Mannosidase | 32 | 1,52E+14 | 7,74E+14 | 8,42E+14 |
| **373** | A0A7M7SS83 | LOC552435 | alpha-1,2-Mannosidase | 43 | 1,15E+14 | 7,72E+14 | 8,42E+14 |
| **374** | A0A7M7R7L3 | 412543 | Uncharacterized protein LOC412543 isoform X1 | 14 | -1,09E+14 | 9,12E+14 | 8,56E+14 |
| **375** | A0A7M7R6X2 | LOC409091 | Neurotrimin | 132 | 1,06E+14 | 7,96E+13 | 8,64E+14 |
| **376** | A0A7M7GYE7 | LOC412273 | Probable chitinase 10 isoform X2 | 1 | 1,15E+14 | 8,01E+14 | 8,68E+14 |
| **377** | A0A7M7LN74 | 100578232 | Notch homolog 2 N-terminal-like protein | 5 | 1,28E+14 | 8,08E+14 | 8,71E+14 |
| **378** | A0A088AQF2 | 107965838 | Endoribonuclease | 34 | -9,60E+14 | 8,09E+14 | 8,71E+14 |
| **379** | A0A7M7R7F0 | 413977 | Calsyntenin-1 | 142 | -1,06E+14 | 8,12E+14 | 8,72E+14 |
| **380** | A0A7M7TF95 | 411832 | Translationally-controlled tumor protein homolog | 16 | 1,12E+14 | 8,21E+14 | 8,79E+14 |
| **381** | A0A7M7IQ52 | 551268 | phospholipase A1 | 42 | -7,84E+14 | 8,31E+14 | 8,88E+14 |
| **382** | A0A7M7R8D5 | 411542 | Signal recognition particle receptor subunit alpha homolog | 20 | -1,03E+14 | 8,36E+14 | 8,91E+14 |
| **383** | A0A7M7TFV6 | 551554 | Ras-related protein Rac1 | 1 | 1,10E+14 | 8,57E+13 | 9,11E+14 |
| **384** | A0A7M7IM62 | 552051 | Dolichyl-diphosphooligosaccharide--protein glycosyltransferase 48 kDa subunit | 7 | -9,48E+14 | 8,62E+14 | 9,14E+14 |
| **385** | A0A7M7KZK9 | LOC551170 | Uncharacterized protein LOC551170 isoform X3 | 1 | -7,92E+14 | 8,65E+14 | 9,15E+14 |
| **386** | A0A7M7GPB8 | LOC726421 | Membrane metallo-endopeptidase-like 1 isoform X2 | 1 | 5,19E+14 | 8,71E+13 | 9,16E+14 |
| **387** | A0A7M7IS49 | 552115 | peptidylprolyl isomerase | 16 | 1,05E+14 | 8,71E+14 | 9,16E+14 |
| **388** | A0A7M7FYF7 | LOC725287 | Sulfhydryl oxidase | 27 | -8,14E+14 | 8,78E+14 | 9,21E+14 |
| **389** | A0A7M7MSB6 | LOC410369 | Uncharacterized protein LOC410369 | 13 | 8,81E+13 | 8,90E+14 | 9,31E+14 |
| **390** | A0A7M7IMI7 | 724477 | Vitamin K-dependent protein C isoform X1 | 1 | -4,96E+14 | 8,93E+14 | 9,32E+14 |
| **391** | A0A7M7FZ02 | LOC724703 | Modular serine protease | 320 | -4,98E+14 | 9,15E+14 | 9,53E+14 |
| **392** | A0A7M7H423 | LOC552459 | Glycine-rich cell wall structural protein 1 isoform X2 | 1 | 7,60E+14 | 9,22E+14 | 9,58E+14 |
| **393** | A0A7M7LRU3 | 551859 | Calmodulin | 37 | -1,55E+14 | 1,05E+14 | 9,63E+14 |
| **394** | A0A7M7IRR6 | LOC413324 | Chitinase-like protein EN03 isoform X1 | 8 | 5,04E+14 | 9,38E+14 | 9,64E+14 |
| **395** | A0A7M7GNU1 | LOC725883 | Guanine nucleotide-binding protein subunit gamma-1 | 25 | 2,67E+14 | 9,48E+14 | 9,71E+14 |
| **396** | A0A7M7GTC0 | 412808 | Aminopeptidase | 1 | 3,87E+13 | 9,50E+14 | 9,71E+14 |
| **397** | A0A7M7R9K2 | 552722 | Glyoxalase domain-containing protein 4 | 24 | 3,76E+14 | 9,58E+14 | 9,77E+14 |
| **398** | A0A7M7H375 | 551031 | Uncharacterized protein LOC551031 | 23 | -1,83E+14 | 9,66E+14 | 9,81E+14 |
| **399** | A0A7M7MTF8 | LOC413569 | protein-tyrosine-phosphatase | 1 | -2,04E+14 | 9,65E+14 | 9,81E+14 |
| **400** | A0A7M7LIA2 | 724382 | Protein obstructor-E | 12 | -1,33E+14 | 9,75E+14 | 9,85E+14 |
| **401** | A0A7M7IEU7 | LOC411903 | Dyslexia-associated protein KIAA0319 | 16 | -2,12E+14 | 9,77E+14 | 9,85E+14 |
| **402** | A0A7M7GZ60 | 551617 | Uncharacterized protein LOC551617 | 77 | -1,03E+14 | 9,80E+14 | 9,85E+14 |
| **403** | A0A7M7LM75 | LOC409826 | Proteoglycan 4 isoform X2 | 1 | -1,26E+14 | 9,84E+14 | 9,86E+14 |
| **404** | A0A7M7TFR9 | 551785 | 6-phosphogluconolactonase | 30 | -7,37E+14 | 9,88E+14 | 9,88E+14 |

**Supplementary Table 3.** Differentially abundant proteins between AF and SL.
Results of differential abundance analysis performed using DEqMS comparing protein abundance profiles between the AF and SL geographic zones. The table includes UniProt accession numbers, gene names, protein descriptions, peptide counts, log fold change (logFC), raw *P* values, and false discovery rate (FDR)-adjusted *P* values. Proteins are ranked according to FDR-adjusted *P* value in ascending order.

| **N°** | **Protein.ID** | **Gene** | **Description** | **Count** | **logFC** | **P Value** | **FDR-adjusted P value** |
| --- | --- | --- | --- | --- | --- | --- | --- |
| **1** | A0A7M7ML78 | LOC408953 | Peroxidase isoform X3 | 1 | -6,50E+14 | 5,10E+14 | 3,22E+12 |
| **2** | A0A7M7MMJ3 | LOC551807 | Neurexin-4 isoform X4 | 1 | -2,46E+13 | 4,29E+14 | 7,38E+12 |
| **3** | A0A7M7R699 | 413167 | Melanotransferrin | 12 | -3,77E+14 | 4,28E+14 | 7,38E+12 |
| **4** | P13645 | KRT10 | Keratin, type I cytoskeletal 10 | 211 | 1,78E+14 | 1,11E+14 | 4,16E+13 |
| **5** | A0A7M7RBA5 | PGAM2 | Phosphoglycerate mutase | 61 | -6,38E+13 | 1,08E+14 | 4,16E+13 |
| **6** | A0A7M7H3B9 | 544670 | Elongation factor 1-alpha | 15 | -5,72E+14 | 1,13E+14 | 4,16E+13 |
| **7** | A0A7M7RA46 | LOC552154 | Uncharacterized protein LOC552154 | 19 | -6,65E+14 | 1,09E+14 | 4,16E+13 |
| **8** | A0A7M6UDU9 | 494523 | Glutathione peroxidase | 1 | -6,71E+14 | 1,08E+14 | 4,16E+13 |
| **9** | A0A7M7G9V0 | 100576467 | 14 kDa phosphohistidine phosphatase | 13 | -7,14E+14 | 1,11E+13 | 4,16E+13 |
| **10** | A0A7M7R5Q5 | 412393 | Cytosolic non-specific dipeptidase | 33 | -7,17E+14 | 1,03E+14 | 4,16E+13 |
| **11** | Q1W633 | Obp21 | Odorant binding protein 21 precursor | 78 | -7,38E+14 | 1,06E+14 | 4,16E+13 |
| **12** | A0A7M7GLV6 | 409165 | Calreticulin | 370 | -7,99E+14 | 1,09E+14 | 4,16E+13 |
| **13** | A0A7M7MP34 | LOC107964964 | Uncharacterized protein LOC107964964 | 21 | -8,00E+14 | 1,03E+13 | 4,16E+13 |
| **14** | A0A7M7R6M6 | 409890 | Peptidyl-prolyl cis-trans isomerase | 84 | -8,26E+14 | 1,13E+14 | 4,16E+13 |
| **15** | A0A7M7R6I4 | 413478 | Spermine oxidase | 53 | -9,60E+14 | 1,07E+13 | 4,16E+13 |
| **16** | A0A7M7TF41 | 409999 | Lipase | 1 | -4,49E+14 | 1,52E+12 | 4,87E+13 |
| **17** | A0A7M7GP09 | LOC102655788 | Uncharacterized protein LOC102655788 | 203 | -7,72E+14 | 1,63E+13 | 5,05E+13 |
| **18** | Q8MQS8 |  | Venom serine protease 34 | 1 | 4,99E+14 | 2,14E+14 | 5,38E+13 |
| **19** | A0A7M7MM52 | LOC413627 | Uncharacterized protein LOC413627 | 96 | -5,55E+13 | 2,11E+14 | 5,38E+13 |
| **20** | A0A7M7R554 | 408327 | Uncharacterized protein LOC408327 | 312 | -5,92E+13 | 2,10E+14 | 5,38E+13 |
| **21** | A0A7M7MPK9 | LOC725048 | Nodal modulator 3 isoform X2 | 1 | -3,88E+14 | 2,13E+14 | 5,38E+13 |
| **22** | A0A7M7MP76 | LOC413274 | guanylate cyclase | 1 | -4,91E+14 | 2,11E+14 | 5,38E+13 |
| **23** | A0A7M7GIK3 | 410663 | Selenoprotein F | 1 | -5,62E+14 | 2,09E+13 | 5,38E+13 |
| **24** | A0A7M7IKC6 | 412394 | alpha-L-fucosidase | 1 | -3,76E+14 | 2,32E+14 | 5,56E+13 |
| **25** | A0A7M7IQZ1 | LOC107965485 | Uncharacterized protein LOC107965485 | 47 | -7,62E+14 | 2,31E+14 | 5,56E+13 |
| **26** | A0A7M7IS49 | 552115 | peptidylprolyl isomerase | 16 | -7,03E+14 | 3,58E+14 | 6,97E+13 |
| **27** | A0A7M7MTG9 | LOC413579 | alpha-1,2-Mannosidase | 32 | 3,93E+14 | 4,63E+14 | 7,62E+13 |
| **28** | A0A7M7GPP4 | LOC725788 | Plexin domain-containing protein 2 isoform X1 | 2 | -3,11E+14 | 4,65E+14 | 7,62E+13 |
| **29** | A0A7M7GUX7 | LOC552663 | Pyridoxal kinase | 11 | -5,19E+14 | 4,62E+14 | 7,62E+13 |
| **30** | A0A7M7H0K2 | 552068 | Protein phosphatase 1L isoform X2 | 1 | -2,95E+14 | 4,96E+14 | 7,76E+13 |
| **31** | A0A7M7MN89 | LOC551373 | Ectonucleoside triphosphate diphosphohydrolase 5 isoform X1 | 1 | -2,41E+14 | 5,15E+14 | 7,92E+13 |
| **32** | A0A7M7GQ60 | 408808 | Prosaposin isoform X1 | 62 | -3,28E+14 | 5,13E+14 | 7,92E+13 |
| **33** | A0A7M7IG95 | LOC411864 | Chaoptin | 21 | 1,90E+13 | 7,07E+14 | 8,50E+13 |
| **34** | A0A7M7R5K0 | 409709 | Glucosylceramidase | 57 | -8,56E+14 | 8,40E+13 | 9,15E+13 |
| **35** | A0A7M7GXX6 | 727390 | Uncharacterized protein LOC727390 isoform X2 | 1 | -8,75E+14 | 8,41E+14 | 9,15E+13 |
| **36** | Q6TGR0 | Mrjp8 | Bee-milk protein | 1399 | -8,24E+12 | 8,65E+14 | 9,24E+13 |
| **37** | A0A7M7H2N8 | 409949 | Agrin | 33 | -1,15E+14 | 8,66E+14 | 9,24E+13 |
| **38** | A0A7M7TF95 | 411832 | Translationally-controlled tumor protein homolog | 16 | -8,47E+14 | 8,64E+13 | 9,24E+13 |
| **39** | A0A088AHC8 | 410122 | Glyceraldehyde-3-phosphate dehydrogenase | 29 | -4,20E+13 | 9,30E+14 | 9,59E+13 |
| **40** | A0A7M7H289 | 727146 | Sphingomyelin phosphodiesterase | 1 | -1,18E+14 | 5,48E+14 | 1,15E+14 |
| **41** | A0A7M7IPH8 | LOC552229 | Carboxylic ester hydrolase | 7 | -1,18E+13 | 6,05E+12 | 1,19E+14 |
| **42** | A0A7M7RC42 | LOC552811 | Laccase-5 | 33 | -1,68E+14 | 6,32E+14 | 1,19E+14 |
| **43** | A0A7M7IL97 | 551044 | Glucose dehydrogenase [FAD, quinone] isoform X2 | 1 | -9,61E+14 | 6,79E+14 | 1,23E+14 |
| **44** | A0A7M7R985 | 413489 | Deoxyribonuclease-2-alpha | 40 | -1,16E+14 | 7,73E+14 | 1,30E+14 |
| **45** | A0A7M7LRU3 | 551859 | Calmodulin | 37 | -1,64E+14 | 7,87E+14 | 1,30E+14 |
| **46** | A0A7M6UEA8 | Ppib | Peptidyl-prolyl cis-trans isomerase | 1484 | -1,37E+14 | 8,60E+14 | 1,37E+14 |
| **47** | P01499 |  | Mast cell degranulating peptide | 180 | 2,85E+14 | 9,00E+14 | 1,37E+14 |
| **48** | A0A7M7LRW3 | LOC102656570 | Glucose dehydrogenase [FAD, quinone] | 171 | -1,56E+14 | 9,32E+14 | 1,37E+14 |
| **49** | A0A7M7R2N0 | LOC408603 | Glucose dehydrogenase [FAD, quinone] | 20 | -2,97E+14 | 1,19E+14 | 1,68E+14 |
| **50** | A0A7M7GBM8 | 100577847 | Uncharacterized protein LOC100577847 isoform X1 | 1 | -2,75E+14 | 4,42E+09 | 1,76E+14 |
| **51** | A0A7M7LKF0 | LOC725273 | Uncharacterized protein LOC725273 isoform X1 | 11 | -8,57E+14 | 1,39E+14 | 1,88E+14 |
| **52** | A0A7M7R673 | 102656890 | Thioredoxin domain-containing protein 17 | 22 | -9,82E+14 | 1,42E+14 | 1,88E+14 |
| **53** | A5YVK7 | Hex70a | Hexamerin 70a precursor | 268 | 1,58E+14 | 1,48E+14 | 1,90E+14 |
| **54** | A0A7M7GAR4 | 100578168 | Fucosyltransferase | 1 | 1,17E+14 | 1,63E+14 | 1,90E+14 |
| **55** | A0A7M7GZ60 | 551617 | Uncharacterized protein LOC551617 | 77 | -1,19E+14 | 1,54E+13 | 1,90E+14 |
| **56** | A0A7M7R625 | 412886 | Tubulin alpha chain | 1 | -2,34E+14 | 1,58E+14 | 1,90E+14 |
| **57** | P61626 | LYZ | Lysozyme C | 70 | 1,92E+14 | 1,80E+14 | 2,04E+14 |
| **58** | A0A7M7GVQ8 | 552382 | Alpha-mannosidase | 1 | -8,11E+14 | 1,87E+14 | 2,07E+14 |
| **59** | A0A7M7GA54 | 100576174 | Uncharacterized protein LOC100576174 | 11 | 1,69E+14 | 1,98E+14 | 2,07E+14 |
| **60** | A0A7M7GL48 | 413256 | Uncharacterized protein LOC413256 | 297 | -1,31E+14 | 1,99E+14 | 2,07E+14 |
| **61** | Q868N5 | Vg | Vitellogenin | 1356 | 1,19E+14 | 2,09E+14 | 2,08E+14 |
| **62** | A0A7M7FZR7 | LOC725960 | Neurofilament heavy polypeptide | 49 | -1,94E+14 | 2,05E+14 | 2,08E+14 |
| **63** | C0HLU0 |  | Secapin-1 | 73 | -1,55E+14 | 2,28E+14 | 2,21E+14 |
| **64** | A0A7M7TFN1 | 413631 | Vanin-like protein 1 | 30 | -9,28E+14 | 2,45E+14 | 2,28E+14 |
| **65** | A0A7M7GL87 | LOC410975 | Uncharacterized protein LOC410975 isoform X1 | 1 | -9,61E+14 | 2,47E+14 | 2,28E+14 |
| **66** | A0A7M7FYU4 | 726196 | Ubiquitin-fold modifier 1 | 10 | -1,03E+14 | 2,65E+14 | 2,35E+14 |
| **67** | A0A7M7G166 | 725813 | Uncharacterized protein LOC725813 | 44 | -9,42E+14 | 2,66E+14 | 2,35E+14 |
| **68** | A0A7M7IHQ8 | 107965279 | phospholipase A1 | 112 | -1,16E+14 | 2,76E+14 | 2,38E+14 |
| **69** | P00761 |  | Trypsin | 1249 | 1,12E+14 | 3,00E+14 | 2,48E+14 |
| **70** | A0A7M7R503 | 408516 | Protein D2 isoform X3 | 26 | -8,22E+14 | 3,00E+12 | 2,48E+14 |
| **71** | A0A7M7GXT7 | LOC409533 | Annexin | 1 | -1,12E+14 | 3,18E+14 | 2,51E+14 |
| **72** | A0A7M7GQV1 | LOC102655185 | Uncharacterized protein LOC102655185 isoform X1 | 1 | -1,14E+14 | 3,29E+14 | 2,51E+14 |
| **73** | A0A7M7GWX8 | LOC100578909 | Procollagen-lysine,2-oxoglutarate 5-dioxygenase 3 isoform X1 | 1 | -8,53E+14 | 3,25E+14 | 2,51E+14 |
| **74** | A0A7M7R7W3 | LOC551763 | Hypoxia up-regulated protein 1 | 27 | -9,34E+14 | 3,17E+14 | 2,51E+14 |
| **75** | P56587 |  | Tertiapin | 142 | -1,14E+14 | 3,43E+14 | 2,57E+14 |
| **76** | A0A7M7GY19 | LOC100577073 | Pigment-dispersing hormone peptides | 2 | 9,46E+14 | 3,80E+14 | 2,62E+14 |
| **77** | A0A7M7III5 | LOC408600 | Nucleobindin-2 | 1074 | -1,07E+14 | 3,83E+14 | 2,62E+14 |
| **78** | A0A7M7GU71 | LOC552077 | ER membrane protein complex subunit 1 | 1 | -6,81E+14 | 3,77E+14 | 2,62E+14 |
| **79** | A0A7M7H494 | 409708 | Glucosylceramidase | 1 | -7,47E+14 | 3,67E+14 | 2,62E+14 |
| **80** | A0A7M7IL03 | LOC102656186 | Trypsin-5 isoform X1 | 53 | -9,32E+14 | 3,79E+14 | 2,62E+14 |
| **81** | Q5BLY4 |  | Icarapin-like | 1 | -1,96E+14 | ######## | 2,78E+14 |
| **82** | A0A088AA06 | 552398 | Hexosyltransferase | 1 | -7,94E+14 | 4,24E+13 | 2,85E+14 |
| **83** | A0A7M7GQ55 | LOC411607 | 4a-hydroxytetrahydrobiopterin dehydratase | 12 | -1,12E+14 | 4,48E+14 | 2,96E+14 |
| **84** | A0A7M7TFS9 | 551922 | NPC intracellular cholesterol transporter 2 homolog a | 142 | -9,85E+14 | 4,58E+14 | 2,98E+14 |
| **85** | A0A7M7SPQ2 | 100577408 | Leukocyte elastase inhibitor | 57 | -8,56E+14 | 4,74E+14 | 3,04E+14 |
| **86** | A0A7M7MQR2 | LOC100578030 | Antitrypsin isoform X2 | 1 | 2,34E+14 | 2,38E+14 | 3,15E+14 |
| **87** | A0A7M7M145 | 725498 | Protein mesh | 34 | -1,12E+14 | 5,24E+14 | 3,23E+14 |
| **88** | A0A7M7GSG4 | 408474 | Apyrase isoform X1 | 1 | -6,05E+14 | 5,59E+14 | 3,23E+14 |
| **89** | A0A7M7G9Y8 | 100577844 | Uncharacterized protein LOC100577844 isoform X1 | 1 | -6,48E+14 | 5,61E+14 | 3,23E+14 |
| **90** | A0A7M7IMI7 | 724477 | Vitamin K-dependent protein C isoform X1 | 1 | -7,74E+14 | 5,51E+14 | 3,23E+14 |
| **91** | A0A7M7TFG9 | 413429 | Purple acid phosphatase | 82 | -8,62E+14 | 5,34E+14 | 3,23E+14 |
| **92** | A0A7M7MQM2 | LOC409307 | Uncharacterized protein LOC409307 | 41 | -8,99E+14 | 5,53E+14 | 3,23E+14 |
| **93** | A0A7M7L685 | LOC724890 | Laccase-1 | 12 | -9,67E+14 | 5,82E+14 | 3,30E+14 |
| **94** | A0A7M7R6Y1 | 409126 | Ras-related protein Rab-2 | 35 | -7,65E+13 | 6,20E+14 | 3,42E+14 |
| **95** | A0A7M7TFL1 | LOC409911 | Protein disulfide-isomerase | 321 | -9,09E+14 | 6,14E+14 | 3,42E+14 |
| **96** | A0A7M7R9E8 | LOC550785 | Fructose-bisphosphate aldolase | 29 | -1,09E+13 | 6,50E+14 | 3,49E+14 |
| **97** | A0A7M7L0F8 | LOC552283 | glutathione transferase | 33 | -7,51E+14 | 6,54E+14 | 3,49E+14 |
| **98** | A0A7M7R3I2 | LOC409298 | Formylglycine-generating enzyme | 18 | -8,81E+14 | 6,68E+14 | 3,49E+14 |
| **99** | A0A7M7R9Q2 | LOC552836 | Uncharacterized protein LOC552836 | 203 | -9,84E+14 | 6,61E+14 | 3,49E+14 |
| **100** | P02768 | ALB | Albumin | 134 | 1,04E+14 | 6,96E+14 | 3,54E+14 |
| **101** | A0A7M7GLE9 | 552191 | Endoplasmic reticulum resident protein 44 isoform X2 | 1 | -7,05E+14 | 6,90E+14 | 3,54E+14 |
| **102** | P02788 | LTF | Lactotransferrin | 131 | 9,39E+14 | 7,17E+14 | 3,60E+14 |
| **103** | A0A7M7LSQ8 | 724341 | Endoplasmic reticulum resident protein 29 | 681 | -8,83E+14 | 7,39E+14 | 3,67E+14 |
| **104** | A0A7M7IVL6 | LOC409468 | A disintegrin and metalloproteinase with thrombospondin motifs 8 isoform X1 | 1 | -6,35E+14 | 7,55E+14 | 3,70E+14 |
| **105** | A0A7M7GZH3 | 409488 | Protein-L-isoaspartate O-methyltransferase | 1 | -6,38E+14 | 8,10E+14 | 3,83E+14 |
| **106** | A0A7M7FZE7 | LOC725041 | Leucine-rich repeat-containing protein 15 | 92 | -7,87E+14 | 8,01E+14 | 3,83E+14 |
| **107** | A0A7M6URI6 | Hsc70-3 | Heat shock protein cognate 3 precursor | 768 | -8,83E+14 | 8,04E+14 | 3,83E+14 |
| **108** | A0A7M7FZB3 | LOC726737 | Venom acid phosphatase Acph-1 | 33 | 6,65E+14 | 8,20E+14 | 3,83E+14 |
| **109** | O77727 | KRT15 | Keratin, type I cytoskeletal 15 | 27 | 1,52E+14 | 8,39E+14 | 3,86E+14 |
| **110** | A0A7M7MMB8 | LOC726872 | LDLR chaperone boca | 13 | -5,76E+14 | 8,75E+13 | 3,86E+14 |
| **111** | A0A7M7H507 | LOC552141 | Beta-1,4-N-acetylgalactosaminyltransferase | 40 | -6,34E+14 | 8,64E+14 | 3,86E+14 |
| **112** | A0A7M7GMF0 | 552756 | Cathepsin L1 | 210 | -8,06E+14 | 8,55E+14 | 3,86E+14 |
| **113** | A0A7M7R8D8 | 411647 | Calumenin | 268 | -8,21E+14 | 8,67E+14 | 3,86E+14 |
| **114** | A0A7M7LN74 | 100578232 | Notch homolog 2 N-terminal-like protein | 5 | -1,13E+14 | 9,11E+14 | 3,93E+14 |
| **115** | A0A7M7GQQ6 | LOC100578311 | UMP-CMP kinase | 1 | -6,13E+14 | 9,03E+13 | 3,93E+14 |
| **116** | A0A7M7MM69 | LOC412835 | Nucleoporin NUP35 | 76 | 8,04E+14 | 9,31E+14 | 3,97E+14 |
| **117** | A0A7M7R8L8 | LOC413614 | Ras-related protein Rab-14 | 6 | -6,68E+14 | 9,40E+14 | 3,97E+14 |
| **118** | A0A7M7R586 | 408426 | Lysosomal thioesterase PPT2 homolog isoform X1 | 2 | 7,57E+14 | 9,69E+14 | 4,05E+14 |
| **119** | A0A7M7LPI2 | LOC413441 | DnaJ homolog subfamily C member 3 | 111 | -7,86E+14 | 9,92E+14 | 4,10E+14 |
| **120** | A0A7M7LRS3 | 550798 | Protein ERGIC-53 | 143 | -7,09E+14 | 1,20E+14 | 4,39E+14 |
| **121** | A0A7M7GV42 | 408851 | Cysteine proteinase CG12163 isoform X1 | 9 | -9,01E+13 | 1,24E+14 | 4,49E+14 |
| **122** | A0A7M7H184 | LOC412536 | Leukocyte elastase inhibitor | 23 | -6,89E+14 | 1,26E+14 | 4,51E+14 |
| **123** | A0A7M7R900 | 412526 | Protein disulfide-isomerase A6 homolog | 59 | -6,31E+14 | 1,28E+14 | 4,54E+14 |
| **124** | A0A7M7GPD9 | LOC551012 | Cation-independent mannose-6-phosphate receptor isoform X1 | 1 | -1,01E+14 | 1,30E+14 | 4,56E+14 |
| **125** | A0A7M7LMV8 | 413749 | Serine protease inhibitor 88Ea isoform X1 | 1 | -6,76E+14 | 1,37E+12 | 4,79E+14 |
| **126** | A0A7M6UMR7 | Hex110 | Hexamerin 110 precursor | 5 | 6,88E+14 | 1,39E+14 | 4,79E+14 |
| **127** | P56682 |  | Chymotrypsin inhibitor | 49 | 1,36E+14 | 1,42E+12 | 4,79E+14 |
| **128** | A0A7M7L386 | LOC724816 | Carboxypeptidase D | 1 | -5,53E+14 | 1,41E+14 | 4,79E+14 |
| **129** | A0A7M7GXZ4 | LOC726552 | NEDD8 | 5 | -6,02E+14 | 1,42E+11 | 4,79E+14 |
| **130** | A0A7M7R759 | 409801 | Carboxylic ester hydrolase | 125 | 8,72E+14 | 1,47E+14 | 4,84E+14 |
| **131** | A0A7M7R2Y3 | 408621 | ER membrane protein complex subunit 7 | 57 | -6,73E+13 | 1,50E+14 | 4,84E+14 |
| **132** | A0A7M7GQS0 | 551603 | Ras-related protein Rab-11A | 58 | -5,95E+14 | 1,48E+14 | 4,84E+14 |
| **133** | A0A7M7IGH2 | LOC409368 | LOW QUALITY PROTEIN: interferon-related developmental regulator 1-like | 67 | -5,96E+14 | 1,49E+14 | 4,84E+14 |
| **134** | A0A7M7GPU6 | LOC411417 | Uncharacterized protein LOC411417 isoform X2 | 1 | -6,07E+14 | 1,50E+14 | 4,84E+14 |
| **135** | A0A7M7L6X9 | LOC726286 | Uncharacterized protein LOC726286 | 25 | -5,91E+14 | 1,56E+14 | 4,94E+14 |
| **136** | A0A7M7H280 | 408987 | Sortilin-related receptor | 43 | -5,28E+14 | 1,60E+14 | 4,99E+14 |
| **137** | A0A7M7GVH6 | 409790 | Multiple coagulation factor deficiency protein 2 homolog | 168 | -7,89E+14 | 1,60E+14 | 4,99E+14 |
| **138** | A0A7M7GTG7 | 552602 | Transmembrane emp24 domain-containing protein | 26 | -5,63E+14 | 1,65E+14 | 5,07E+14 |
| **139** | A0A7M7G005 | 726353 | Uncharacterized protein LOC726353 isoform X1 | 1 | -4,42E+14 | 1,68E+14 | 5,08E+14 |
| **140** | A0A7M7GE53 | 100577527 | Uncharacterized protein LOC100577527 | 68 | -5,88E+14 | 1,69E+13 | 5,08E+14 |
| **141** | A0A7M7R6I1 | LOC408557 | Thioredoxin domain-containing protein 5 homolog | 5 | -8,95E+14 | 1,66E+13 | 5,08E+14 |
| **142** | A0A7M7R370 | LOC409026 | Transmembrane emp24 domain-containing protein bai | 13 | -6,17E+14 | 1,72E+14 | 5,09E+14 |
| **143** | A0A7M7R9B8 | 411765 | Rab GDP dissociation inhibitor | 8 | -9,84E+14 | 1,72E+14 | 5,09E+14 |
| **144** | A0A7M6UUL3 | Obp17 | OBP17 | 29 | 8,03E+14 | 1,78E+14 | 5,12E+14 |
| **145** | A0A7M7GER9 | LOC552798 | Sodium-coupled neutral amino acid transporter 10 isoform X1 | 1 | 6,89E+14 | 1,75E+14 | 5,12E+14 |
| **146** | A0A7M7GM85 | 550671 | CLIP domain-containing serine protease | 402 | -6,37E+12 | 1,78E+14 | 5,12E+14 |
| **147** | A0A7M7GB63 | 409481 | ADP-ribosylation factor | 41 | -5,65E+14 | 1,76E+14 | 5,12E+14 |
| **148** | A0A7M7FZJ9 | LOC725926 | Collagen alpha-1(IV) chain isoform X2 | 1 | -5,35E+13 | 1,87E+13 | 5,15E+14 |
| **149** | P85831 |  | Prohormone-4 | 28 | -5,09E+14 | 1,85E+14 | 5,15E+14 |
| **150** | A0A7M7R6X2 | LOC409091 | Neurotrimin | 132 | -5,60E+14 | 1,89E+14 | 5,15E+14 |
| **151** | A0A7M6UVZ7 | 727472 | Peptidoglycan-recognition protein | 64 | -5,66E+14 | 1,83E+14 | 5,15E+14 |
| **152** | A0A7M7MQZ9 | LOC409474 | Laminin subunit gamma-1 | 393 | -6,31E+14 | 1,89E+14 | 5,15E+14 |
| **153** | A0A7M7G3D2 | 727309 | Glutaredoxin-C4 isoform X2 | 17 | -6,72E+14 | 1,80E+14 | 5,15E+14 |
| **154** | A0A7M7FYF7 | LOC725287 | Sulfhydryl oxidase | 27 | -7,30E+14 | 1,87E+13 | 5,15E+14 |
| **155** | A0A7M6W876 | LOC406114 | Alpha-amylase | 18 | -9,23E+14 | 1,84E+14 | 5,15E+14 |
| **156** | A0A7M7GUT3 | LOC503505 | Icarapin-like isoform X1 | 141 | -5,58E+13 | 1,91E+14 | 5,15E+14 |
| **157** | P35527 | KRT9 | Keratin, type I cytoskeletal 9 | 374 | 1,89E+14 | 1,94E+14 | 5,17E+14 |
| **158** | P04264 | KRT1 | Keratin, type II cytoskeletal 1 | 518 | 1,79E+14 | 1,94E+14 | 5,17E+14 |
| **159** | A0A7M7GNS0 | 409004 | Ras-related protein Rab6 isoform X1 | 1 | -4,61E+13 | 1,95E+14 | 5,17E+14 |
| **160** | A0A7M7GQB8 | LOC552636 | Dipeptidase | 1 | -8,20E+13 | 1,99E+14 | 5,19E+14 |
| **161** | A0A7M6UGK8 | Rpn1 | Dolichyl-diphosphooligosaccharide--protein glycosyltransferase subunit 1 | 115 | -5,37E+14 | 1,98E+14 | 5,19E+14 |
| **162** | A0A7M7IRS5 | LOC107963996 | Leucine-rich repeat-containing protein 70 | 49 | 6,04E+14 | 2,20E+14 | 5,42E+14 |
| **163** | A0A7M7M4R3 | LOC413324 | Chitinase-like protein Idgf4 isoform X2 | 26 | 4,47E+14 | 2,24E+14 | 5,42E+14 |
| **164** | A0A7M7IRY4 | 410769 | Aminopeptidase | 1 | -1,01E+14 | 2,23E+14 | 5,42E+14 |
| **165** | A0A7M7R866 | 552091 | Group XV phospholipase A2 isoform X1 | 26 | -4,41E+14 | 2,18E+14 | 5,42E+14 |
| **166** | A0A7M7GKS8 | LOC411889 | Serine protease K12H4.7 | 86 | -5,67E+14 | 2,24E+14 | 5,42E+14 |
| **167** | A0A7M7GSJ6 | 414035 | Lachesin isoform X2 | 1 | -8,23E+14 | 2,23E+14 | 5,42E+14 |
| **168** | A0A7M7H478 | 725718 | Cofilin/actin-depolymerizing factor homolog | 76 | -5,77E+14 | 2,35E+14 | 5,59E+14 |
| **169** | A0A7M7R608 | 412150 | Heat shock protein 83 | 325 | -6,02E+14 | 2,36E+14 | 5,59E+14 |
| **170** | A0A7M7TFY7 | 552747 | Glucosidase 2 subunit beta | 141 | -5,47E+14 | 2,38E+14 | 5,59E+14 |
| **171** | A0A7M7L3J3 | LOC413785 | peptidylprolyl isomerase | 1 | -3,93E+14 | 2,41E+14 | 5,63E+14 |
| **172** | A0A7M7GAF4 | 100576444 | Chymotrypsin inhibitor | 246 | -8,91E+14 | 2,42E+14 | 5,63E+14 |
| **173** | A0A7M7L992 | LOC100577764 | small monomeric GTPase | 4 | -4,22E+14 | 2,45E+14 | 5,65E+14 |
| **174** | A0A7M7LKE8 | LOC724436 | phospholipase A2 | 545 | 6,26E+14 | 2,47E+14 | 5,66E+14 |
| **175** | A0A7M7RB56 | 552645 | Mesencephalic astrocyte-derived neurotrophic factor homolog | 32 | -4,41E+14 | 2,50E+14 | 5,66E+14 |
| **176** | A0A7M7IFF9 | 409167 | Translation elongation factor 2 isoform X1 | 24 | -5,70E+14 | 2,51E+14 | 5,66E+14 |
| **177** | A0A7M7GTZ8 | LOC102655329 | 14-3-3 protein zeta isoform X1 | 1 | -8,46E+14 | 2,49E+14 | 5,66E+14 |
| **178** | A0A087ZYQ1 | LOC411014 | Malate dehydrogenase | 17 | -6,56E+14 | 2,53E+14 | 5,68E+14 |
| **179** | A0A7M7KZK9 | LOC551170 | Uncharacterized protein LOC551170 isoform X3 | 1 | -5,49E+14 | 2,55E+14 | 5,70E+14 |
| **180** | A0A7M7GEI7 | LOC100578816 | Uncharacterized protein LOC100578816 | 19 | 6,04E+14 | 2,63E+14 | 5,75E+14 |
| **181** | A0A7M7MTL0 | CUTA | Protein CutA homolog isoform X1 | 18 | -3,87E+13 | 2,62E+14 | 5,75E+14 |
| **182** | A0A7M7MQD1 | LOC410337 | Venom dipeptidyl peptidase 4 isoform X1 | 404 | -5,43E+14 | 2,63E+14 | 5,75E+14 |
| **183** | A0A7M7GNJ2 | LOC552018 | tRNA (guanine(26)-N(2))-dimethyltransferase | 26 | -6,02E+14 | 2,63E+14 | 5,75E+14 |
| **184** | Q06830 | PRDX1 | Peroxiredoxin-1 | 37 | 4,27E+14 | 6,24E+14 | 5,84E+14 |
| **185** | A0A7M7GSP5 | LOC100577777 | Cysteine-rich venom protein 6 isoform X2 | 1 | 1,59E+14 | 7,35E+14 | 5,84E+14 |
| **186** | Q17058 |  | Alpha-glucosidase | 111 | -6,00E+14 | 2,71E+14 | 5,89E+14 |
| **187** | A0A7M7MGK9 | LOC552249 | Alpha-mannosidase | 1 | 4,99E+14 | 2,77E+14 | 5,98E+14 |
| **188** | A0A7M7R9P8 | 551250 | Microsomal triglyceride transfer protein large subunit | 16 | 5,24E+14 | 2,82E+14 | 6,00E+14 |
| **189** | A0A7M7GQU7 | LOC412838 | Alpha-mannosidase | 16 | -3,88E+14 | 2,81E+14 | 6,00E+14 |
| **190** | A0A7M7GYE7 | LOC412273 | Probable chitinase 10 isoform X2 | 1 | -5,05E+14 | 2,83E+14 | 6,00E+14 |
| **191** | A0A7M7MM50 | LOC409814 | Beta-glucuronidase | 106 | -5,21E+14 | 2,91E+14 | 6,15E+14 |
| **192** | A0A7M7GFE2 | LOC100577168 | Uncharacterized protein LOC100577168 | 37 | 4,22E+14 | 2,94E+14 | 6,16E+14 |
| **193** | A0A7M7G0D7 | 727126 | Uncharacterized protein LOC727126 | 37 | -5,84E+14 | 2,95E+14 | 6,16E+14 |
| **194** | A0A7M7R7L3 | 412543 | Uncharacterized protein LOC412543 isoform X1 | 14 | -3,73E+14 | 3,00E+14 | 6,20E+14 |
| **195** | A0A7M7IJY0 | LOC409714 | protein-tyrosine-phosphatase | 1 | -5,34E+14 | 2,99E+14 | 6,20E+14 |
| **196** | Q86PH6 | Tsf1 | Transferrin | 928 | 5,39E+14 | 3,03E+14 | 6,23E+14 |
| **197** | A0A7M7R7C9 | LOC410035 | Dorsal-ventral patterning protein Sog isoform X2 | 7 | -3,13E+14 | 3,07E+14 | 6,29E+14 |
| **198** | A0A7M7LT84 | 100578782 | Complex III subunit 9 | 29 | 3,97E+14 | 3,16E+14 | 6,38E+14 |
| **199** | A0A7M7GZX8 | LOC725078 | Uncharacterized protein LOC725078 isoform X1 | 1 | 3,34E+14 | 3,18E+14 | 6,38E+14 |
| **200** | A0A7M7R7N1 | 411799 | Polypeptide N-acetylgalactosaminyltransferase | 3 | -6,53E+14 | 3,14E+14 | 6,38E+14 |
| **201** | A0A7M7MP33 | LOC413792 | Nucleoprotein TPR isoform X1 | 1 | -7,38E+14 | 3,18E+14 | 6,38E+14 |
| **202** | A0A7M7L8V2 | LOC725169 | Succinate--CoA ligase [GDP-forming] subunit beta, mitochondrial | 41 | -5,08E+13 | 3,20E+14 | 6,39E+14 |
| **203** | A0A7M7R8E8 | 411622 | Uncharacterized protein LOC411622 | 6 | -2,35E+13 | 1,68E+14 | 6,67E+14 |
| **204** | A0A7M7R5W7 | 411943 | glutaminyl-peptide cyclotransferase | 10 | -1,49E+14 | 1,39E+14 | 6,67E+14 |
| **205** | A0A7M7RAC1 | 551350 | Myotrophin | 14 | -1,49E+14 | 1,31E+14 | 6,67E+14 |
| **206** | A0A7M7R6U6 | LOC410975 | Uncharacterized protein LOC410975 isoform X2 | 17 | -1,51E+14 | 1,64E+14 | 6,67E+14 |
| **207** | A0A7M7LP09 | 410380 | Neprilysin-2 isoform X2 | 1 | -1,90E+14 | 1,46E+14 | 6,67E+14 |
| **208** | A0A7M7RB96 | 551968 | Aldose reductase | 35 | 3,68E+14 | 3,38E+14 | 6,71E+14 |
| **209** | A0A7M7MMK3 | LOC113218958 | Superoxide dismutase [Cu-Zn] | 41 | 5,11E+13 | 3,46E+14 | 6,83E+14 |
| **210** | A0A7M6UME9 | 406154 | Profilin | 9 | -2,61E+14 | 3,50E+14 | 6,84E+14 |
| **211** | A0A7M7ISB8 | 552086 | Aldose 1-epimerase | 1 | -2,88E+14 | 3,49E+14 | 6,84E+14 |
| **212** | A0A7M7MR18 | 551268 | phospholipase A1 | 18 | -1,24E+13 | 2,07E+14 | 6,87E+14 |
| **213** | A0A7M6UQF8 | Obp4 | Odorant binding protein 4 precursor | 60 | -1,44E+14 | 2,08E+14 | 6,87E+14 |
| **214** | A0A7M7MPW6 | 551793 | Uncharacterized protein LOC551793 isoform X1 | 1 | -4,00E+14 | 3,64E+14 | 7,05E+14 |
| **215** | A0A7M7RA20 | LOC408807 | IRP30 | 53 | 6,98E+14 | 3,67E+14 | 7,06E+14 |
| **216** | A0A7M7MNC0 | LOC726444 | Ectonucleoside triphosphate diphosphohydrolase 5 | 132 | -4,26E+14 | 3,68E+14 | 7,06E+14 |
| **217** | B2D0J4 |  | Venom dipeptidyl peptidase 4 | 41 | 5,54E+14 | 3,75E+14 | 7,09E+14 |
| **218** | A0A8U0WQC3 | Obp14 | Odorant binding protein 14 precursor | 306 | 5,11E+14 | 3,74E+14 | 7,09E+14 |
| **219** | A0A7M7IGK1 | LOC725122 | Uncharacterized protein LOC725122 isoform X2 | 33 | -4,43E+14 | 3,73E+14 | 7,09E+14 |
| **220** | Q08169 |  | Hyaluronidase | 610 | -4,07E+14 | 3,78E+13 | 7,11E+14 |
| **221** | A0A7M7R3H9 | LOC409278 | Apolipoprotein D | 21 | 4,41E+14 | 4,01E+14 | 7,23E+14 |
| **222** | O18330 | MRJP1 | Major royal jelly protein 1 | 817 | 4,39E+14 | 3,95E+14 | 7,23E+14 |
| **223** | Q17060 | MRJP3 | Major royal jelly protein 3 | 374 | 4,31E+14 | 3,99E+14 | 7,23E+14 |
| **224** | A0A7M7GNU1 | LOC725883 | Guanine nucleotide-binding protein subunit gamma-1 | 25 | 3,57E+14 | 3,97E+14 | 7,23E+14 |
| **225** | A0A7M7SS83 | LOC552435 | alpha-1,2-Mannosidase | 43 | -3,39E+13 | 3,99E+14 | 7,23E+14 |
| **226** | Q9U8X6 | LOC406081 | Glucose oxidase | 96 | -4,93E+13 | 4,06E+14 | 7,23E+14 |
| **227** | A0A7M6UDS2 | 409398 | Superoxide dismutase [Cu-Zn] | 76 | -3,59E+14 | 4,03E+14 | 7,23E+14 |
| **228** | A0A7M7LIA2 | 724382 | Protein obstructor-E | 12 | -3,74E+14 | 3,98E+13 | 7,23E+14 |
| **229** | A0A7M7TFR2 | LOC550884 | N-sulphoglucosamine sulphohydrolase | 133 | -3,77E+14 | 4,04E+14 | 7,23E+14 |
| **230** | A0A7M7MR23 | LOC727193 | phospholipase A1 | 33 | -4,75E+14 | 4,05E+14 | 7,23E+14 |
| **231** | A0A7M7H042 | LOC725552 | DNA-directed RNA polymerase subunit | 1 | -4,76E+14 | 3,90E+14 | 7,23E+14 |
| **232** | A0A7M7LM75 | LOC409826 | Proteoglycan 4 isoform X2 | 1 | -6,07E+14 | 4,01E+14 | 7,23E+14 |
| **233** | A0A7M7GQ25 | LOC409517 | Digestive cysteine proteinase 1 | 278 | -3,84E+14 | 4,09E+14 | 7,25E+14 |
| **234** | A0A7M7M5A5 | 107965322 | Gamma-interferon-inducible lysosomal thiol reductase | 100 | -4,69E+14 | 4,11E+14 | 7,25E+14 |
| **235** | A0A7M7TFR9 | 551785 | 6-phosphogluconolactonase | 30 | -4,19E+14 | 4,16E+14 | 7,32E+14 |
| **236** | P00630 |  | Phospholipase A2 | 8768 | 2,64E+14 | 4,23E+14 | 7,33E+14 |
| **237** | Q10416 |  | Hymenoptaecin | 19 | -3,61E+14 | 4,21E+14 | 7,33E+14 |
| **238** | A0A7M7R506 | LOC410734 | Glucose dehydrogenase [FAD, quinone] | 201 | -3,70E+14 | 4,20E+14 | 7,33E+14 |
| **239** | P35908 | KRT2 | Keratin, type II cytoskeletal 2 epidermal | 183 | 8,45E+14 | 4,34E+14 | 7,41E+14 |
| **240** | O77061 | MRJP2 | Major royal jelly protein 2 | 27 | 3,34E+14 | 4,36E+14 | 7,41E+14 |
| **241** | A0A7M7FYN8 | LOC725756 | Beta-galactosidase | 451 | -3,59E+13 | 4,37E+14 | 7,41E+14 |
| **242** | A0A7M7SQD3 | LOC551435 | Protein disulfide-isomerase | 577 | -3,73E+14 | 4,40E+13 | 7,44E+14 |
| **243** | A0A7M7H6B6 | 727510 | Lactosylceramide 4-alpha-galactosyltransferase isoform X2 | 1 | -2,54E+14 | 4,42E+14 | 7,44E+14 |
| **244** | A0A7M7GMY7 | 408951 | 14-3-3 protein epsilon | 8 | -5,05E+14 | 4,48E+14 | 7,50E+14 |
| **245** | A0A7M7SQ18 | LOC408961 | Apolipophorins | 2185 | 3,69E+14 | 4,51E+14 | 7,52E+14 |
| **246** | A0A7M7L6C1 | LOC100577054 | Uncharacterized protein LOC100577054 isoform X1 | 2 | -2,16E+14 | 4,55E+13 | 7,56E+14 |
| **247** | A0A7M7R6H0 | 408851 | Cysteine proteinase CG12163 isoform X2 | 114 | -3,04E+14 | 4,70E+13 | 7,66E+14 |
| **248** | A0A7M7L4E2 | LOC408826 | Dystroglycan 1 | 1 | -3,50E+14 | 4,71E+14 | 7,66E+14 |
| **249** | A0A7M7GRE8 | LOC413987 | Polypeptide N-acetylgalactosaminyltransferase | 4 | -3,02E+14 | 4,73E+12 | 7,67E+14 |
| **250** | A0A7M7MVS7 | LOC412513 | Uncharacterized protein LOC412513 | 131 | -2,87E+13 | 4,94E+14 | 7,75E+14 |
| **251** | A0A7M7H6R7 | 102654353 | Fucosyltransferase | 95 | -2,95E+14 | 4,88E+14 | 7,75E+14 |
| **252** | A0A7M7R9F4 | 552600 | GILT-like protein 1 | 80 | -3,04E+14 | 4,91E+14 | 7,75E+14 |
| **253** | A0A7M7FZE8 | 725260 | Uncharacterized protein LOC725260 | 41 | -3,13E+14 | 4,83E+14 | 7,75E+14 |
| **254** | A0A7M7MSF1 | LOC551913 | Protein CREG1 | 229 | -3,29E+14 | 4,81E+14 | 7,75E+14 |
| **255** | A0A7M7GXL7 | LOC552223 | DnaJ homolog shv | 478 | -3,34E+14 | 4,93E+14 | 7,75E+14 |
| **256** | A0A7M7MNA5 | LOC409281 | Protein sel-1 homolog 1 | 43 | -3,59E+14 | 4,84E+14 | 7,75E+14 |
| **257** | H9KQJ7 |  | Omega-conotoxin-like protein 1 | 135 | -4,82E+14 | 4,93E+13 | 7,75E+14 |
| **258** | A0A7M7LQ41 | LOC412045 | Dolichyl-diphosphooligosaccharide--protein glycosyltransferase subunit 2 | 113 | -2,90E+14 | 5,01E+12 | 7,79E+14 |
| **259** | A0A7M6UVG4 | Cpap3-b | Cuticular protein analogous to peritrophins 3-B precursor | 148 | -3,02E+14 | 5,02E+14 | 7,79E+14 |
| **260** | A0A7M7GTC0 | 412808 | Aminopeptidase | 1 | 4,35E+14 | 5,43E+14 | 7,93E+14 |
| **261** | A0A7M7IGY7 | 724779 | Protein artichoke | 104 | 3,09E+14 | 5,36E+14 | 7,93E+14 |
| **262** | A0A7M7R9K2 | 552722 | Glyoxalase domain-containing protein 4 | 24 | 5,16E+13 | 5,31E+14 | 7,93E+14 |
| **263** | A0A7M7GYC6 | 724900 | Uncharacterized protein LOC724900 isoform X3 | 1 | -4,93E+12 | 5,37E+14 | 7,93E+14 |
| **264** | Q86MV4 | PPO | Phenoloxidase subunit A3 | 152 | -3,08E+13 | 5,18E+14 | 7,93E+14 |
| **265** | A0A7M7MMZ2 | LOC411344 | Fasciclin-1 isoform X4 | 1 | -2,00E+14 | 5,45E+14 | 7,93E+14 |
| **266** | A0A088AQF2 | 107965838 | Endoribonuclease | 34 | -2,42E+14 | 5,45E+14 | 7,93E+14 |
| **267** | A0A7M7R7J6 | LOC409861 | Nucleoside diphosphate kinase | 66 | -2,47E+14 | 5,30E+13 | 7,93E+14 |
| **268** | A0A7M7GPV3 | LOC551882 | Protein dj-1beta | 48 | -2,54E+14 | 5,37E+12 | 7,93E+14 |
| **269** | A0A7M7MTB6 | 406146 | Hyaluronidase | 133 | -2,56E+14 | 5,42E+14 | 7,93E+14 |
| **270** | A0A7M7G858 | LOC725661 | Uncharacterized protein LOC725661 | 127 | -2,59E+14 | 5,27E+13 | 7,93E+14 |
| **271** | A0A7M7H421 | LOC725899 | Alpha-galactosidase | 25 | -3,00E+14 | 5,32E+14 | 7,93E+14 |
| **272** | Q6W3E3 | Mrjp6 | Bee-milk protein | 33 | -3,43E+14 | 5,21E+14 | 7,93E+14 |
| **273** | A0A7M7IGQ7 | LOC100576326 | Phenoloxidase-activating factor 2 isoform X1 | 1 | -5,14E+14 | 5,41E+14 | 7,93E+14 |
| **274** | A0A7M7FXY2 | LOC725038 | NPC intracellular cholesterol transporter 2 homolog a | 178 | -5,29E+14 | 5,31E+14 | 7,93E+14 |
| **275** | A0A7M7SQT4 | LOC100578744 | Uncharacterized protein LOC100578744 | 158 | 2,79E+14 | 5,51E+14 | 7,96E+14 |
| **276** | A0A7M7R3V2 | LOC409663 | lysozyme | 121 | -2,42E+14 | 5,51E+13 | 7,96E+14 |
| **277** | A0A7M7L3I9 | LOC724811 | kynurenine--oxoglutarate transaminase | 144 | -2,72E+14 | 5,54E+14 | 7,97E+14 |
| **278** | A0A7M7R7Z9 | 551684 | Ferritin | 152 | 2,40E+14 | 5,69E+13 | 8,09E+14 |
| **279** | A0A7M7ISX2 | LOC412198 | UDP-glucose:glycoprotein glucosyltransferase isoform X1 | 14 | -1,97E+14 | 5,66E+14 | 8,09E+14 |
| **280** | A0A7M7GM92 | LOC411748 | LOW QUALITY PROTEIN: protein kinase C-binding protein NELL1 | 74 | -2,46E+14 | 5,68E+14 | 8,09E+14 |
| **281** | A0A7M7MQS4 | LOC100578030 | Antichymotrypsin-2 isoform X1 | 1 | -2,30E+14 | 5,72E+14 | 8,10E+14 |
| **282** | A0A7M7GS92 | 100578168 | Fucosyltransferase | 1 | -1,88E+14 | 5,88E+14 | 8,23E+14 |
| **283** | A0A7M6UCT9 | Hsc70-4 | Heat shock protein cognate 4 | 86 | -2,38E+14 | 5,88E+14 | 8,23E+14 |
| **284** | A0A7M7R7K3 | 551273 | Lysosomal Pro-X carboxypeptidase | 312 | -2,55E+14 | 5,88E+14 | 8,23E+14 |
| **285** | A0A7M7RBC3 | LOC552734 | Endoribonuclease | 17 | -4,17E+14 | 5,85E+14 | 8,23E+14 |
| **286** | A0A7M7R3G4 | 409265 | Dolichyl-diphosphooligosaccharide--protein glycosyltransferase subunit STT3A | 15 | 2,32E+14 | 6,01E+14 | 8,31E+14 |
| **287** | A0A7M7GUN4 | LOC411006 | Endoplasmic reticulum lectin 1 | 1 | -1,68E+13 | 6,03E+14 | 8,31E+14 |
| **288** | A0A7M7GRI9 | 409849 | Fasciclin-2 isoform X3 | 1 | -1,60E+14 | 6,02E+14 | 8,31E+14 |
| **289** | A0A7M7R8L1 | 411890 | Nucleolysin TIAR | 4 | -3,23E+14 | 5,98E+14 | 8,31E+14 |
| **290** | A0A7M6UMJ2 | Obp13 | OBP13 | 75 | -2,22E+14 | 6,09E+14 | 8,34E+14 |
| **291** | A0A7M7IJ14 | LOC726352 | Transmembrane protease serine 11B-like protein | 23 | -4,05E+14 | 6,09E+14 | 8,34E+14 |
| **292** | A0A7M7MU23 | LOC551651 | Ferritin | 305 | 2,27E+14 | 6,12E+14 | 8,34E+14 |
| **293** | A0A7M7GQ39 | LOC408937 | SPARC | 51 | -2,63E+14 | 6,13E+14 | 8,34E+14 |
| **294** | A0A7M7M5C7 | 410451 | Carboxypeptidase | 124 | -2,21E+14 | 6,18E+14 | 8,37E+14 |
| **295** | A0A7M7IMS8 | LOC551688 | Band 7 protein AGAP004871 isoform X4 | 1 | 3,35E+14 | 6,39E+14 | 8,39E+14 |
| **296** | A0A7M7R2L6 | 408572 | Transgelin | 21 | 2,21E+14 | 6,36E+13 | 8,39E+14 |
| **297** | A0A7M7KYY1 | LOC725591 | Protein crumbs isoform X2 | 1 | 1,94E+14 | 6,46E+13 | 8,39E+14 |
| **298** | A0A7M7L949 | LOC413012 | EH domain-containing protein 3 | 21 | 1,72E+14 | 6,39E+14 | 8,39E+14 |
| **299** | A0A7M7ILH0 | LOC410362 | MOXD1 homolog 2 | 214 | -2,18E+13 | 6,40E+14 | 8,39E+14 |
| **300** | A0A7M7MN70 | LOC408618 | Furin-like protease 2 isoform X1 | 2 | -1,42E+14 | 6,31E+14 | 8,39E+14 |
| **301** | A0A7M7L2X7 | LOC412103 | Transport and Golgi organization protein 1 isoform X4 | 1 | -1,49E+14 | 6,37E+14 | 8,39E+14 |
| **302** | A0A087ZYX8 | LOC409143 | Venom serine protease 34 isoform X1 | 1 | -1,66E+14 | 6,43E+14 | 8,39E+14 |
| **303** | A0A7M7GY33 | 725882 | Uncharacterized protein LOC725882 | 35 | -1,80E+14 | 6,27E+14 | 8,39E+14 |
| **304** | A0A7M7MTF8 | LOC413569 | protein-tyrosine-phosphatase | 1 | -2,12E+14 | 6,47E+14 | 8,39E+14 |
| **305** | A0A7M7GMA7 | 408412 | Prohibitin | 9 | -3,16E+14 | 6,49E+14 | 8,39E+14 |
| **306** | P62979 | RPS27A | Ubiquitin-40S ribosomal protein S27a | 1 | -3,45E+14 | 6,27E+13 | 8,39E+14 |
| **307** | A0A7M7GAL3 | 409451 | Thioredoxin | 28 | -3,78E+14 | 6,42E+14 | 8,39E+14 |
| **308** | A0A7M7H012 | LOC724993 | Uncharacterized protein LOC724993 | 18 | -4,02E+14 | 6,39E+14 | 8,39E+14 |
| **309** | A4ZXC4 | Tpi | Triosephosphate isomerase | 69 | -1,68E+14 | 6,57E+14 | 8,43E+14 |
| **310** | A0A7M7H1B6 | 551749 | LARGE xylosyl- and glucuronyltransferase 1 | 17 | -1,96E+14 | 6,56E+14 | 8,43E+14 |
| **311** | A0A7M7R5S0 | LOC409365 | Lysosomal alpha-glucosidase | 39 | -2,13E+14 | 6,59E+14 | 8,43E+14 |
| **312** | A0A7M7H423 | LOC552459 | Glycine-rich cell wall structural protein 1 isoform X2 | 1 | 3,56E+14 | 6,93E+14 | 8,44E+14 |
| **313** | A0A7M7GQX8 | 408808 | Prosaposin isoform X2 | 16 | 3,16E+14 | 6,67E+14 | 8,44E+14 |
| **314** | A0A7M7MSB6 | LOC410369 | Uncharacterized protein LOC410369 | 13 | 3,10E+14 | 6,73E+14 | 8,44E+14 |
| **315** | A0A7M7IM62 | 552051 | Dolichyl-diphosphooligosaccharide--protein glycosyltransferase 48 kDa subunit | 7 | 2,56E+14 | 6,86E+14 | 8,44E+14 |
| **316** | Q25BT7 | Hbg2 | alpha-glucosidase | 174 | 2,11E+14 | 6,81E+14 | 8,44E+14 |
| **317** | O97432 | MRJP5 | Major royal jelly protein 5 | 117 | 2,05E+14 | 6,91E+14 | 8,44E+14 |
| **318** | A0A7M6W5F9 | MRJP7 | Bee-milk protein | 115 | 1,84E+14 | 6,73E+14 | 8,44E+14 |
| **319** | A0A7M7GL03 | LOC726966 | Angiotensin-converting enzyme | 11 | 1,80E+14 | 6,98E+14 | 8,44E+14 |
| **320** | A0A7M7GRZ5 | 409668 | Proteasome inhibitor PI31 subunit | 1 | 1,55E+14 | 6,97E+14 | 8,44E+14 |
| **321** | A0A7M7SRI2 | LOC552678 | Enolase | 71 | 1,51E+14 | 6,93E+14 | 8,44E+14 |
| **322** | A0A7M6UW32 | 406122 | Actin related protein 1 | 51 | -2,09E+13 | 6,86E+14 | 8,44E+14 |
| **323** | A0A7M7L4T5 | LOC411978 | Myrosinase 1 | 8 | -3,13E+13 | 6,76E+14 | 8,44E+14 |
| **324** | B2D0J5 |  | Venom carboxylesterase-6 | 2426 | -1,66E+14 | 6,80E+14 | 8,44E+14 |
| **325** | A0A7M7L1T2 | LOC552572 | Eukaryotic translation initiation factor 5A | 23 | -1,70E+14 | 6,65E+14 | 8,44E+14 |
| **326** | A0A7M7MQW1 | LOC552683 | Chitinase domain-containing protein 1 | 146 | -1,73E+14 | 6,85E+14 | 8,44E+14 |
| **327** | B8Y9E0 | C1q-VP | C1q-like venom protein | 190 | -1,81E+14 | 6,89E+14 | 8,44E+14 |
| **328** | A0A7M7MKK8 | LOC408874 | Uncharacterized protein LOC408874 isoform X9 | 413 | -1,87E+14 | 6,88E+14 | 8,44E+14 |
| **329** | A0A7M7H331 | LOC725848 | Uncharacterized protein LOC725848 | 15 | -1,95E+14 | 6,67E+14 | 8,44E+14 |
| **330** | A0A7M7TEW6 | 408782 | Tubulin beta chain | 3 | -1,38E+14 | 7,02E+14 | 8,47E+14 |
| **331** | A0A7M7LIH2 | 726495 | Protein D2 | 10 | -1,40E+14 | 7,11E+14 | 8,52E+14 |
| **332** | A0A7M7GPB8 | LOC726421 | Membrane metallo-endopeptidase-like 1 isoform X2 | 1 | 1,17E+14 | 7,16E+14 | 8,56E+14 |
| **333** | A0A7M7FZ28 | LOC724644 | Uncharacterized protein LOC724644 | 244 | -2,74E+14 | 7,21E+14 | 8,59E+14 |
| **334** | I1VC85 |  | Secapin-2 | 1 | 2,50E+14 | 7,29E+14 | 8,63E+14 |
| **335** | A0A7M7R5K9 | LOC409160 | Dihydropteridine reductase | 5 | -2,23E+14 | 7,27E+13 | 8,63E+14 |
| **336** | A0A7M7IEU7 | LOC411903 | Dyslexia-associated protein KIAA0319 | 16 | -2,14E+14 | 7,43E+14 | 8,78E+14 |
| **337** | A0A7M7IKX6 | LOC410801 | Cathepsin L1 | 178 | -1,60E+14 | 7,49E+14 | 8,83E+14 |
| **338** | Q17061 | MRJP4 | Major royal jelly protein 4 | 50 | -1,27E+14 | 7,53E+14 | 8,83E+14 |
| **339** | R4TRB9 | LOC113218576 | LYS1 | 475 | -1,51E+14 | 7,54E+14 | 8,83E+14 |
| **340** | A0A7M7GTW3 | LOC102655078 | LOW QUALITY PROTEIN: zonadhesin | 9 | -2,04E+14 | 7,61E+14 | 8,89E+14 |
| **341** | A0A7M7IXJ7 | 551205 | Glucosidase II subunit alpha | 1 | -9,89E+14 | 7,64E+14 | 8,90E+14 |
| **342** | A0A7M7RAL9 | 551381 | Uncharacterized protein LOC551381 | 19 | 1,63E+14 | 7,81E+14 | 9,02E+14 |
| **343** | A0A7M7G1N6 | 724386 | NPC intracellular cholesterol transporter 2 | 46 | 1,46E+14 | 7,86E+14 | 9,02E+14 |
| **344** | B0LUE8 | A4 | Apolipophorin-III-like protein precursor | 107 | 1,36E+14 | 7,80E+14 | 9,02E+14 |
| **345** | A0A7M7IRR6 | LOC413324 | Chitinase-like protein EN03 isoform X1 | 8 | 2,03E+13 | 7,86E+14 | 9,02E+14 |
| **346** | A0A7M7MSM4 | LOC409257 | Uncharacterized protein LOC409257 isoform X1 | 1 | -1,10E+13 | 7,88E+14 | 9,02E+14 |
| **347** | A0A7M7GSN5 | 102654987 | Ras-related protein Rab-1A | 24 | -1,37E+14 | 7,83E+14 | 9,02E+14 |
| **348** | A0A7M7INZ2 | 412523 | Out at first protein | 29 | 1,28E+14 | 7,93E+13 | 9,04E+14 |
| **349** | A0A7M7R6A3 | LOC409945 | Aspartate--tRNA ligase, cytoplasmic | 5 | -1,64E+14 | 7,97E+14 | 9,06E+14 |
| **350** | H9KL77 | 725230 | Histone H4 | 20 | 1,80E+14 | 8,19E+14 | 9,08E+14 |
| **351** | A0A7M6UBW1 | Obp18 | OBP18 | 26 | 1,06E+14 | 8,18E+13 | 9,08E+14 |
| **352** | P83563 |  | Allergen Api m 6.03 / Api m 6.04 | 939 | 1,06E+12 | 8,02E+14 | 9,08E+14 |
| **353** | A0A7M7TF36 | 410539 | Protein 5NUC | 184 | -1,08E+14 | 8,13E+14 | 9,08E+14 |
| **354** | A0A7M7L1N9 | 726269 | Glutathione peroxidase | 278 | -1,09E+14 | 8,12E+13 | 9,08E+14 |
| **355** | A0A7M7R8U4 | LOC412898 | peptidylglycine monooxygenase | 606 | -1,11E+14 | 8,06E+14 | 9,08E+14 |
| **356** | A0A7M7IHP6 | 410234 | UDP-glucuronate decarboxylase | 62 | -1,21E+14 | 8,14E+14 | 9,08E+14 |
| **357** | Q1W647 | Obp3 | Odorant binding protein 3 isoform X1 | 9 | -1,76E+14 | 8,09E+14 | 9,08E+14 |
| **358** | A0A7M7IT99 | LOC410890 | Protein seele | 41 | -1,99E+14 | 8,16E+14 | 9,08E+14 |
| **359** | A0A7M7R7F0 | 413977 | Calsyntenin-1 | 142 | 1,00E+14 | 8,22E+14 | 9,09E+14 |
| **360** | Q8I6X7 | CSP3 | Antennal-specific protein 3c | 36 | -8,46E+14 | 8,27E+14 | 9,12E+14 |
| **361** | P17722 |  | Defensin-1 | 15 | -1,07E+14 | 8,32E+14 | 9,14E+14 |
| **362** | A0A7M7MVN9 | LOC409366 | Integral membrane protein 2 | 25 | -1,38E+14 | 8,33E+13 | 9,14E+14 |
| **363** | A0A7M7IKU1 | LOC726698 | Serine protease snake | 73 | 1,24E+14 | 8,36E+14 | 9,14E+14 |
| **364** | A0A7M7G8Z8 | LOC409954 | thioredoxin-dependent peroxiredoxin | 22 | -9,21E+14 | 8,47E+14 | 9,19E+14 |
| **365** | A0A7M7TFW2 | LOC552428 | Uncharacterized protein LOC552428 | 45 | 7,15E+14 | 8,52E+14 | 9,21E+14 |
| **366** | P01501 | MELT | Melittin | 4435 | -7,03E+13 | 8,55E+14 | 9,21E+14 |
| **367** | A0A7M7LQ38 | 551227 | Myb-like protein I isoform X2 | 1 | -1,31E+14 | 8,56E+14 | 9,21E+14 |
| **368** | A0A7M7R8E3 | 411576 | Phosphoglycerate kinase | 37 | 8,57E+14 | 8,78E+14 | 9,29E+14 |
| **369** | A0A7M7G8H6 | LOC726320 | Serine protease inhibitor 27A isoform X1 | 1 | -5,36E+14 | 8,77E+13 | 9,29E+14 |
| **370** | A0A7M7M3X6 | 408666 | Uncharacterized protein LOC408666 | 1614 | -8,12E+14 | 8,78E+11 | 9,29E+14 |
| **371** | A0A7M7FZV4 | 725931 | Multiple inositol polyphosphate phosphatase 1 | 17 | -5,45E+14 | 8,87E+14 | 9,36E+14 |
| **372** | A0A7M7IGX3 | LOC410986 | Tolloid-like protein 1 isoform X5 | 1 | 4,29E+14 | 8,94E+14 | 9,42E+14 |
| **373** | A0A7M7IQ52 | 551268 | phospholipase A1 | 42 | -4,71E+14 | 8,98E+14 | 9,43E+14 |
| **374** | A0A7M7MYD9 | LOC113219418 | Uncharacterized protein LOC113219418 | 13 | -5,90E+14 | 9,01E+14 | 9,44E+14 |
| **375** | A0A7M7GC96 | LOC410733 | Glucose dehydrogenase [FAD, quinone] | 225 | 5,43E+14 | 9,15E+14 | 9,53E+14 |
| **376** | A0A7M7R743 | 409341 | Lysosomal aspartic protease | 273 | 4,90E+14 | 9,14E+14 | 9,53E+14 |
| **377** | A0A7M7MVC3 | LOC724421 | Fibrillin-2 | 66 | 1,00E+14 | 9,20E+14 | 9,56E+14 |
| **378** | A0A7M7SRZ1 | LOC113219268 | Uncharacterized protein LOC113219268 isoform X1 | 1 | -4,11E+13 | 9,25E+14 | 9,57E+14 |
| **379** | A0A7M7M5Q4 | LOC100577163 | Insulin-like growth factor-binding protein complex acid labile subunit | 15 | -7,54E+14 | 9,26E+14 | 9,57E+14 |
| **380** | A0A7M7L6P4 | LOC551664 | Palmitoyl-protein thioesterase 1 | 310 | -3,98E+14 | 9,36E+13 | 9,62E+14 |
| **381** | A0A7M7MGP7 | LOC100576383 | alpha-1,2-Mannosidase | 1 | 2,85E+14 | 9,41E+14 | 9,63E+14 |
| **382** | A0A7M7GZD2 | LOC408568 | Peptidyl-alpha-hydroxyglycine alpha-amidating lyase 1 isoform X4 | 1 | 2,40E+14 | 9,42E+14 | 9,63E+14 |
| **383** | A0A7M6UFV3 | 100302106 | Uncharacterized protein LOC100302106 | 14 | -1,36E+14 | 3,40E+14 | 9,64E+14 |
| **384** | A0A7M7GBS8 | LOC726252 | Titin isoform X3 | 1 | -1,56E+14 | 3,19E+14 | 9,64E+14 |
| **385** | A0A7M7GJM1 | LOC552228 | Renin receptor isoform X2 | 1 | -1,12E+14 | 3,82E+14 | 9,73E+14 |
| **386** | A0A7M7GB31 | 100577576 | Protein obstructor-E | 20 | -1,12E+14 | 4,01E+14 | 9,73E+14 |
| **387** | A0A7M7GQ31 | LOC726818 | Beta-hexosaminidase | 37 | -1,26E+14 | 4,17E+14 | 9,73E+14 |
| **388** | A0A7M7MM38 | LOC411392 | Protein Red | 22 | 4,70E+14 | 9,53E+14 | 9,73E+14 |
| **389** | A0A7M7G0H4 | LOC727116 | Uncharacterized protein LOC727116 | 6 | 3,85E+14 | 9,63E+14 | 9,80E+14 |
| **390** | A0A7M7H3H4 | 725215 | Venom acid phosphatase Acph-1 | 433 | -1,63E+14 | 4,47E+14 | 9,86E+14 |
| **391** | A0A7M7R8D5 | 411542 | Signal recognition particle receptor subunit alpha homolog | 20 | 8,61E+14 | 9,86E+14 | 9,89E+14 |
| **392** | A0A7M7GAZ8 | 409406 | NTF2-related export protein | 1 | 1,25E+14 | 9,86E+14 | 9,89E+14 |
| **393** | Q5BLY5 |  | Venom acid phosphatase Acph-1 | 1 | 1,14E+13 | 9,78E+14 | 9,89E+14 |
| **394** | A0A7M7FZ02 | LOC724703 | Modular serine protease | 320 | -1,07E+14 | 9,82E+14 | 9,89E+14 |
| **395** | A0A7M7H375 | 551031 | Uncharacterized protein LOC551031 | 23 | -1,07E+14 | 9,80E+14 | 9,89E+14 |
| **396** | A0A7M6W880 | MRJP9 | Bee-milk protein | 2026 | -1,36E+14 | 9,75E+14 | 9,89E+14 |
| **397** | A0A7M7FZ07 | LOC726429 | Uncharacterized protein LOC726429 | 38 | 7,60E+14 | 9,90E+14 | 9,90E+14 |

**Supplementary Table 4. Differentially abundant proteins between AF and IV.** Results of differential abundance analysis performed using DEqMS comparing protein abundance profiles between the AF and IV geographic zones. The table includes UniProt accession numbers, gene names, protein descriptions, peptide counts, log fold change (logFC), raw *P* values, and false discovery rate (FDR)-adjusted *P* values. Proteins are ranked according to FDR-adjusted *P* value in ascending order.
